# Synthesis of 5-arylacetylenyl-1,2,4-oxadiazoles and their transformations under superelectrophilic activation conditions

**DOI:** 10.3762/bjoc.17.158

**Published:** 2021-09-15

**Authors:** Andrey I Puzanov, Dmitry S Ryabukhin, Anna S Zalivatskaya, Dmitriy N Zakusilo, Darya S Mikson, Irina A Boyarskaya, Aleksander V Vasilyev

**Affiliations:** 1 Department of Chemistry, Saint Petersburg State Forest Technical University, Institutsky per., 5, Saint Petersburg, 194021, Russia; 2 All-Russia Research Institute for Food Additives – Branch of V.M. Gorbatov Federal Research Center for Food Systems of RAS, Liteyniy pr., 55, Saint Petersburg, 191014, Russia; 3 Institute of Chemistry, Saint Petersburg State University, Universitetskaya nab., 7/9, Saint Petersburg, 199034, Russia

**Keywords:** acetylene-oxadiazoles, Friedel–Crafts reaction, hydroarylation, superelectrophilic activation, triflic acid

## Abstract

Acetylene derivatives of 1,2,4-oxadiazoles, i.e., 5-(2-arylethynyl)-3-aryl-1,2,4-oxadiazoles, have been obtained, for the first time reported, from 5-(2-arylethenyl)-3-aryl-1,2,4-oxadiazoles by their bromination at the carbon–carbon double bond followed by di-dehydrobromination with NaNH_2_ in liquid NH_3_. The reaction of the acetylenyl-1,2,4-oxadiazoles with arenes in neat triflic acid TfOH (CF_3_SO_3_H) at room temperature for 1 h resulted in the formation of *E*/*Z-*5-(2,2-diarylethenyl)-3-aryl-1,2,4-oxadiazoles as products of regioselective hydroarylation of the acetylene bond. The addition of TfOH to the acetylene bond of these oxadiazoles quantitatively resulted in *E*/*Z-*vinyl triflates. The reactions of the cationic intermediates have been studied by DFT calculations and the reaction mechanisms are discussed.

## Introduction

1,2,4-Oxadiazoles have a great importance in chemistry, biology and medicine. Many drugs contain an 1,2,4-oxadiazole ring, such as butalamine [1], libexin [2], ataluren [3], oxolamine [4], and pleconaril [5]. Various oxadiazole derivatives show different kinds of activity against cancer [6–7], tuberculosis [8], Gram-positive bacteria [9], and they are used in treatment of epilepsy [10] and Alzheimer disease [11–13]. The synthesis of compounds of the 1,2,4-oxadiazole series is an actual task in organic and medicinal chemistry (see selected reviews on this topic [14–22]). However, among all the varieties of 1,2,4-oxadiazoles, their acetylenic derivatives are quite rare. To the best of our knowledge, there is only one example of 1,2,4-oxadiazole conjugated with an acetylene bond, which is 3-phenylethynyl-1,2,4-oxadiazole [23]. Up to the moment, there are no data on the preparation of 1,2,4-oxadiazoles containing a conjugated acetylenic substituent in the position 5 of the heterocyclic ring.

Based on our previous works on the chemistry of 1,2,4-oxadiazoles in superacids [24–25], we undertook this study on further investigation of the transformations of these heterocyclic compounds in electrophilic media. The main goals of this work were the synthesis of 5-arylacetylenyl-1,2,4-oxadiazoles and the study of their reactions with/without arenes under the conditions of superelectrophilic activation by the Brønsted superacid CF_3_SO_3_H (TfOH), the strong Lewis acids AlX_3_ (X = Cl, Br), or the acidic zeolite CBV-720.

## Results and Discussion

**Scheme 1:**
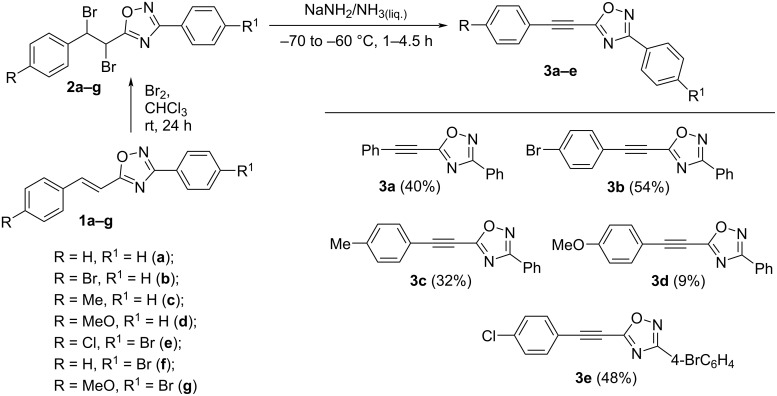
Synthesis of 5–arylethynyl-3-aryl-1,2,4-oxadiazoles **3a–e**.

The synthesis of 5-arylethynyl-1,2,4-oxadiazoles **3** was based on transformations of the corresponding 5-styryloxadiazoles, i.e., 5-(2-arylethenyl)-3-aryl-1,2,4-oxadiazoles **1a–g** (Scheme 1). Bromination of the side chain carbon–carbon double bond in oxadiazoles **1a–g** led to pairs of diastereomers of dibromo derivatives **2a–g**. Then, several bases were tested for the di-dehydrobromination of compounds **2a–g**. However, treatment of **2a–g** in the following systems, KOH–EtOH (reflux, 2 h), BuLi–THF (−40 °C, 2 h), *t*-BuOK–THF (reflux, 2 h), or LiN(iPr)_2_–THF (−40 °C, 2 h), afforded complex mixtures of reaction products without desired acetylenyloxadiazoles **3**. We succeeded to get compounds **3a–e** by the reaction of **2a–e** with sodium amide in liquid ammonia [NaNH_2_–NH_3_(liq.)] only at low temperature −70 to −60 °C (Scheme 1). However, the yields of target compounds were moderate 32–54% (for **3a–c,e**) or even low 9% (for **3d**). Running this reaction at higher temperature –50 to –40 °C led to a decrease of the yields of compounds **3**. Apart from that, compounds **2f**,**g** containing a 3-*para*-bromophenyl moiety in the heterocyclic core gave no corresponding 5-acetylenyloxadiazoles **3** in the system NaNH_2_–NH_3_(liq.), only mixtures of oligomeric materials were formed. Moreover, compound **3e** was obtained as an inseparable mixture with styryloxadiazole **1e**. The latter may be formed from **3e** under the reduction by the solution that contained NaNH_2_. All these data point out the instability of 5-acetylenyloxadiazoles **3** in strong basic and nucleophilic media. Oxadiazoles **3**, which were initially formed from compounds **2** in the system NaNH_2_–NH_3_(liq.), underwent further secondary transformations under nucleophilic reaction conditions, even at very low temperature –70 to –60 °C, that resulted in low to moderate yields of the target acetylene derivatives.

Then, electrophilic reactions of 5-acetylenyloxadiazoles **3a–d** in different acids were studied. In our recent study on the electrophilic activation of 5-styryl-1,2,4-oxadiazoles **1** [24], it was shown by means of NMR spectroscopy and DFT calculation that the protonation of these oxadiazoles in Brønsted superacids TfOH and FSO_3_H gave reactive N,C-diprotonated species. The protonation of oxadiazoles **1** takes place at the nitrogen N4 and the α-carbon of the side chain C=C bond. One would expect the formation of similar dications at the protonation of acetylenyloxadiazoles **3** in Brønsted superacids (see Table 1). Table 1 contains data on DFT calculations of cations **Aa–d** (N-protonated forms) and **Ba–d** (N,C-diprotonated forms) derived at the protonation of oxadiazoles **3a–d**. Charge delocalization, contribution of atomic orbital into LUMO, global electrophilicity indices ω [26–27], and Gibbs free energies of protonation reactions with hydroxonium ion (H_3_O^+^) Δ*G*_298_ were calculated.

**Table 1:** Selected electronic characteristics for cations **Aa–d** and **Ba–d** calculated by DFT from protonation of oxadiazoles **3a–d**.

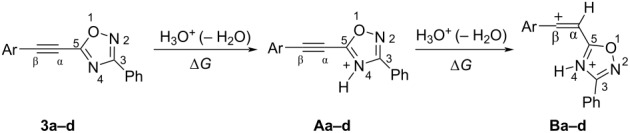								

Species	*E*_HOMO_, eV	*E*_LUMO_, eV	ω,^a^ eV	*q*(C^β^),^b^ e	*k*(C^β^)_LUMO_,^c^ %	*q*(N^2^),^b^ e	*q*(N^4^),^b^ e	Δ*G*_298_ of protonation, kJ/mol

Cations **A** (N-protonated species)								

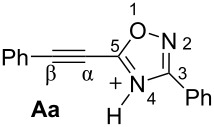	−7.44	−3.56	3.9	0.23	7.2	−0.13	−0.49	−80.8
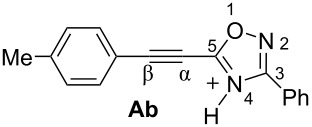	−7.23	−3.49	3.8	0.23	7.4	−0.13	−0.50	−83.4
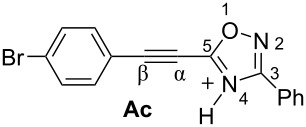	−7.28	−3.62	4.0	0.23	7.4	−0.13	−0.49	−79.2
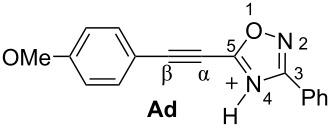	−6.84	−3.38	3.8	−0.30	6.1	−0.14	−0.53	−86.6

Dications **B** (N,C-diprotonated species)								

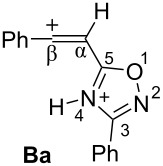	−7.83	−5.28	8.4	0.47	30.0	−0.11	−0.47	+18.0
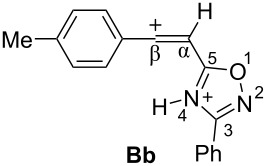	−7.82	−5.04	7.4	0.44	29.0	−0.11	−0.48	−1.2
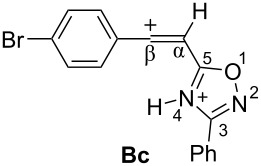	−7.82	−5.25	8.3	0.45	24.3	0.1	0.4	+18.3
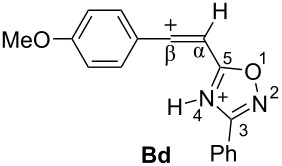	−7.80	−4.63	6.1	0.40	16.7	−0.11	−0.48	−28.8

^a^Global electrophilicity index ω = (*E*_HOMO_ + *E*_LUMO_)^2^/8(*E*_LUMO_ − *E*_HOMO_). ^b^Natural charges. ^c^Contribution of atomic orbital into molecular orbital.

Big negative values of Δ*G*_298_ (−86.6 to −79.2 kJ/mol) of the first protonation step show that the formation of N-protonated species **Aa–d** is extremely energetically favorable. For the second protonation (reaction **A**→**B**) leading to dications **Ba–d**, the Δ*G*_298_ values vary from −28.6 to 18.3 kJ/mol. Although the second protonations are sometimes mildly endergonic (and hence there would be an unfavourable equilibrium between species **A** and **B**), the capture of the diaction **B** by a nucleophile is likely to be very exergonic and this can drive the reaction through to the products. Calculated electronic characteristic of these dications reveal their high electrophilicity, the indexes ω are 6.1–8.4 eV. Carbon C^β^ bears a large positive charge (0.40–0.47 e) and gives a big contribution into LUMO (16.7–30%), pointing out that this carbon is a reactive electrophilic center by charge and orbital factors.

**Scheme 2:**
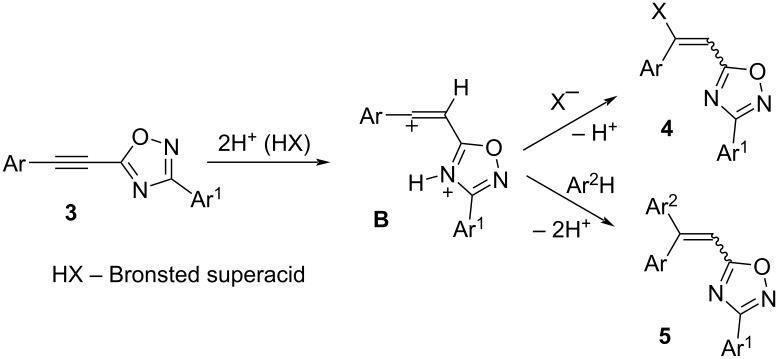
Plausible reaction mechanism for transformations of 5-acetylenyl-1,2,4-oxadiazoles **3** in Brønsted superacids.

Thus, according our previous data on reactions of 5-styryl-1,2,4-oxadiazoles **1** [24] and results of the DFT calculations for protonation of 5-acetylenyl-1,2,4-oxadiazoles **3** (Table 1), one would propose the following reaction pathways for compounds **3** in Brønsted superacids (Scheme 2). Protonation of oxadiazole **3** affords dication **B**, which may react with counter anion of acid X^−^ giving rise to vinyl derivatives **4**. In the presence of nucleophilic arene molecules, species **B** should afford substances **5** as products of hydroarylation of the acetylene bond of the starting compounds **3**.

**Scheme 3:**
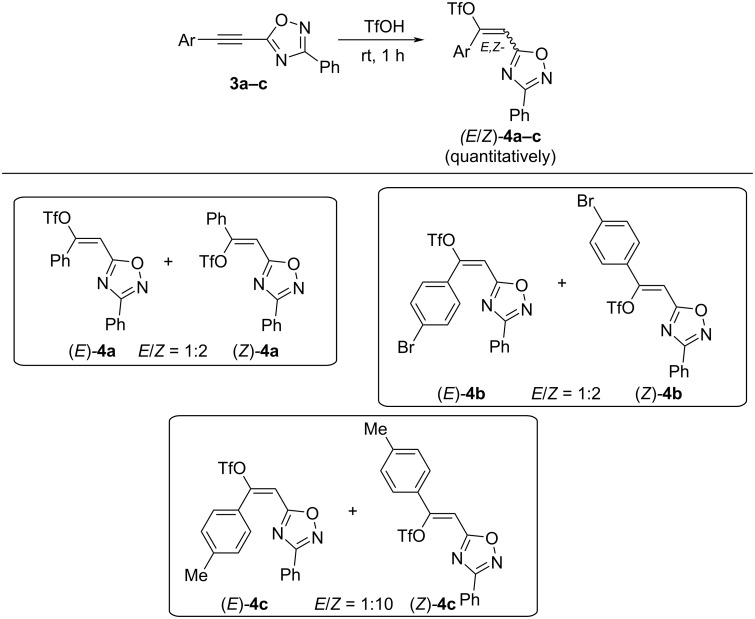
Quantitative formation of *E*/*Z-*vinyl triflates **4a–c** from 5-acetylenyl-1,2,4-oxadiazoles **3a–c** in TfOH.

Indeed, reaction of 5-acetylenyl-1,2,4-oxadiazoles **3a–c** with excess of TfOH at room temperature for 1 h resulted in the quantitative preparation of *E*/*Z*-isomers of vinyl triflates **4a–c** with a predominant formation of *Z*-isomers as product of an *anti-*addition of TfOH to the acetylene bond (Scheme 3). *E*/*Z-*Stereochemistry of compounds **4a–c** was determined by H,F-NOESY correlation between vinyl proton (>C=CH–) and the CF_3_ group from the TfO substituent (see Supporting Information File 1). It should be noted that attempts of chromatographic separation of triflates **4a–c** into individual *E-* and *Z-*isomers on silica gel led to a decrease of their yields and a change in *E*/*Z*-ratio. That reveals instability of these compounds on silica gel.

**Scheme 4:**
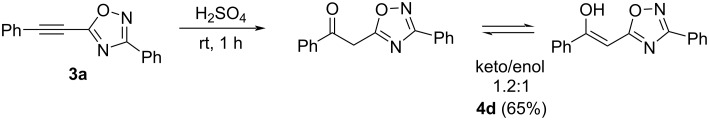
Formation of compound **4d** from 5-acetylenyl-1,2,4-oxadiazole **3a** in H_2_SO_4_.

In the same reaction in H_2_SO_4_ (Scheme 4), oxadiazole **3a** gave the product of hydration of the acetylene bond (**4d**, yield of 65%) existing in solution as equilibrium between ketone and enol forms in a ratio of 1.2:1 according to NMR data (see Supporting Information File 1).

**Scheme 5:**
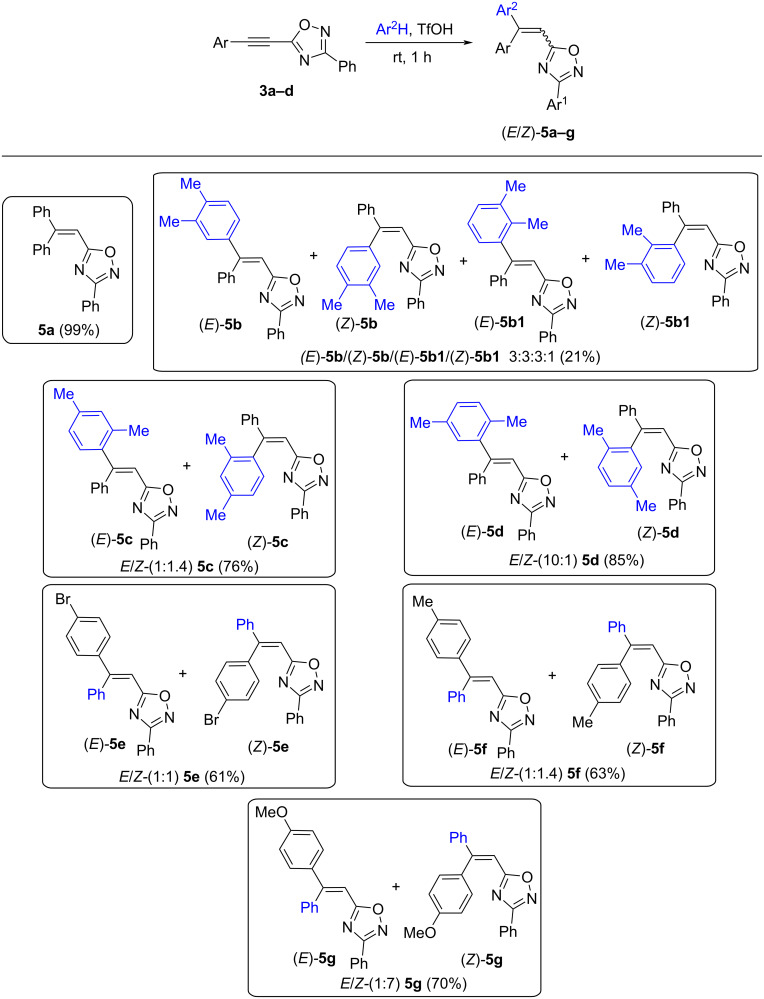
Hydroarylation of 5-acetylenyl-1,2,4-oxadiazole **3a–d** by arenes in TfOH leading to compounds *E*/*Z-***5a–g**.

Then, reactions of 5-acetylenyl-1,2,4-oxadiazole **3a–d** with arenes (benzene and *o*-, *m*-, *p*-xylenes) in TfOH at room temperature for 1 h leading to products of hydroarylation of the acetylene bond, compounds (*E*/*Z*)*-***5a–g**, were carried out (Scheme 5). This reaction gave *E*/*Z-*isomers **5b–g**, their stereochemical configuration was determined by H,H NOESY correlations between the vinyl proton and aromatic protons (see Supporting Information File 1). In the case of the reaction with *o*-xylene, pairs of *E*/*Z-*isomers of two regioisomers, (*E*/*Z*)*-***5b** and (*E*/*Z*)*-***5b1**, were obtained.

We also checked the reaction of oxadiazole **3a** with benzene under the action of Lewis acids AlCl_3_, AlBr_3_ and acidic zeolite CBV-720 (Table 2). However, these Lewis acids showed unsatisfactory results leading to oligomeric materials (Table 2, entries 1 and 2). Probably, due to some secondary reactions of the formed compound **5a** with AlCl_3_, AlBr_3_. The yield of target compound **5a** in the reaction with zeolite was lower than in the same reaction in TfOH (compare entry 3 in Table 2 with data shown in Scheme 5). Thus, among the tested acidic reagents, TfOH showed better results for the hydroarylation of compounds **3**.

**Table 2:** Reactions of 5-acetylenyloxadiazole **3a** with benzene under the action of various acids.

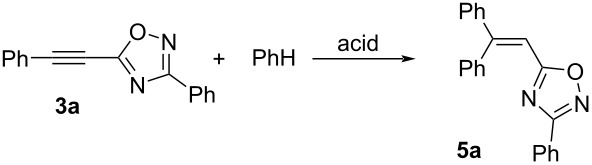				

Entry	Acid	Reaction conditions		Yield of **5a**, %

		Temperature, °C	Time, h	

1	AlCl_3_	rt	1	oligomeric compounds
2	AlBr_3_	rt	1	oligomeric compounds
3	Zeolite CBV-720	130	1	56

Additionally, the reaction of oxadiazole **3a** with benzene in TfOH (rt, 1 h) in the presence of cyclohexane, as a hydride ion source, was conducted to achieve the ionic hydrogenation of intermediate cationic species. However, no products of ionic hydrogenation were obtained, only the product of the hydrophenylation of the acetylene bond **5a** was quantitatively isolated (compare with data shown in Scheme 5).

## Conclusion

For the first time reported, we have synthesized 5-arylacetylene derivatives of 1,2,4-oxadiazoles, i.e., 5-(2-arylethynyl)-3-aryl-1,2,4-oxadiazoles. In the Brønsted superacid TfOH, these oxadiazoles react in a way of electrophilic addition to the acetylene bond. They give products of hydroarylation of the acetylene bond in the reaction with arenes or vinyl triflates in reaction with TfOH without arenes.

## Supporting Information

File 1 Experimental procedures, characterization data and ^1^H and ^13^C NMR spectra of compounds, as well as data of DFT calculations.

## References

[1] Palazzo G, Corsi G (1962). Arzneim Forsch.

[2] Coupar I M, Hedges A, Metcalfe H L, Turner P (1969). J Pharm Pharmacol.

[3] Jones A M, Helm J M (2009). Drugs.

[4] Silvestrini B (1960). Minerva Med.

[5] Rotbart H A, Webster A D (2001). Clin Infect Dis.

[6] Zhang H-Z, Kasibhatla S, Kuemmerle J, Kemnitzer W, Ollis-Mason K, Qiu L, Crogan-Grundy C, Tseng B, Drewe J, Cai S X (2005). J Med Chem.

[7] Shamsi F, Hasan P, Queen A, Hussain A, Khan P, Zeya B, King H M, Rana S, Garrison J, Alajmi M F (2020). Bioorg Chem.

[8] Atmaram Upare A, Gadekar P K, Sivaramakrishnan H, Naik N, Khedkar V M, Sarkar D, Choudhari A, Mohana Roopan S (2019). Bioorg Chem.

[9] Janardhanan J, Chang M, Mobashery S (2016). Curr Opin Microbiol.

[10] Mohammadi-Khanaposhtani M, Shabani M, Faizi M, Aghaei I, Jahani R, Sharafi Z, Shamsaei Zafarghandi N, Mahdavi M, Akbarzadeh T, Emami S (2016). Eur J Med Chem.

[11] Querfurth H W, LaFerla F M (2010). N Engl J Med.

[12] Jiang C-S, Fu Y, Zhang L, Gong J-X, Wang Z-Z, Xiao W, Zhang H-Y, Guo Y-W (2015). Bioorg Med Chem Lett.

[13] Mei W-w, Ji S-s, Xiao W, Wang X-d, Jiang C-s, Ma W-q, Zhang H-y, Gong J-x, Guo Y-w (2017). Monatsh Chem.

[14] Hemming K (2001). J Chem Res, Synop.

[15] Kayukova L A (2005). Pharm Chem J.

[16] Hemming K, Zhdankin V V (2008). 1.2.4-Oxadiazoles. Five-membered Rings: Triazoles, Oxadiazoles, Thiadiazoles and their Fused Carbocyclic Derivatives.

[17] Pace A, Pierro P (2009). Org Biomol Chem.

[18] Bora R O, Dar B, Pradhan V, Farooqui M (2014). Mini-Rev Med Chem.

[19] Pace A, Buscemi S, Piccionello A P, Pibiri I (2015). Adv Heterocycl Chem.

[20] Piccionello A P, Pace A, Buscemi S (2017). Chem Heterocycl Compd.

[21] Aggarwal S, Goyal A, Kaur R (2020). Res J Pharm Technol.

[22] Lelyukh M, Demchuk I, Harkov S, Chaban T, Drapak I, Chaban I, Shelepeten L, Matiychuk V (2020). Biointerface Res Appl Chem.

[23] Claisse J A, Foxton M W, Gregory G I, Sheppard A H, Tiley E P, Warburton W K, Wilson M J (1973). J Chem Soc, Perkin Trans 1.

[24] Zalivatskaya A S, Ryabukhin D S, Tarasenko M V, Ivanov A Y, Boyarskaya I A, Grinenko E V, Osetrova L V, Kofanov E R, Vasilyev A V (2017). Beilstein J Org Chem.

[25] Golushko A A, Khoroshilova O V, Vasilyev A V (2019). J Org Chem.

[26] Parr R G, Szentpály L v, Liu S (1999). J Am Chem Soc.

[27] Chattaraj P K, Giri S, Duley S (2011). Chem Rev.

