# Supplementary material for: Synthesis of 5-arylacetylenyl-1,2,4-oxadiazoles and their transformations under superelectrophilic activation conditions
Source: Beilstein J Org Chem. 2021 Sep 15;17:2417–24. doi: 10.3762/bjoc.17.158 (PMC8450969; doi:10.3762/bjoc.17.158)
Supplement: File 1 — Experimental procedures, characterization data and 1H and 13C NMR spectra of compounds, as well as data of DFT calculations. [file Beilstein_J_Org_Chem-17-2417-s001.pdf]

BEILSTEIN JOURNAL OF ORGANIC CHEMISTRY

Supporting Information

for

Synthesis of 5-arylacetylenyl-1,2,4-oxadiazoles and their transformations under superelectrophilic activation conditions

Andrey I. Puzanov, Dmitry S. Ryabukhin, Anna S. Zalivatskaya, Dmitriy N. Zakusilo, Darya S. Mikson, Irina A. Boyarskaya and Aleksander V. Vasilyev

Beilstein J. Org. Chem. **2021,** *17,* 2417–2424. doi:10.3762/bjoc.17.158

Experimental procedures, characterization data and ^{1}H and ^{13}C NMR spectra of compounds, as well as data of DFT calculations

License and Terms: This is a supporting information file under the terms of the Creative Commons Attribution License (https://creativecommons.org/licenses/by/4.0). Please note that the reuse, redistribution and reproduction in particular requires that the author(s) and source are credited and that individual graphics may be subject to special legal provisions.
The license is subject to the *Beilstein Journal of Organic Chemistry* terms and conditions: (https://www.beilstein-journals.org/bjoc/terms)

Contents

1. General……………………………………………………………………………….………..S2

2. Synthesis and characterization of compounds **2–5**……………………………………………S2

3. ^{1}H and ^{13}C NMR spectra of compounds **2–5** ………………………………………………..S12

4. Data of DFT calculation of compounds **3** and cations **A, B**…………………………………S45

1. General

NMR spectra of solutions of compounds in $CDCl_3$ were recorded on Bruker AVANCE 500 or Bruker AVANCE III 400 spectrometers (at 500, 400 and 125, 100 MHz for ^1H and ^{13}C NMR spectra respectively) at 25 °C. The solvent residual signals $CDCl_3$ (δ 7.26 ppm) for ^1H NMR spectra and the carbon signal of $CDCl_3$ (δ 77.16 ppm) for ^{13}C NMR spectra were used as references. HRMS was carried out at instrument Bruker maXis HRMS–ESI–QTOF. GC–MS study was carried out on Agilent Technology instrument. The preparative reactions were monitored by thin-layer chromatography carried out on silica gel plates (Silufol UV-254), using UV light for detection. Column chromatography was performed on silica gel Merck-60 with petroleum ether–ethyl acetate mixture eluation.

2. Synthesis and characterization of compounds 2–5

General procedure for the synthesis of 5-(1,2-dibromo-2-arylethyl)-3-aryl-1,2,4-oxadiazoles 2.

A solution of Br_2 with a volume of 0.115 ml (2.1 mmol) dissolved in 15 ml of $CHCl_3$ was added dropwise to a solution of the starting 1,2,4-oxadiazole **1** (2 mmol) in 5 ml of $CHCl_3$ at room temperature with a magnetic stirring. The reaction was carried out for 24 h. Evaporation of solvent in vacuum gave pure reaction products. Compounds **2a,c,e,f** were obtained as mixtures of two diastereomers (see below). A ratio of diastereomers was determined by ^1H NMR as a ratio of the signals of the corresponding protons in the structural fragment –CH(Br)–CH(Br)–.

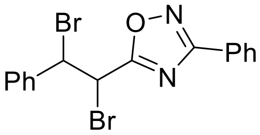

5-(1,2-Dibromo-2-phenylethyl)-3-phenyl-1,2,4-oxadiazole (**2a)** was obtained as a mixture of two diastereomers in a ratio of ~ 10 : 1. Yield of 90%. Orange solid, mp 168-170°C. Selected signals for D1: ^1H NMR (400 MHz, $CDCl_3$) δ 5.68 (d, *J* = 11.8 Hz, 1H), 5.73 (d, *J* = 11.8 Hz, 1H), 7.42-7.48 (m, 3H), 7.52-7.54 (m, 5H), 8.16 (d, *J* = 8.0 Hz,2H).^{13}C NMR (100 MHz, $CDCl_3$) δ 40.1, 51.2, 126.3, 127.8, 128.1, 129.1, 129.2, 129.8, 131.7, 137.7, 169.1, 176.2. Selected signals for D2: ^1H NMR (400 MHz, $CDCl_3$) δ 4.89 (d, *J* = 11.7 Hz, 1H), 5.35 (d, *J* = 11.7 Hz, 1H). ^{13}C NMR (100 MHz, $CDCl_3$) δ 46.6, 50.5. Signals for D1 and D2: ^1H NMR (400 MHz, $CDCl_3$) δ 7.42-7.48 (m, 3H), 7.52-7.54 (m, 5H), 8.16 (d, *J* = 8.0 Hz,2H). ^{13}C NMR (100 MHz, $CDCl_3$) δ 126.3, 127.8, 128.1, 129.1, 129.2, 129.8, 131.7, 137.7, 169.1, 176.2. HRMS: $C_{16}H_{13}Br_2N_2O$ found 406.9389 $[M+H]^+$, calcd. 406.9389.

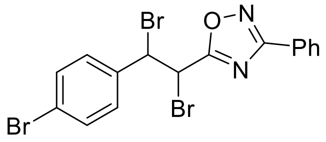

5-(1,2-Dibromo-2-(4-bromophenyl)ethyl)-3-phenyl-1,2,4-oxadiazole (2b) was obtained only one diastereomer. Yield of 82%. Orange solid, mp 170-172°C. ^{1}H NMR (500 MHz, $CDCl_3$) δ 5.63 (d, *J*= 11.9 Hz, 1H), 5.65 (d, *J*= 11.9 Hz, 1H), 7.39 (d, *J*= 8.4 Hz, 2H), 7.50-7.55 (m, 3H), 7.59 (d, *J*= 8.4 Hz, 2H), 8.14 (d, *J*= 9.3 Hz, 2H).^{13}C NMR (100 MHz, $CDCl_3$) δ 39.8, 50.2, 123.9, 126.3, 127.8, 129.1, 129.7, 131.8, 132.5, 136.8, 169.1, 175.9. HRMS: found $C_{16}H_{12}Br_3N_2O$ 484.8495 $[M+H]^+$, calcd. 484.8494.

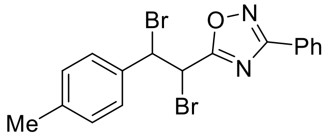

5-(1,2-Dibromo-2-(4-methylphenyl)ethyl)-3-phenyl-1,2,4-oxadiazole (2c) was obtained as a mixture of two diastereomers in a ratio of ca. 2 : 1. Yield of 90%. Orange solid, mp 120-122°C (for mixture of diastereomers). Selected signals for D1: ^{1}H NMR (500 MHz, $CDCl_3$) δ 2.41 (s, 3H, CH_3), 5.67 (d, *J*=11.8 Hz, 1H), 5.73 (d, *J*=11.8 Hz, 1H), 7.27 (d, *J*=4.6 Hz, 2H), 7.41 (d, *J*=7.95 Hz, 2H), 7.51-7.56 (m, 4H), 8.16 (d, *J*=6.65 Hz, 2H).^{13}C NMR (125 MHz, $CDCl_3$) δ 40.1, 51.2. Selected signals for D2: ^{1}H NMR (500 MHz, $CDCl_3$) δ 2.38 (s, 2H, CH_3), 4.88 (d, *J*=11.8 Hz, 1H), 5.34 (d, *J*=11.7 Hz, 1H), 7.21 (d, *J*=8 Hz, 1H), 7.31 (d, *J*=8 Hz, 1H). ^{13}C NMR (125 MHz, $CDCl_3$) δ 46.8, 50.6. Signals for D1 and D2: ^{13}C NMR (125 MHz, $CDCl_3$) δ 126.3, 127.8, 128.1, 129.1, 129.2, 129.8, 131.7, 137.7, 169.1, 176.2. HRMS: $C_{17}H_{15}Br_2N_2O$ found 420.9546 $[M+H]^+$, calcd. 420.9546.

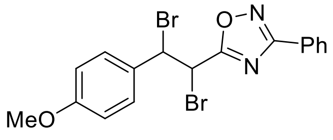

5-(1,2-Dibromo-2-(4-methoxyphenyl)ethyl)-3-phenyl-1,2,4-oxadiazole (2d) was obtained only one diastereomer. Yield of 72%. Orange solid, mp 139-141°C.^{1}H NMR (500 MHz, $CDCl_3$) δ 3.85 (s, 3H, CH_3), 5.68 (d, *J*=11.9 Hz, 1H), 5.71 (d, *J*=11.9 Hz, 1H), 6.96 (d, *J*=8.5 Hz, 2H), 7.44 (d, *J*=8.5 Hz, 2H), 7.50-7.55 (m, 3H), 8.15 (d, *J*=7.8 Hz, 2H).^{13}C NMR (125 MHz, $CDCl_3$) δ 40.5, 51.6, 55.5, 114.6, 126.4, 127.8, 129.1, 129.5, 131.7, 160.6, 169.1, 176.3. HRMS: found $C_{17}H_{15}Br_2N_2O_2$ 436.9500 $[M+H]^+$, calcd. 436.9505.

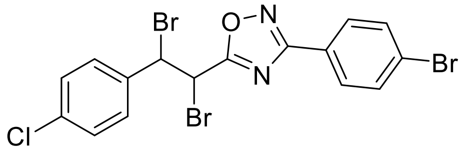

3-(4-Bromophenyl)-5-(1,2-dibromo-2-(4-chlorophenyl)ethyl)-1,2,4-oxadiazole (2e) was obtained as a mixture of two diastereomers in a ratio of ~ 4 : 1. Yield of 92%. Orange solid, mp 171-173°C (for mixture of diastereomers). Selected signals for D1: ^{1}H NMR (500 MHz, $CDCl_3$) δ 5.62 (d, *J*=11.8 Hz, 1H), 5.65(d, *J*=11.8 Hz, 1H), 7.35-7.39 (m, 1H), 7.42-7.76(m, 4H), 7.66 (d, *J*=8.5 Hz,2H), 8.01 (d, *J*=8.5 Hz,2H). ^{13}C NMR (125 MHz, $CDCl_3$) δ 39.8, 50.1. Selected signals for D2: ^{1}H NMR (500 MHz, $CDCl_3$) δ 4.81 (d, *J*=11.7 Hz, 1H), 5.3 (d, *J*=11.7 Hz, 1H). ^{13}C NMR (125 MHz, $CDCl_3$) δ 46.4, 49.3. Signals for D1 and D2: ^{13}C NMR (125 MHz, $CDCl_3$) δ 125.2, 126.4, 129.2, 129.4, 129.5, 129.54, 129.6, 132.4, 135.5, 135.8, 136.1, 136.2, 168.4, 176.2. HRMS: found $C_{16}H_{11}Br_3ClN_2O$ 518.8105 $[M+H]^+$, calcd. 518.8105.

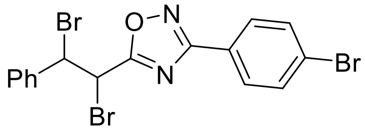

3-(4-Bromophenyl)-5-(1,2-dibromo-2-phenylethyl)-1,2,4-oxadiazole (2f) was obtained as a mixture of two diastereomers in a ratio of ~ 1 : 1. Yield of 94%. Orange solid, mp 141-143°C (for mixture of diastereomers). Selected signals for D1: ^{1}H NMR (500 MHz, $CDCl_3$) δ 5.65 (d, *J*=11.8 Hz, 1H), 5.71 (d, *J*=11.8 Hz, 1H. ^{13}C NMR (125 MHz, $CDCl_3$) δ 39.9, 46.5. Selected signals for D2: ^{1}H NMR (500 MHz, $CDCl_3$) δ 4.88 (d, *J*=11.7 Hz, 1H), 5.33 (d, *J*=11.7 Hz, 1H). ^{13}C NMR (125 MHz, $CDCl_3$) δ 50.2, 51.2. Signals for D1 and D2: ^{1}H NMR (500 MHz, $CDCl_3$) δ 7.37-7.47 (m, 11H), 7.52 (d, *J*=8.2 Hz, 2H), 7.65-7.67 (m, 4H), 7.71 (d, *J*=8.5 Hz, 1H), 8.01-8.05 (m, 3H), 8.08 (d, *J*=8.55 Hz, 1H). ^{13}C NMR (125 MHz, $CDCl_3$) δ 125.3, 126.4, 128.1, 128.2, 129.1, 129.3, 129.6, 129.7, 129.9, 132.3, 132.4, 132.7, 137.5, 137.6, 171.9, 176.5. HRMS: found $C_{16}H_{12}Br_3N_2O$ 484.8494 $[M+H]^+$, calcd. 484.8494.

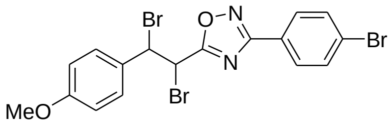

3-(4-Bromophenyl)-5-(1,2-dibromo-2-(4-methoxyphenyl)ethyl)-1,2,4-oxadiazole (2g) was obtained only one diastereomer. Yield of 97%. Orange solid, mp 135-137°C.^{1}H NMR (500 MHz, $CDCl_3$) δ 3.85 (s, 3H, OMe), 5.66-5.71 (m, 2H), 6.69 (d, *J*=8 Hz, 2H), 7.44 (d, *J*=8 Hz, 2H), 7.65 (d, *J*=8 Hz, 2H), 8.01 (d, *J*=8 Hz,

2H).^{13}C NMR (125 MHz, $CDCl_3$) δ 40.2, 51.4, 55.4, 114.4, 125.1, 126.2, 129.1, 129.3, 132.2, 160.4, 168.2, 176.4. HRMS: found $C_{17}H_{14}Br_3N_2O_2$ 514.8605[M+H]$^+$, calcd. 514.8600.

General procedure for the synthesis of 5-arylethynyl-1,2,4-oxadiazoles 3.

First, sodium amide was obtained by condensation of gaseous ammonia at −60 to −70 °C and $Fe(NO_3)_3{\cdot}9H_2O$ (3mg, 0.007 mmol) was added with stirring, and then, after the formation of a pale brown solution, metal sodium (168 mg, 7.3 mmol) was added in small portions. The result was a dark blue sodium amide solution. Then 5-(1,2-dibromo-2-arylethyl)-3-aryl-1,2,4-oxadiazole **2** (2.4 mmol) was added. The reaction was running at −60 to −70 °C for 1–4.5 h. At the end of the reaction, gaseous ammonia was evaporated at room temperature to obtain a precipitate, which was filtered and washed with water several times. The resulting solid was recrystallized from ethanol or mixture of methanol/benzene 3:1, vol. Additional purification was carried out by column chromatography on silica gel with eluation by petroleum ether–ethyl acetate 95: 5, vol.

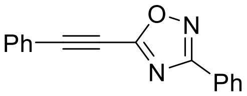

3-Phenyl-5-(phenylethynyl)-1,2,4-oxadiazole (3a). Yield of 40%. White solid, mp 130-132°C.^{1}H NMR (400 MHz, $CDCl_3$)δ 7.44-7.52 (m, 6H), 7.68 (d, *J*=7.4 Hz, 2H), 8.13 (d, *J*=6.4 Hz, 2H).^{13}C NMR (100 MHz, $CDCl_3$) δ 73.9, 98.2, 119.6, 126.4, 127.6, 128.9, 129.1, 131.2, 131.6, 132.8, 161.5, 169.0. GC-MS, m/z, $I_{rel.}$, %: 246 [M+] (100); 129 (12); 119 (100); 91 (15); HRMS: found $C_{16}H_{10}N_2NaO$ 269.0685 [M+Na]$^+$, calcd. 269.0685.

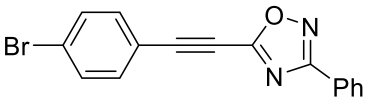

5-((4-Bromophenyl)ethynyl)-3-phenyl-1,2,4-oxadiazole (3b). Yield of 54%. Beige solid, mp 151-153°C. ^{1}H NMR (500 MHz, $CDCl_3$) δ 7.50-7.60 (m, 5H), 7.59 (d, *J*=8.3 Hz, 2H), 8.12 (d, *J*=6.7 Hz, 2H).^{13}C NMR (125 MHz, $CDCl_3$) δ 74.9, 96.9, 118.4, 126.1, 126.3, 127.7, 129.1, 131.7, 132.4, 134.1, 161.3, 169.1. IR ($CHCl_3$), ν, cm^{-1}: 2230 (C≡C); GC-MS, m/z, $I_{rel.}$, %: 326 (M^{+2}) (60); 324 (M^+) (60); 207 (20); 191 (11); 119 (100); 91 (16); HRMS: found $C_{16}H_{10}BrN_2O$ 324.9971 [M+H]$^+$, calcd. 324.9971.

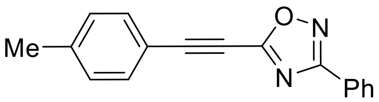

5-((4-Methylphenyl)ethynyl)-3-phenyl-1,2,4-oxadiazole (3c). Yield of 32%. White solid, mp 137-139°C. ^{1}H NMR (400 MHz, $CDCl_3$) δ 2.41 (s, 1H, CH_3), 7.24 (d, *J*=6.4 Hz, 2H), 7.48-7.53 (m, 3H), 7.57 (d, *J*=6.4 Hz,2H), 8.12-8.14 (m, 2H).^{13}C NMR

(100 MHz, $CDCl_3$) δ 21.9, 73.6, 98.7, 116.5, 126.5, 127.6, 129.1, 129.7, 131.6, 132.8, 141.9, 161.7, 169.0. GC-MS, m/z, $I_{rel.}$, %: 260 [M+] (100); 119 (50); 141 (48); 261 (20); HRMS: found $C_{17}H_{13}N_2O$ 261.1028 $[M+H]^+$, calcd. 261.1023.

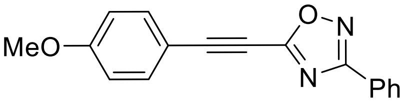

5-((4-Methoxyphenyl)ethynyl)-3-phenyl-1,2,4-oxadiazole (3d). Yield of 9%. White solid, mp 114-116°C.^{1}H NMR (400 MHz, $CDCl_3$) δ 3.86 (s, 1H, OMe), 6.94 (d, *J* = 6.9 Hz, 2H), 7.48-7.53 (m, 3H), 7.62 (d, *J* = 6.9 Hz,2H), 8.12 (d, *J* = 6.0 Hz,2H).^{13}C NMR (100 MHz, $CDCl_3$) δ55.6, 73.4, 99.0, 111.4, 114.2, 114.6, 126.5, 127.6, 129.1, 131.6, 134.7, 162.0, 169.0. GC-MS, m/z, $I_{rel.}$, %: 276 [M+] (100); 157 (96); 159 (65); 277 (20). HRMS: found $C_{17}H_{13}N_2O_2$ 277.0977 $[M+H]^+$, calcd. 277.0973.

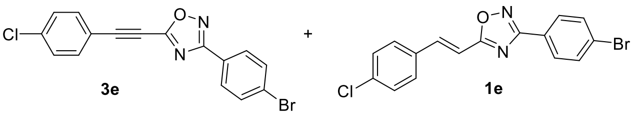

4:1

3-(4-bromophenyl)-5-((4-chlorophenyl)ethynyl)-1,2,4-oxadiazole (3e) and 3-(4-bromophenyl)-5-(4-chlorostyryl)-1,2,4-oxadiazole (1e). Yield of 60%. Ratio **3e**/**1e** 4:1. Orange solid, mp 149-151°C (for mixture of compounds). Selected signals of **3e** from the spectrum of the mixture: ^{1}H NMR (500 MHz, $CDCl_3$) δ 7.40-7.43 (m, 2H), 7.53(d, *J*=8.4 Hz, 1H), 7.60-7.65 (m, 4H), 7.97 (d, *J*=8.4 Hz, 2H). ^{13}C NMR (125 MHz, $CDCl_3$) δ 74.6, 97.2. Spectrum of the mixture: ^{13}C NMR (125 MHz, $CDCl_3$) δ 110.7, 117.9, 125.2, 126.3, 129.1, 129.2, 129.4, 129.5, 132.3, 132.4, 134.0, 136.8, 137.8, 141.6, 161.4, 168.4. HRMS: found $C_{16}H_9BrClN_2O$ 358.9580 $[M+H]^+$, calcd. 358.9581.

General procedure for the synthesis of (*E*-/*Z*-)-5-(trifluoromethylsulfonyloxy-2-arylethenyl)-3-aryl-1,2,4-oxadiazoles 4.

The starting 5-arylethynyl-1,2,4-oxadiazole **3** (0.12 mmol) was added to 1 ml (11 mmol) of trifluoromethanesulfonic acid at room temperature with a stirring. After 1 h, the reaction mixture was poured into 50 ml of water, neutralized with $NaHCO_3$ and extracted with chloroform (3 × 25 ml). The combined extracts were dried with Na_2SO_4, the solvent was distilled off in a vacuum, that gave quantitatively mixtures of *E*-/*Z*-isomers of triflates **4a–c** in ratios as indicated below. Attempts to separate *E*-/*Z*-isomers of triflates **4a–c** by column chromatography on silica gel (eluation by petroleum ether–ethyl acetate 95:5, vol.) led to a dramatic decrease of their yields and a change of *E*-/*Z*- ratios.

Compound **4d** was obtained in the same way in H_2SO_4.

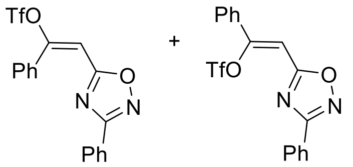

E-/Z-=1:2

(*E-/Z*)-5-(2-Trifluoromethylsulfonyloxy-2-phenylethenyl)-3-phenyl-1,2,4-oxadiazol (Z-4a). Quantitative yield with a ratio of *E-/Z*-isomers 2 : 1. Orange solid, mp 74-76°C (for mixture of *E-/Z*-isomers). Selected signals for *E*-isomer: ^{1}HNMR (400 MHz, $CDCl_3$) δ 6.84 (s, 1H, =CH), 7.97-7.99 (m, 1H). ^{19}F NMR (376 MHz, $CDCl_3$) δ -73.5. Selected signals for *Z*-isomer: ^{1}H NMR (400 MHz, $CDCl_3$) δ 6.88 (s, 1H, =CH), 7.51-7.57 (m, 6H), 7.70 (d, *J*=7 Hz, 2H), 8.18-8.19 m (m, 2H). ^{19}F NMR (376 MHz, $CDCl_3$) δ-73.34. Signals for *E-/Z*-isomers: ^{13}C NMR (100 MHz, $CDCl_3$) δ104.2, 104.4, 118.5 (q, *J*=255Hz), 126.6, 126.8, 127.5, 127.8, 128.8, 129.1, 129.4, 129.5, 131.6, 132.3, 132.4, 154.5, 154.6, 168.9, 169.1, 170.7, 170.9. GC-MS, m/z, $I_{rel.}$, %: 396 [M+] (49); 395 (100); 276 (20); 207 (59); 105 (43); 77 (79). HRMS: found $C_{17}H_{12}F_3N_2O_4S$ 397.0464 $[M+H]^+$, calcd. 397.0464.

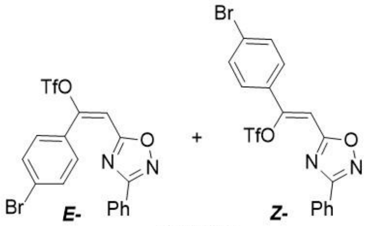

E-/Z-=1:1

(*E-/Z*)-5-(2-Trifluoromethylsulfonyloxy-2-(4-bromophenyl)ethenyl)-3-phenyl-1,2,4-oxadiazol (*E-/Z*-4b). Quantitative yield with a ratio of *E-/Z*-isomers 2 : 1. Orange oily substance. Selected signals for *E*-isomer: ^{1}HNMR (400 MHz, $CDCl_3$)δ 6.87 (s, 1H, =CH). ^{19}F NMR (376 MHz, $CDCl_3$) δ-73.36. Selected signals for *Z*-isomer: ^{1}HNMR (400 MHz, $CDCl_3$) δ 6.85 (s, 1H, =CH). ^{19}F NMR (376 MHz, $CDCl_3$) δ -73.21 Signals for *E-/Z*-isomers: 7.45-7.60 (m, 12H), 7.67-7.70 (m, 4H), 7.99-8.02 (m, 2H), 8.19-8.21(m, 2H). ^{13}C NMR (100 MHz, $CDCl_3$) δ104.7, 106.9, 118.5 (q, CF_3, *J*=255Hz), 126.2, 126.5, 127.1, 127.3, 127.5, 127.7, 128.2, 129.1, 131.0, 131.7, 131.8, 132.2, 132.8, 153.4, 156.2, 168.9, 169.2, 170.4, 170.6. HRMS: found $C_{17}H_{11}BrF_3N_2O_4S$ 474.9570 $[M+H]^+$, calcd. 474.9570.

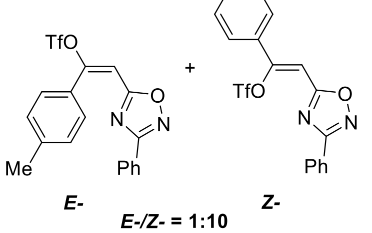

E-/Z- = 1:10

(*E-/Z*)-5-(2-Trifluoromethylsulfonyloxy-2-(4-methylphenyl)ethenyl)-3-phenyl-1,2,4-oxadiazol (*E-/Z*-4c). Quantitative yield with a ratio of

E-/Z-isomers 2 : 1. Orange solid, mp 80-82°C (for mixture of *E-/Z*-isomers). Selected signals for *E*-isomer: ^{1}HNMR (400 MHz, $CDCl_3$)δ 2.45 (s, 3H, CH_3), 6.83 (s, 1H, =CH); ^{13}C NMR (100 MHz, $CDCl_3$) δ21.8, 105.6, 118.4 (q, CF_3, *J*=319 Hz).^{19}F NMR (376 MHz, $CDCl_3$) δ -73.55 (s). Selected signals for *Z*-isomer: ^{1}HNMR (400 MHz, $CDCl_3$) δ 2.44 (s, 3H, CH_3), 6.83 (s, 1H, =CH), 7.31 (d, *J*=8 Hz, 2H), 7.48-7.53 (m, 3H), 7.58 (d, *J* = 8 Hz, 2H), 8.17-8.2 (m, 2H).^{13}C NMR (100 MHz, $CDCl_3$) δ 21.7, 103.2, 118.4 (q, CF_3, *J* = 319 Hz), 120.1, 126.6, 126.7, 127.7, 129.1, 129.2, 130.1, 131.5, 143.1, 154.8, 169.0, 170.8. ^{19}F NMR (376 MHz, $CDCl_3$) δ -73.31. HRMS: found $C_{18}H_{14}F_3N_2O_4S$ 411.0626 $[M+H]^+$, calcd. 411.0621.

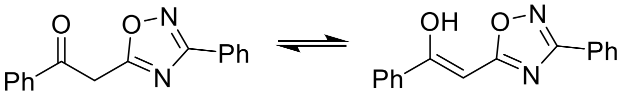
1-Phenyl-2-(3-phenyl-1,2,4-oxadiazole-5-el)ethan-1-опи (Z-)-1-phenyl-2-(3-phenyl-1,2,4-oxadiazole-5-el)ethen-1-ol (4d). Yield of 65%. Orange solid, mp109-110°C. Selected signals for ketone form: ^{1}HNMR (500 MHz, $CDCl_3$) δ 4.7 (s, 2H, CH_2).^{13}C NMR (125 MHz, $CDCl_3$) δ37.5 (s, CH_2). Selected signals for enole form: ^{1}HNMR (500 MHz, $CDCl_3$) δ 6.3 (s, 1H, =CH), 11.9 (s, 1H, OH).^{13}C NMR (125 MHz, $CDCl_3$) δ 81.7 (s, =CH); Signals for ketone and enole: ^{1}HNMR (500 MHz, $CDCl_3$)δ 7.43-7.5 m, 7.6-7.66 m, 7.87-7.9 m, 8.02 (d, *J* = 7.4 Hz), 8.08-8.1 m.^{13}C NMR (125 MHz, $CDCl_3$) δ 29.8, 109.5, 110.4, 126.3, 126.7, 127.6, 128.1, 128.4, 128.5, 128.7, 128.8, 128.9, 129.0, 129.1, 129.2, 129.6, 129.9, 130.6, 131.3, 131.4, 131.5, 131.52, 134.3, 142.8, 166.2, 166.9, 168.8, 173.6, 176.4, 191.1. GC-MS, m/z, $I_{rel.}$, %: 264 [M+] (15); 118 (12); 105 (100); 77 (37). HRMS: found $C_{16}H_{13}N_2O_2$ 265.0972$[M+H]^+$, calcd.265.0972.

General procedure for the synthesis of 3-aryl -5-(2,2-diarylethenyl)-1,2,4-oxadiazoles 5.
5-Arylethynyl-1,2,4-oxadiazole **3** (0.12 mmol) was added to a mixture of arene (1.1 mmol) and TfOH (1 ml, 11 mmol). The reaction mixture was stirred at room temperature for 1 h. Then it was poured into 50 ml of water, neutralized with $NaHCO_3$ and extracted with chloroform (3 × 25 ml). The combined extracts were dried with Na_2SO_4, the solvent was distilled off in a vacuum. The residue was purified by column chromatography on silica gel with eluation by petroleum ether–ethyl acetate 95:5, vol.

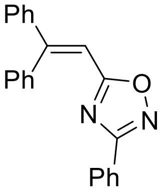
5-(2,2-Diphenylethenyl)-3-phenyl-1,2,4-oxadiazole (5a). Yield of 99%. White solid, mp 110-112°C.^{1}HNMR (500 MHz, $CDCl_3$) δ 7.05 (s, 1H, =CH), 7.29-7.31 (m, 2H), 7.38-7.47 (m, 11H), 7.97 (d, *J*=7 Hz, 2H).^{13}C NMR (125 MHz, $CDCl_3$) δ 109.5, 127.1, 127.5, 128.4,

128.5, 128.7, 128.9, 129.0, 129.6, 129.9, 131.1, 138.3, 140.6, 155.9, 168.3, 174.7. GC-MS, m/z, $I_{rel.}$,%: 324 [M+] (90); 282 (50); 204 (70); 193 (100); 178 (87); 165 (66). HRMS: found $C_{22}H_{17}N_2O$ 325.1335 $[M+H]^+$, calcd. 325.1335.

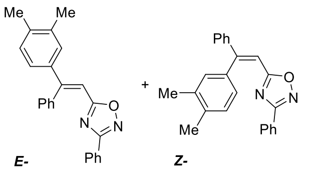

E-/Z-= 1:1

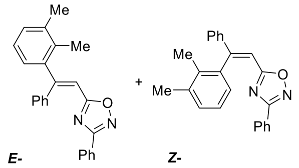

E-/Z-= 1:1

(*E*-/*Z*-)-5-(2-(3,4-Dimethylphenyl)-2-phenylethenyl)-3-phenyl-1,2,4-oxadiazole (*E*-/*Z*-5b:*E*-/*Z*-5b1). Four isomers were obtained in a ratio of 3:3:3:1 in a yield of 21%. Yellow oily substances. The assignment of spectral signals of each isomer was not clear. ^{1}HNMR (400 MHz, $CDCl_3$) δ signals of group CH_3: 2.02, 2.19, 2.20, 2.26, 2.27, 2.30, 2.34, 2.35 (ratio 3:1:1:3:3:3:3:3).^{1}HNMR (400 MHz, $CDCl_3$) δ signals H_{arom}: 6.98, 7.02, 7.04, 7.05, 7.12-7.22, 7.27-7.30, 7.39-7.46, 7.94-7.96, 7.97-7.98. ^{13}C NMR (100 MHz, $CDCl_3$) δ, all signals: 16.5, 19.4, 19.8, 19.9, 19.94, 20.0, 20.6, 108.5, 109.2, 109.9, 125.7, 126.2, 126.8, 127.2, 127.3, 127.4, 127.46, 127.5, 127.6, 127.7, 128.3, 128.6, 128.65, 128.8, 128.88, 128.9, 129.5, 129.6, 129.8, 129.9, 130.00, 130.1, 131.1, 134.5, 135.7, 136.5, 137.0, 137.3, 137.6, 137.8, 138.2, 138.5, 139.1, 139.5, 141.2, 155.7, 156.1, 156.4, 168.2, 174.7, 174.9. HRMS: found $C_{24}H_{21}N_2O$ 353.1654 $[M+H]^+$, calcd. 353.1650.

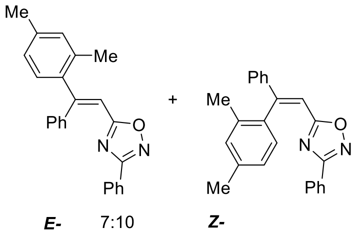

(*E*-/*Z*-)-5-(2-(2,4-Dimethylphenyl)-2-phenylethynyl)-3-phenyl-1,2,4-oxadiazole (*E*-/*Z*-5c). Yield of 76%, ratio of *E*-/*Z*-isomers 1:1.4. Yellow oily substances. ^{1}HNMR (500 MHz, $CDCl_3$) δ 2.05 (s, 3H, CH_3), 2.4 (s, 3H, CH_3), 7.03 (d, *J*=7 Hz,1H), 7.07-7.1 (m, 1H), 7.13 (s, 1H), 7.2 (s, 1H), 7.3-7.5 (m, 14H), 7.98 (d, *J*= 7 Hz, 3H).^{13}C NMR (125 MHz, $CDCl_3$) δ 19.6, 19.9, 21.4, 106.4, 109.8, 110.0, 110.4, 126.7, 127.4, 127.6, 128.8, 128.9, 129.0, 129.9, 131.1, 131.3, 155.3, 168.2, 174.8. GC-MS, m/z, $I_{rel.}$, %: 352 M^+ (10); 335 (100); 232 (21); 218 (40); 191 (38); 178 (23). HRMS: found $C_{24}H_{21}N_2O$ 353. 1648 $[M+H]^+$, calcd. 353.1648.

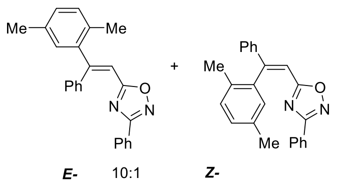

(*E*-/*Z*-)-5-(2-(2,5-Dimethylphenyl)-2-phenylethenyl)-3-phenyl-1,2,4-oxadiazole (*E*-/*Z*-5d). Yield of 85%, ratio of *E*-/*Z*-isomers 10:1. Yellow oily substances. Selected signals for *E*-isomer: ^{1}HNMR (500 MHz, $CDCl_3$) δ 2.33 (s, 3H, CH_3), 6.94 (s, 1H, =CH). Selected signals for *Z*-isomer: ^{1}HNMR (500 MHz, $CDCl_3$) δ 2.06 (s, 3H, CH_3), 6.85 (s, 1H, =CH). Signals for *E-/Z*-isomers: ^{1}HNMR (500 MHz, $CDCl_3$) δ 7.1-7.2 (m, 3H), 7.4-7.5 (m, 10H), 7.98 (d, *J*=7 Hz, 2H). ^{13}C NMR (125 MHz, $CDCl_3$) δ 19.2, 21.1, 106.4, 109.8, 110.4, 127.4, 127.6, 128.8, 129.4, 129.5, 129.9, 130.3, 131.1, 132.8, 135.4, 137.6, 139.3, 155.2, 168.2, 174.4. GC-MS, m/z, $I_{rel.}$, %: 352 [M+] (9); 335 (100); 232 (21); 220 (41); 191 (52); 178 (30). HRMS: found $C_{24}H_{21}N_2O$ 353.1648 $[M+H]^+$, calcd. 353.1648.

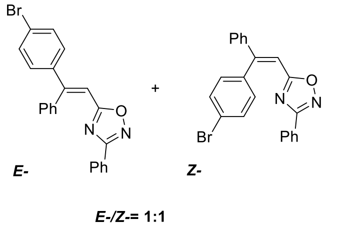

(*E*-/*Z*-)-5-(2-(4-Bromophenyl)-2-phenylethenyl)-3-phenyl-1,2,4-oxadiazole*(E*-/*Z*-5e). Yield of 61%, ratio of *E*-/*Z*-isomers 1:1. Beige solids, mp 155-157°C (for mixture of *E-/Z*-isomers). Selected signals for *E*-isomer: ^{1}HNMR (400 MHz, $CDCl_3$) δ 7.02 (s, 1H, =CH). ^{13}C NMR (100 MHz, $CDCl_3$) δ109.8 (=CH). Selected signals for *Z*-isomer: ^{1}HNMR (400 MHz, $CDCl_3$)δ7.04 (s, 1H, =CH).^{13}C NMR (100 MHz, $CDCl_3$) δ 109.9(=CH). Signals for *E-/Z*-isomers: ^{1}HNMR (400 MHz, $CDCl_3$)δ 7.18 (d, *J* = 7 Hz,2H), 7.27 (d, *J* = 7 Hz,4H), 7.38-7.39 (m, 3H), 7.43-7.47 (m, 10H), 7.51 (d, *J* = 7 Hz,3H), 7.58 (d, *J* = 7 Hz,2H), 7.96-7.98 (t, 4H).^{13}C NMR (100 MHz, $CDCl_3$) δ 123.3, 124.5, 126.9, 126.9, 127.4, 127.5, 127.6, 128.4, 128.5, 128.8, 128.9, 128.5, 129, 129.2, 129.4, 129.6, 130, 130.2, 131.2, 131.2, 131.3, 131.4, 131.7, 131.9, 132.4, 137.2, 137.7, 139.6, 140.1, 141.4, 154.6, 154.7, 168.3, 168.4, 174.4, 174.5. GC-MS, m/z, $I_{rel.}$, %: 402 M^+ (68); 404 (64); 403 (100); 401 (84); 271 (42); 203 (72); 178 (89); 165 (61). HRMS: found $C_{22}H_{16}BrN_2O$ 403.0441 $[M+H]^+$, calcd.403.0441.

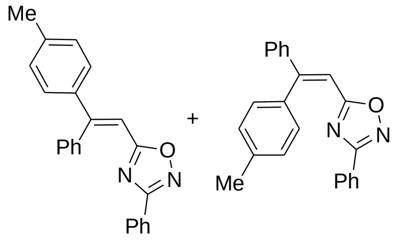

E-/Z- 1 : 1.4

(*E*-/*Z*-)-3-Phenyl-5-(2-phenyl-2-(4-methylphenyl)ethenyl)-1,2,4-oxadiazole (*E*-/*Z*-5f). Yield of 63%, ratio of *E*-/*Z*-isomers 1:1.4. Beige solids, mp 103-105°C (for mixture of *E*-/*Z*-isomers). Selected signals for *E*-isomer: ^{1}HNMR (400 MHz, $CDCl_3$) δ2.44 (s, 3H, CH_3), 6.99 (s, 1H, =CH).^{13}C NMR (100 MHz, $CDCl_3$) δ 21.60 (CH_3), 109.3 (=CH). Selected signals for *Z*-isomer: ^{1}HNMR (400 MHz, $CDCl_3$) δ 2.39 (s, 3H, CH_3), 7.03 (s, 1H, =CH).^{13}C NMR (100 MHz, $CDCl_3$) δ21.45 (CH_3), 108.6 (=CH). Signals for *E-/Z*-isomers: ^{1}HNMR (400 MHz, $CDCl_3$) δ7.18 (d, *J*=8 Hz, 3H), 7.24 (s, 1H), 7.27-7.32 (m, 5H), 7.40-7.46 (m, 11H), 7.95-8.01 (m, 4H).^{13}CNMR (100 MHz, $CDCl_3$) δ 127.4, 127.5, 128.4, 128.5, 128.6, 128.7, 128.8, 128.9, 129.1, 129.4, 129.6, 129.7, 129.9, 131.1, 131.2, 135.4, 137.8, 138.5, 139.0, 140.4, 141.1, 155.9, 156.2, 168.2, 174.9. HRMS: found $C_{23}H_{19}N_2O$ 339.1497 $[M+H]^+$, calcd. 339.1494.

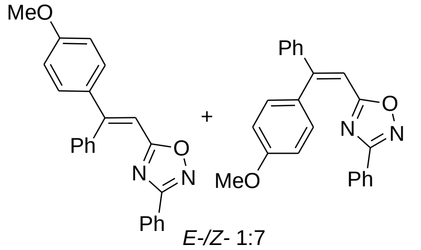

E-/Z- 1:7

(*E-/Z*-)-5-(2-(4-Methoxyphenyl)-2-phenylethenyl)-3-phenyl-1,2,4-oxadiazole (*E*-/*Z*-5g). Yield of 70%, ratio of *E*-/*Z*-isomers 1:7. Beige solids, mp 94-96°C (for mixture of *E-/Z*-isomers). Selected signals for *E*-isomer: ^{1}HNMR (400 MHz, $CDCl_3$)δ 3.84 (s, 3H, OMe), 6.90 (d, *J*=8.8Hz, 2H), 6.99 (s, 1H, =CH), 7.36 (d, *J*=8.8Hz,2H), 7.97(d, *J*=7 Hz,2H).^{13}CNMR (100 MHz, $CDCl_3$) δ 55.5 (OMe); Selected signals for *Z*-isomer: ^{1}HNMR (400 MHz, $CDCl_3$)δ 3.75 (s, 3H, CH_3), 6.82 (d, *J*=8.6 Hz,2H), 7.20 (d, *J*=8.6 Hz,2H), 8.02 (d, *J*=8 Hz,2H).^{13}CNMR (100 MHz, $CDCl_3$) δ 55.3 (OMe). Signals for *E-/Z*-isomers: ^{1}HNMR (400 MHz, $CDCl_3$) δ 7.28-7.30 (m, 4H), 7.43-7.48 (m, 4H).^{13}CNMR (100 MHz, $CDCl_3$) δ107.5, 114.1, 114.2, 126.9, 127.0, 127.4, 127.5, 127.6, 128.4, 128.7, 128.8, 128.9, 129.6, 130.0, 131.0, 131.2, 131.5, 134.7, 138.5, 143.0, 155.4, 158.5, 161.2, 168.1, 168.3, 175.0, 178.3. HRMS: found $C_{23}H_{19}N_2O_2$ 355.1447$[M+H]^+$, calcd. 355.1444.

3. ^{1}H and ^{13}C NMR spectra of compounds 2, 3, 4, 5

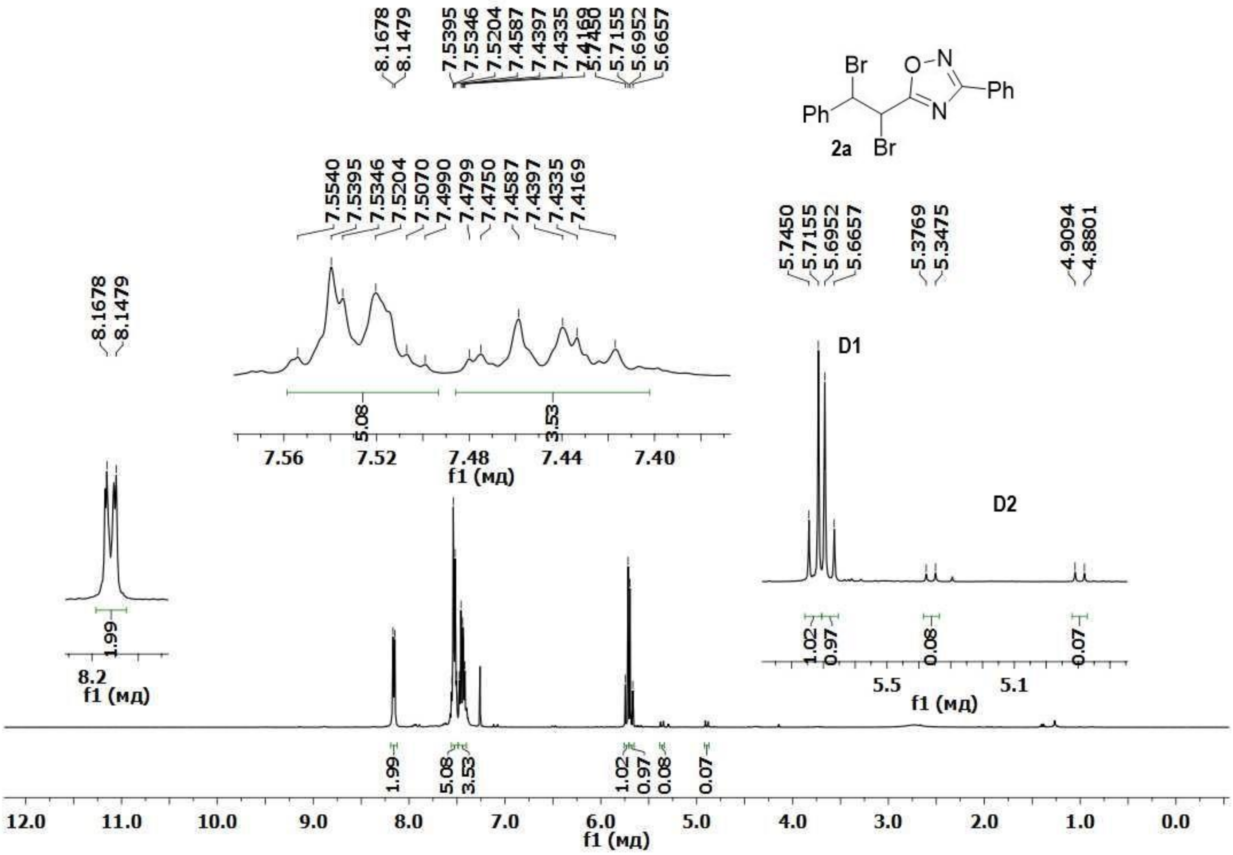

Figure S1. ^{1}H NMR spectrum of mixture of diestereomers of compound **2a** (400 MHz, $CDCl_3$)

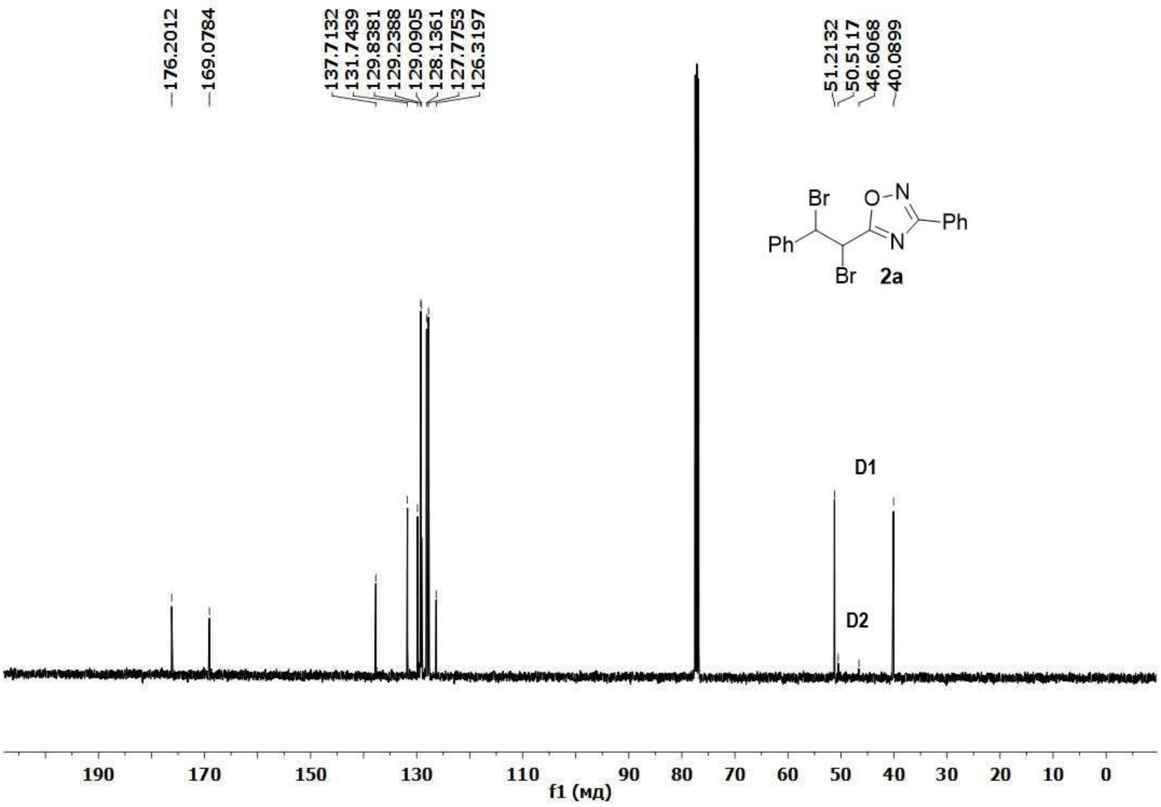

Figure S2. ^{13}C NMR spectrum of mixture of diestereomers of compound **2a** (100 MHz, $CDCl_3$).

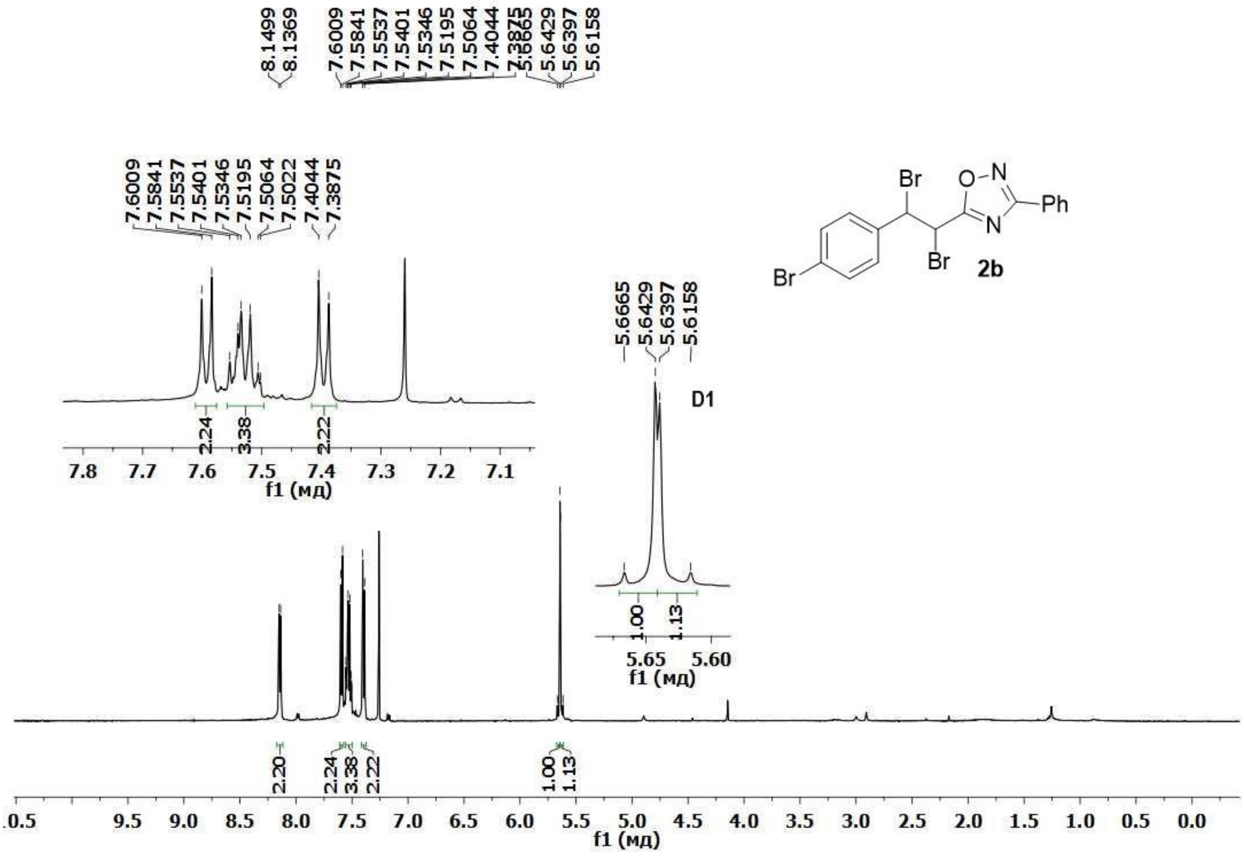

Figure S3. ^{1}H NMR spectrum of compound **2b** (500 MHz, $CDCl_3$).

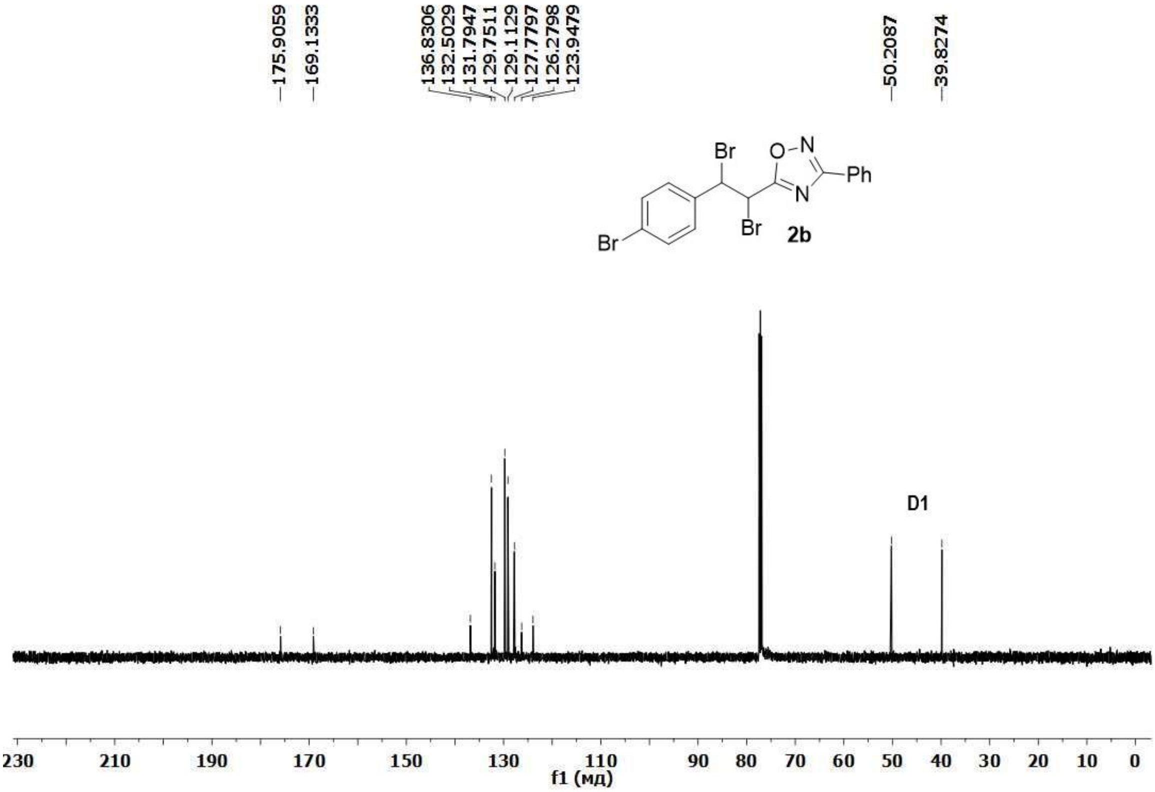

Figure S4. ^{13}C NMR spectrum of compound **2b** (125 MHz, $CDCl_3$).

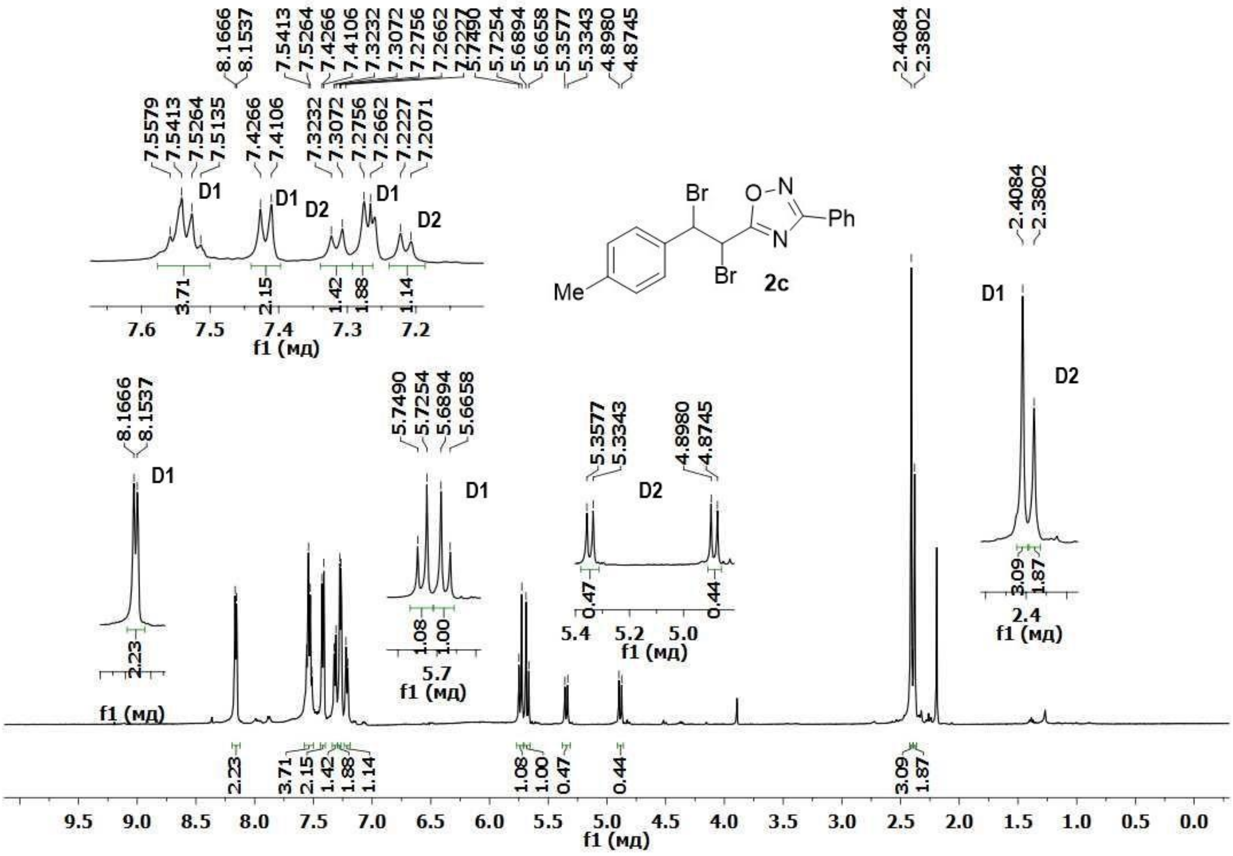

Figure S5. ^{1}H NMR spectrum of mixture of diestereomers of compound **2c** (500 MHz, $CDCl_3$)

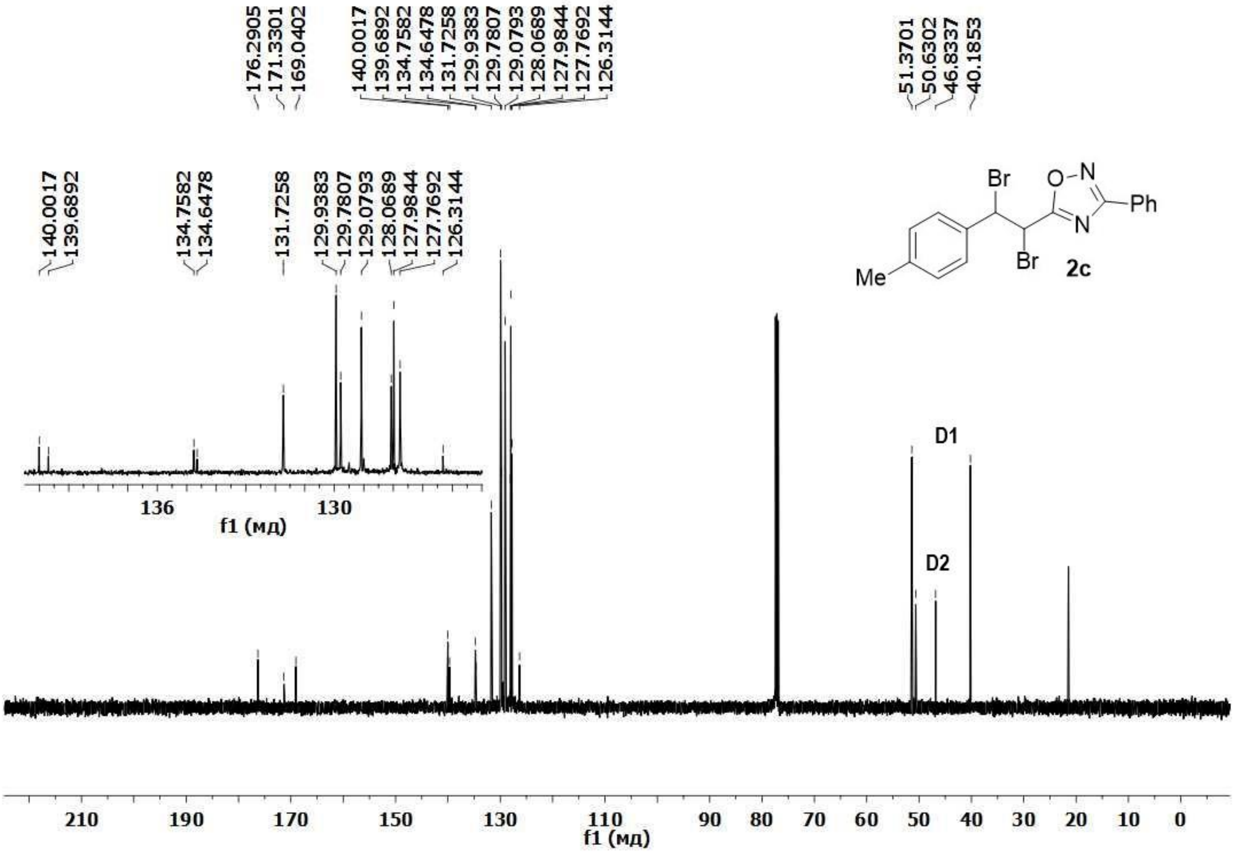

Figure S6. ^{13}C NMR spectrum of mixture of diestereomers of compound **2c** (125 MHz, $CDCl_3$).

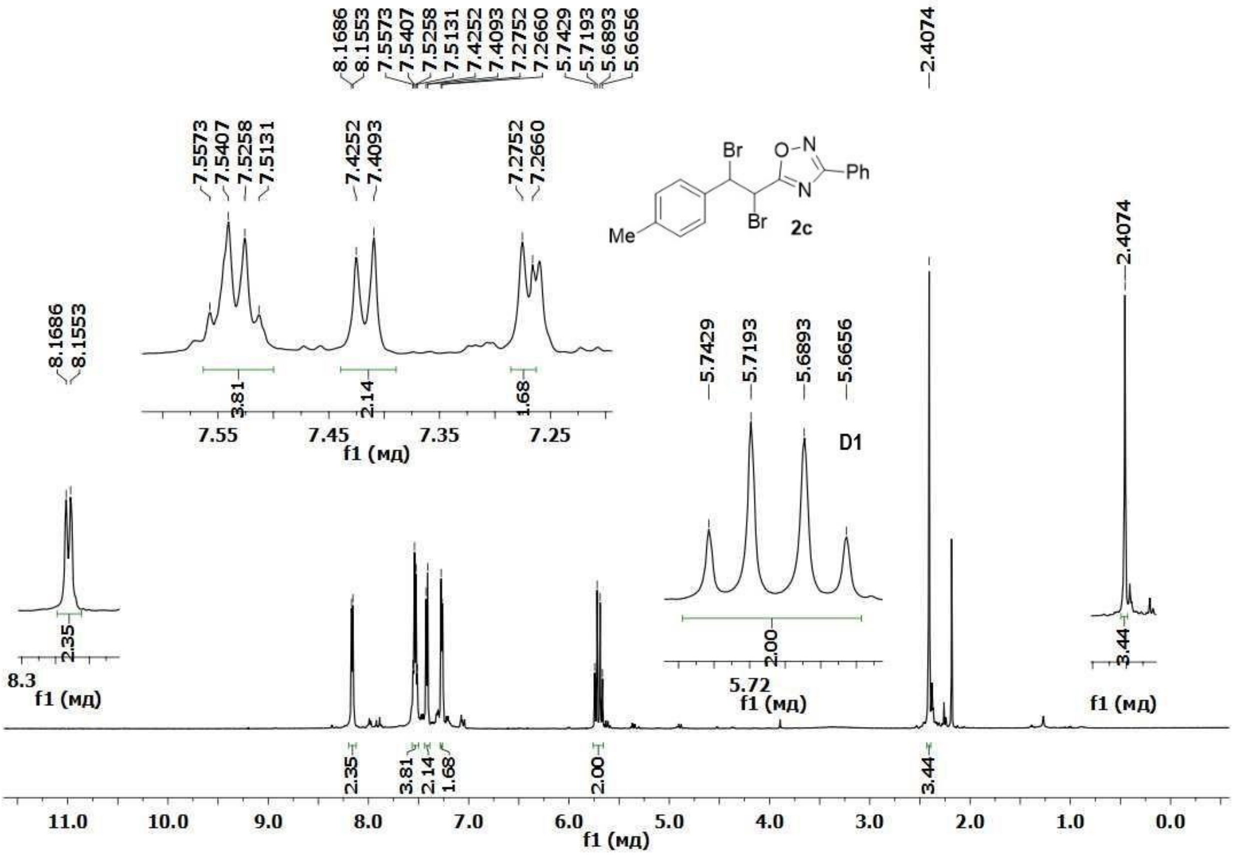

Figure S7. ^{1}H NMR spectrum of diestereomer 1 of compound **2c** (500 MHz, $CDCl_3$)

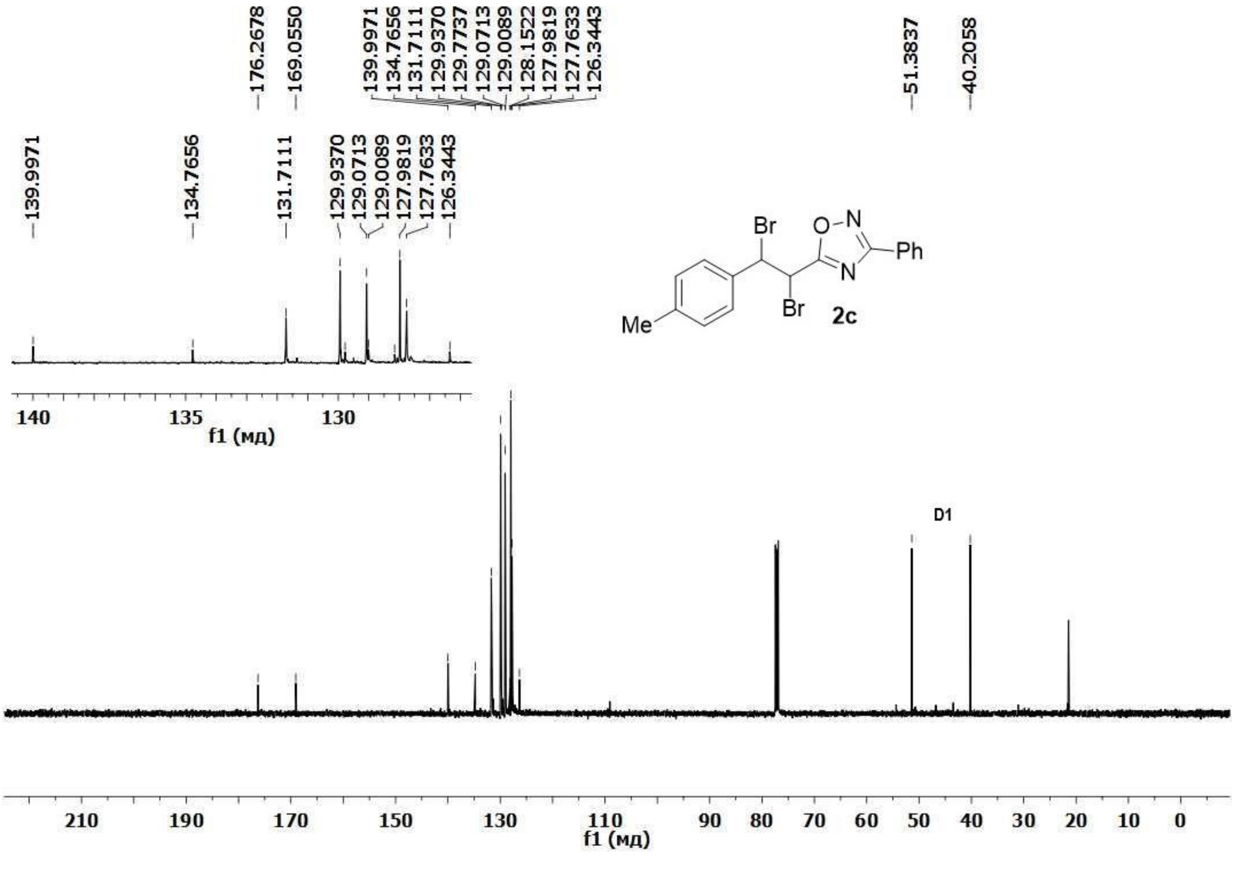

Figure S8. ^{13}C NMR spectrum of diestereomer 1 of compound **2c** (125 MHz, $CDCl_3$).

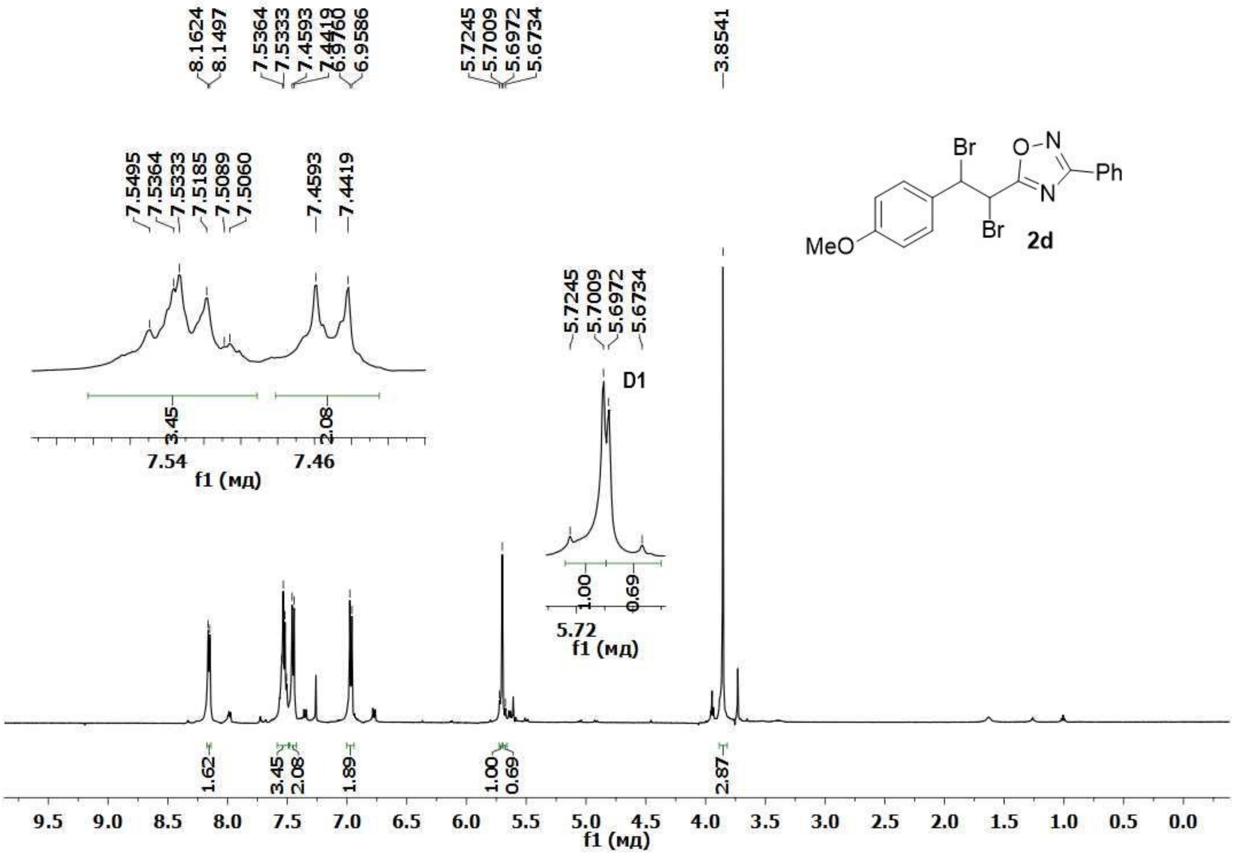

Figure S9. ^{1}H NMR spectrum of compound **2d** (500 MHz, $CDCl_3$)

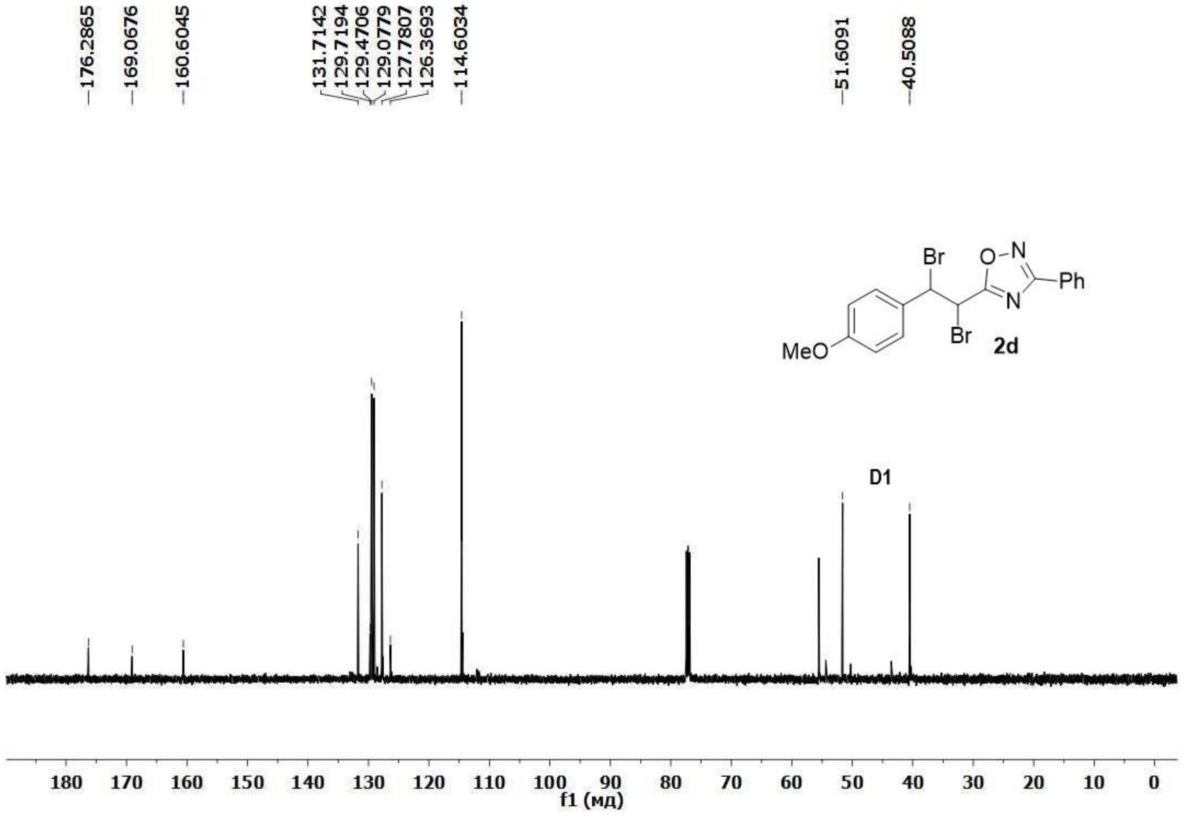

Figure S10. ^{13}C NMR spectrum of compound **2d** (125 MHz, $CDCl_3$).

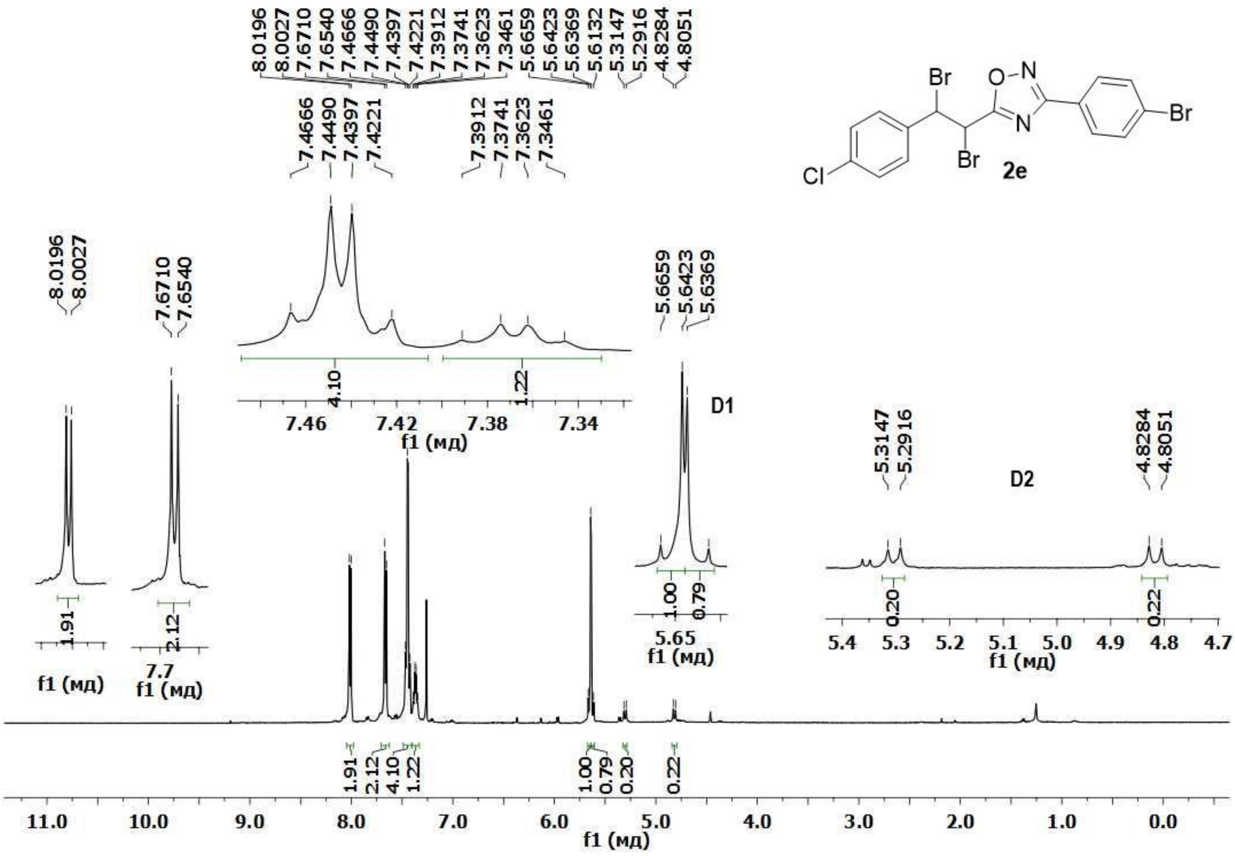

Figure S11. ^{1}H NMR spectrum of mixture of diestereomers of compound **2e** (500 MHz, $CDCl_3$)

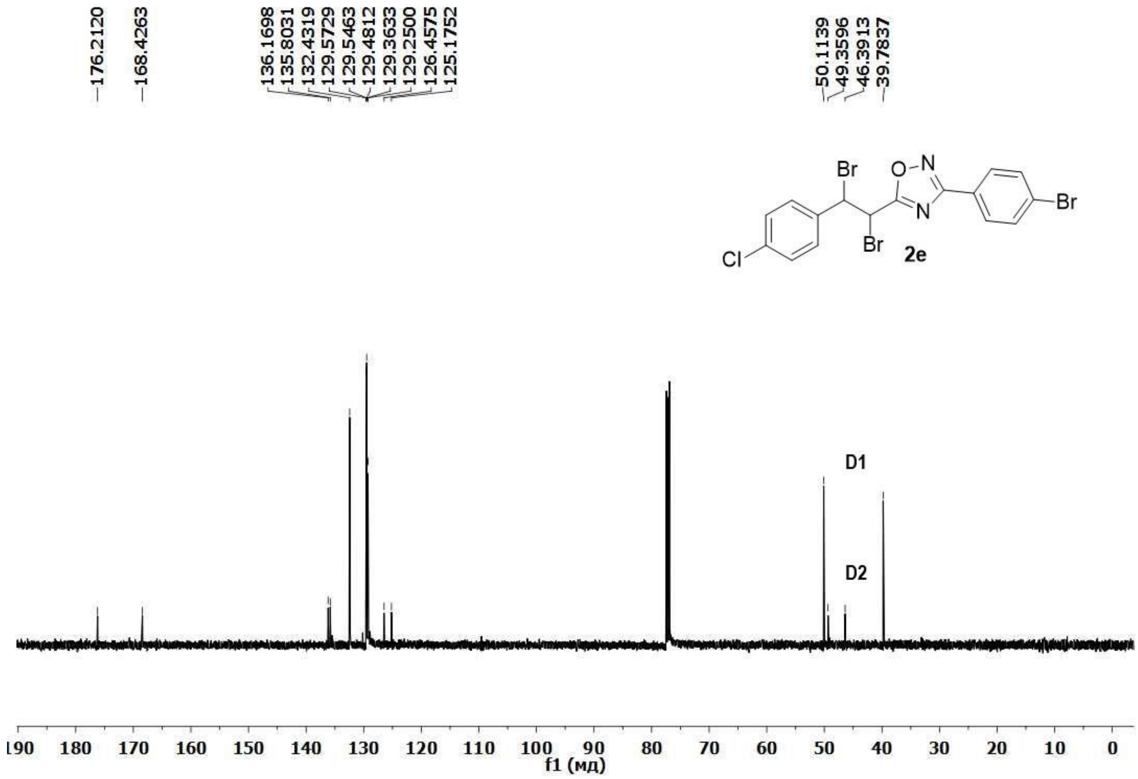

Figure S12. ^{13}C NMR spectrum of mixture of diestereomers of compound **2e** (125 MHz, $CDCl_3$).

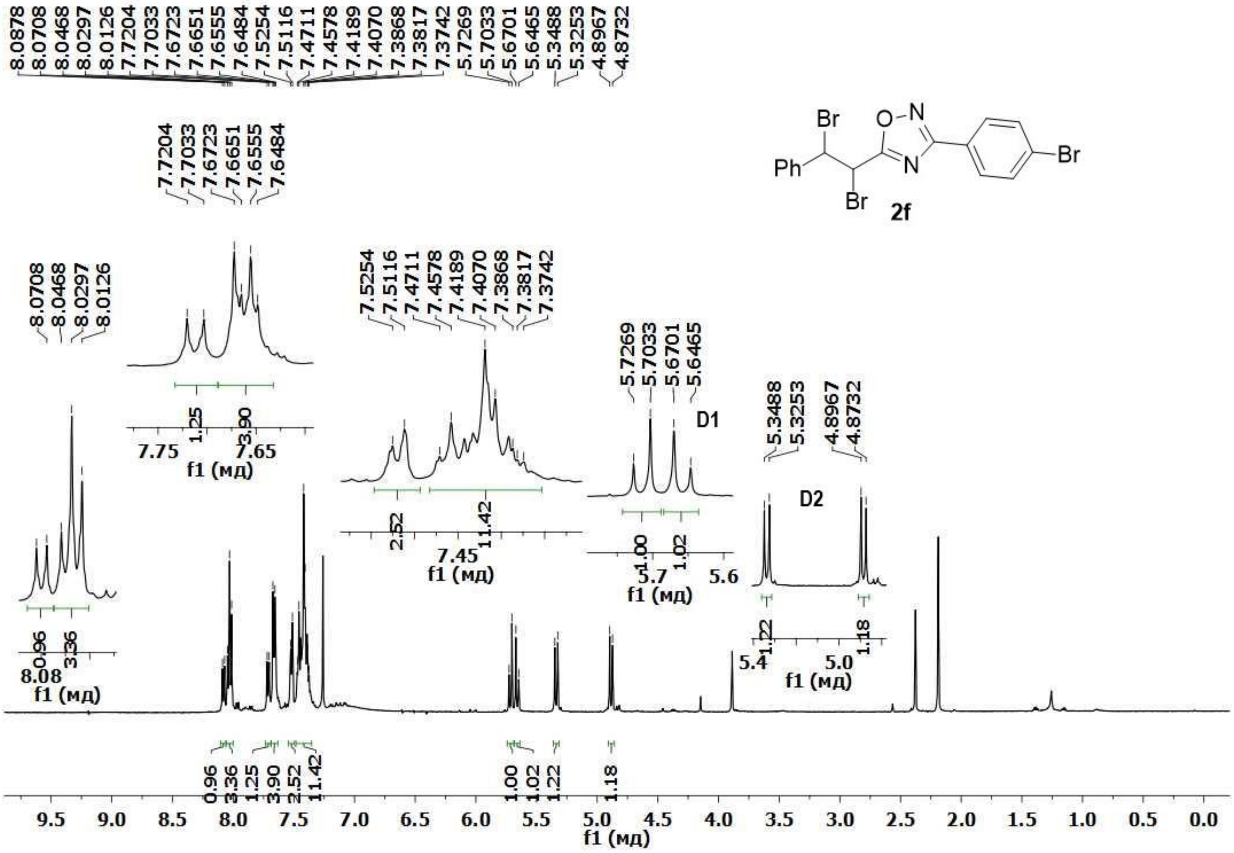

Figure S13. ^{1}H NMR spectrum of mixture of diestereomers of compound **2f** (500 MHz, $CDCl_3$)

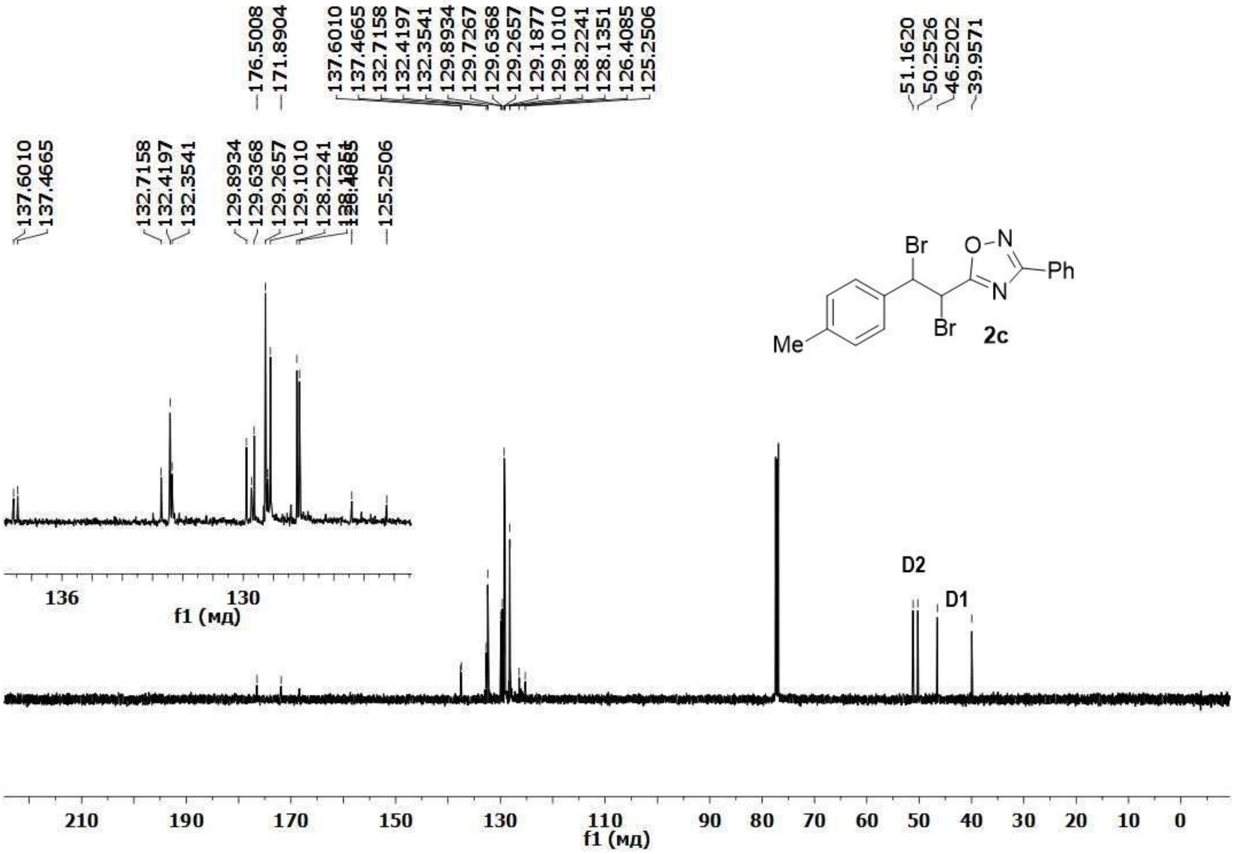

Figure S14. ^{13}C NMR spectrum of mixture of diestereomers of compound **2f** (125 MHz, $CDCl_3$).

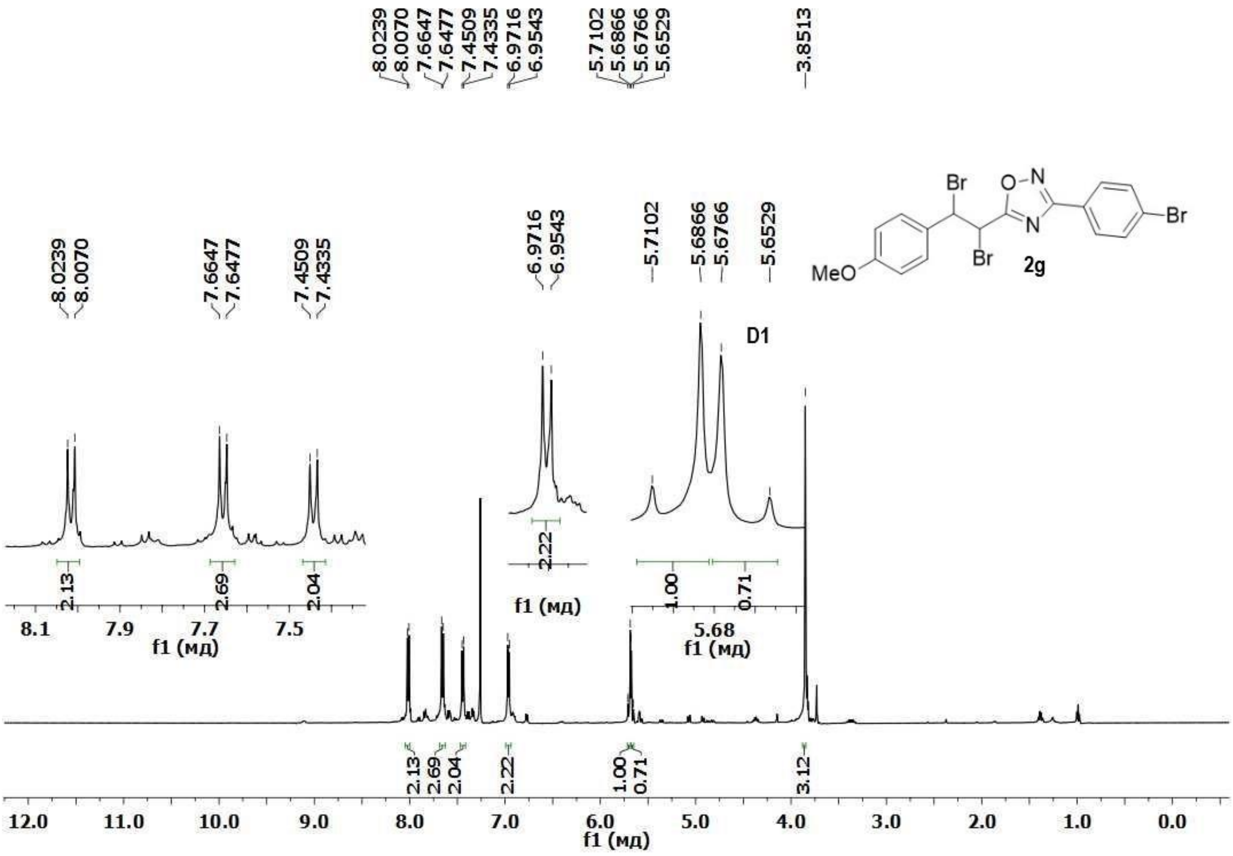

Figure S15. ^{1}H NMR spectrum of compound **2g** (500 MHz, $CDCl_3$).

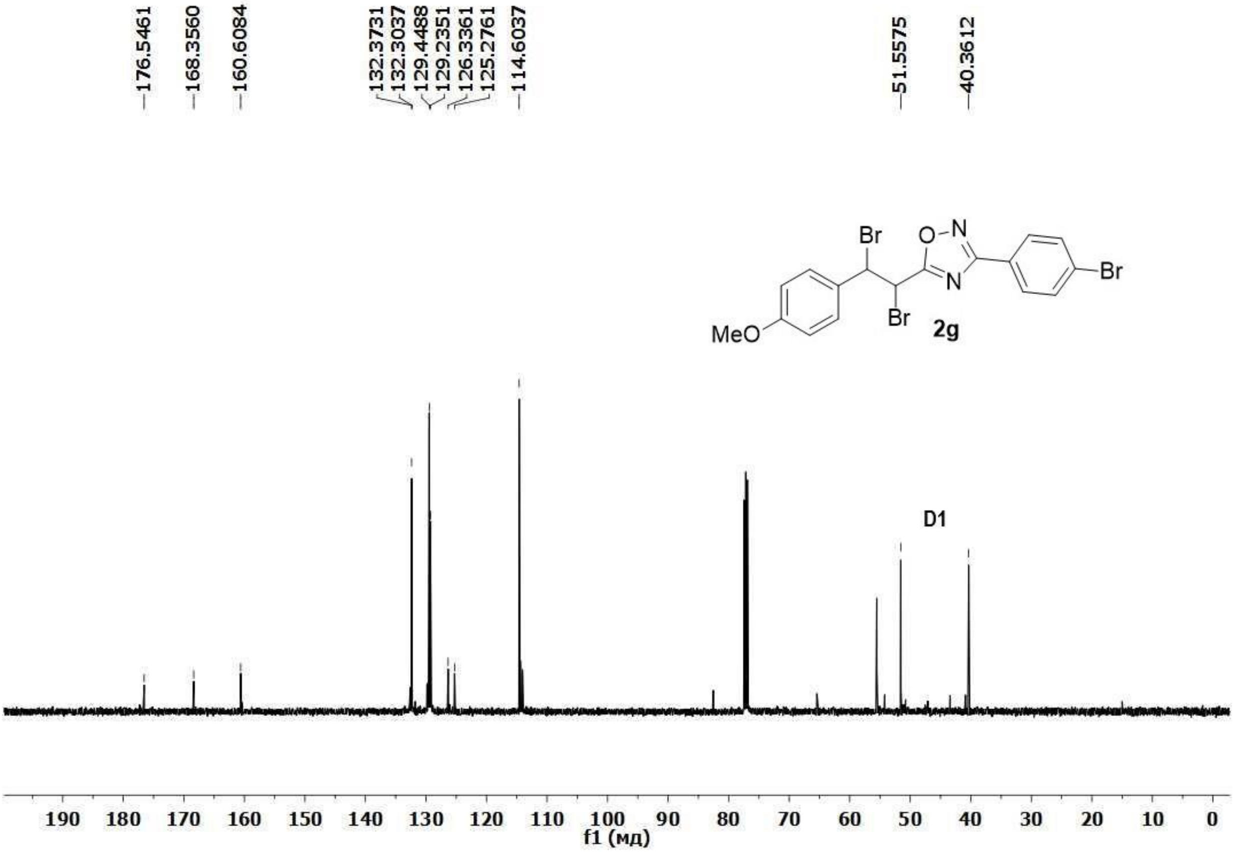

Figure S16. ^{13}C NMR spectrum of compound **2g** (125 MHz, $CDCl_3$).

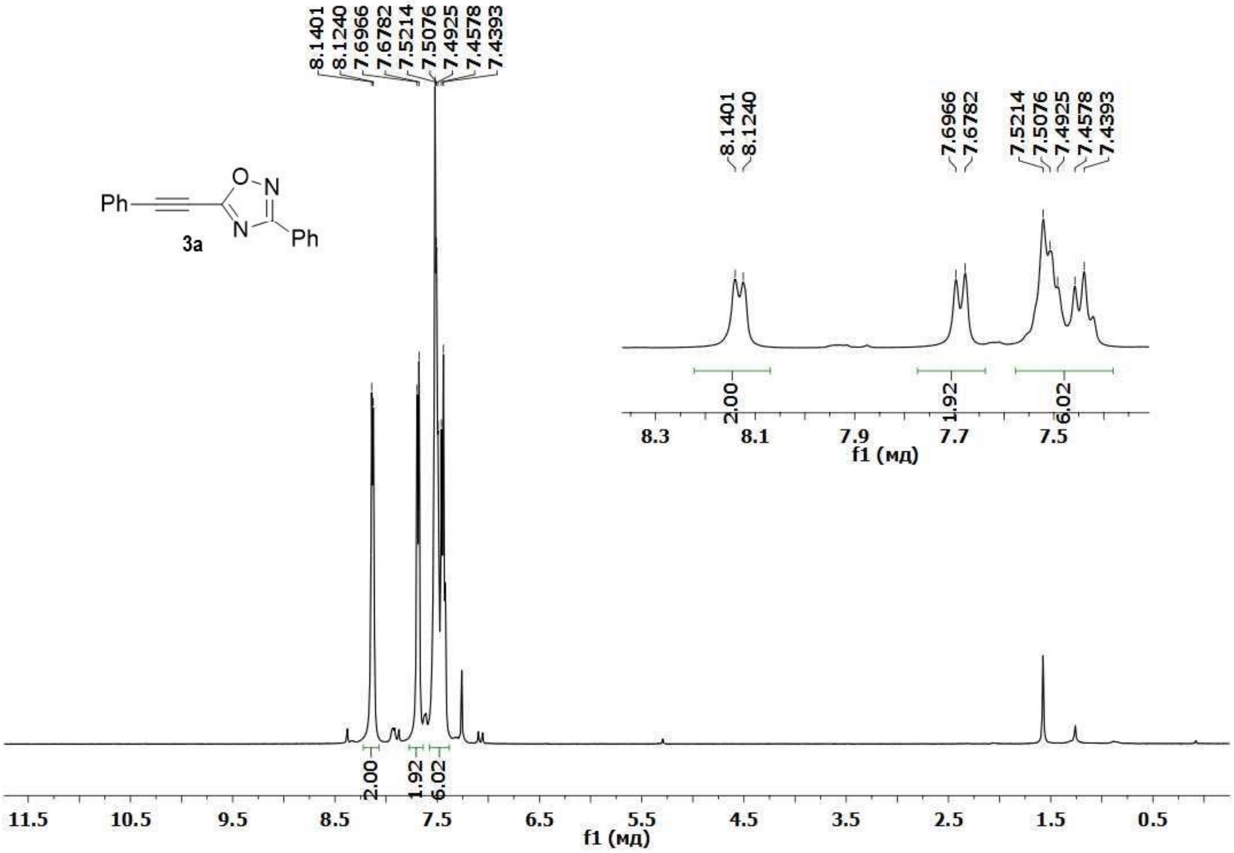

Figure S17. ^{1}H NMR spectrum of compound **3a** (400 MHz, $CDCl_3$).

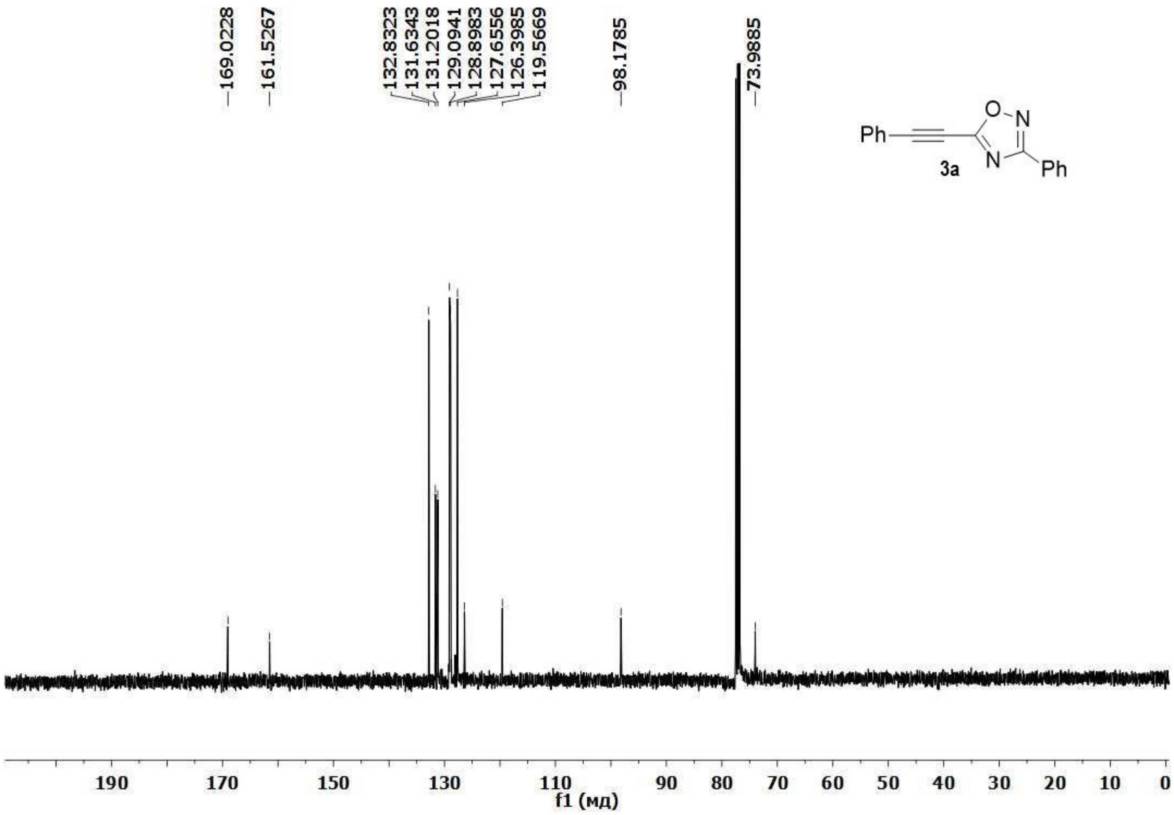

Figure S18. ^{13}C NMR spectrum of compound **3a** (100 MHz, $CDCl_3$).

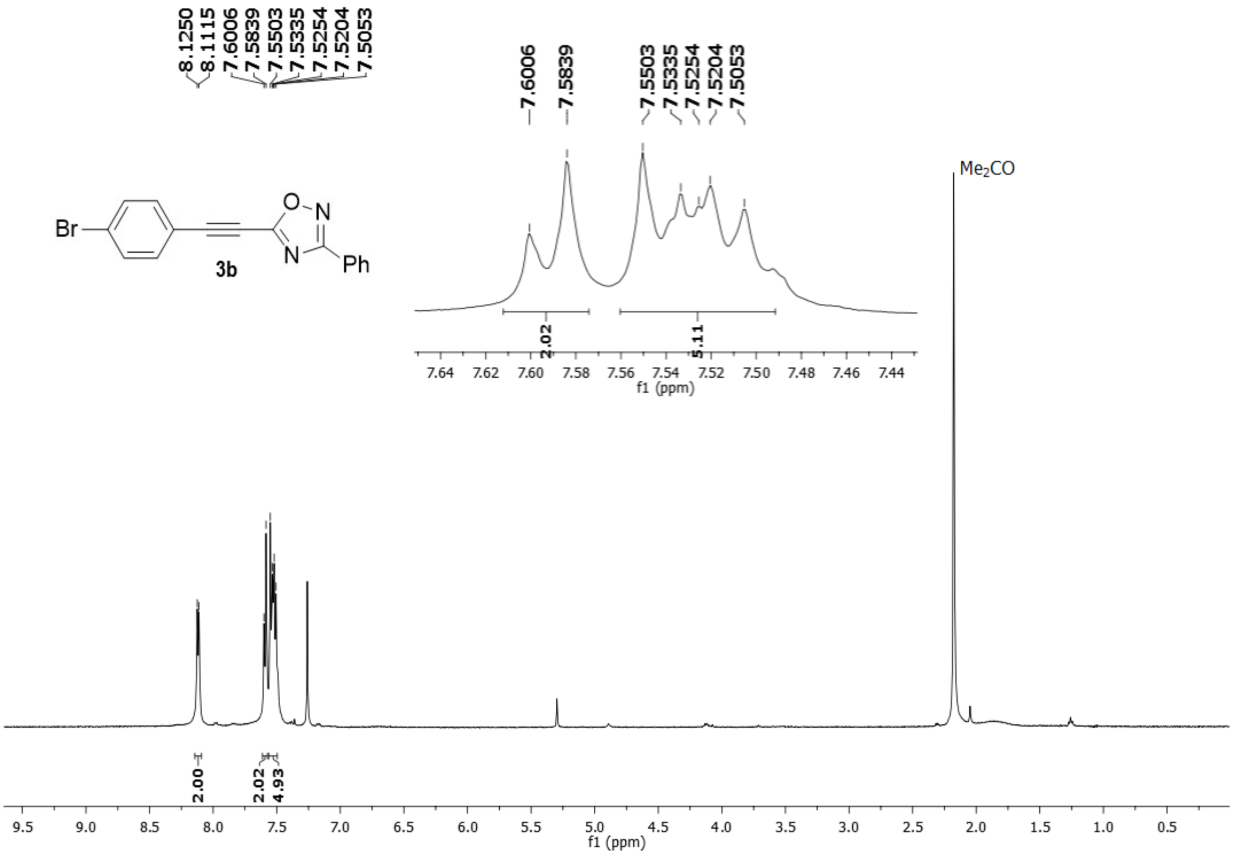

Figure S19. ^{1}H NMR spectrum of compound **3b** (500 MHz, $CDCl_3$).

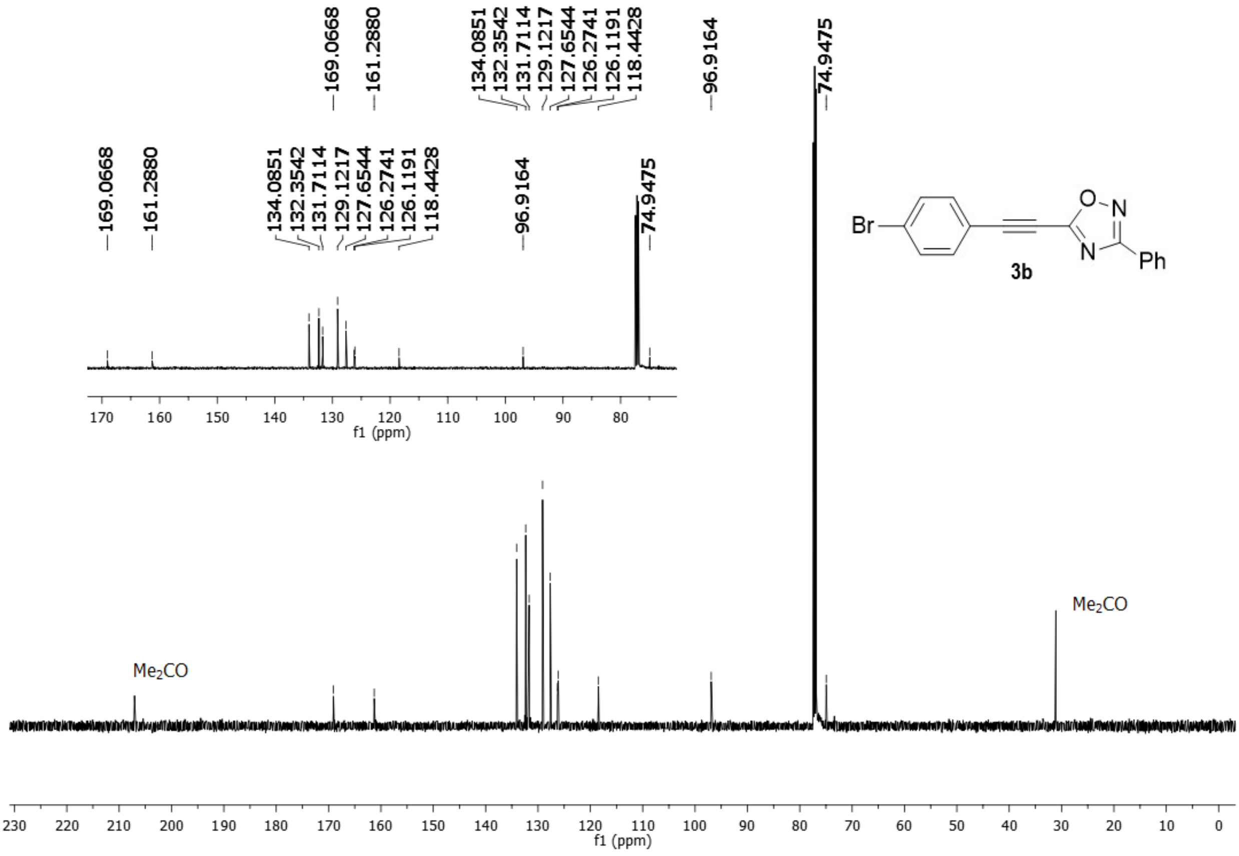

Figure S20. ^{13}C NMR spectrum of compound **3b** (125 MHz, $CDCl_3$).

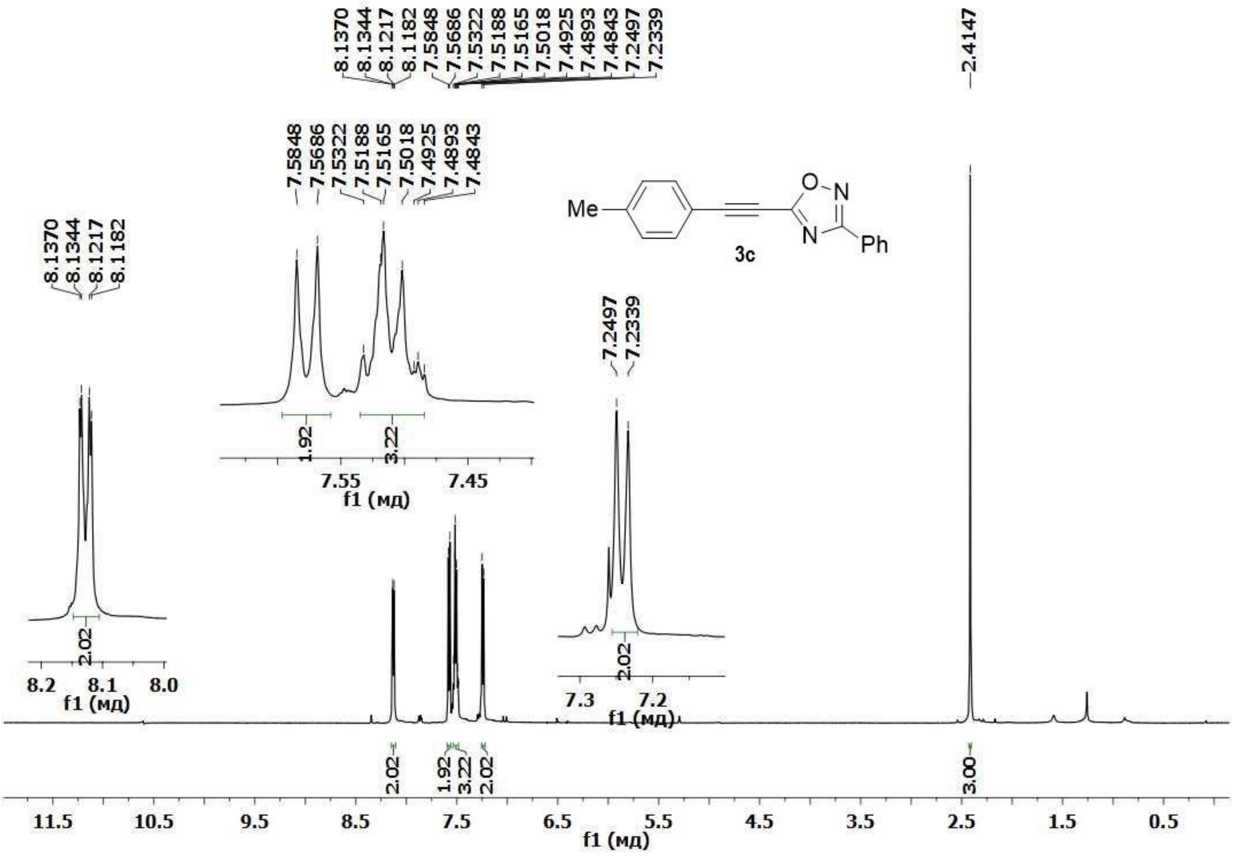

Figure S21. ^{1}H NMR spectrum of compound **3c** (400 MHz, $CDCl_3$).

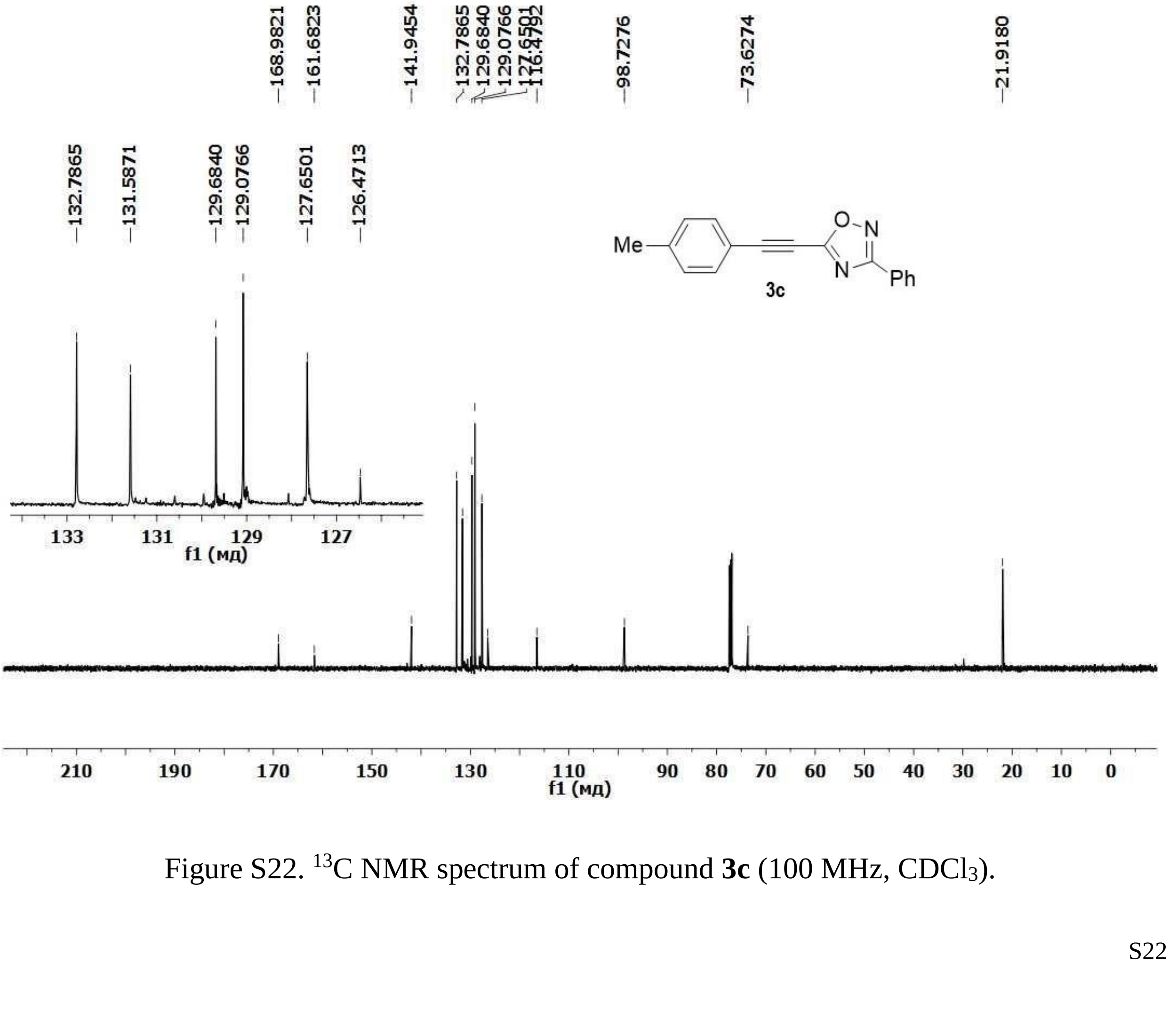

Figure S22. ^{13}C NMR spectrum of compound **3c** (100 MHz, $CDCl_3$).

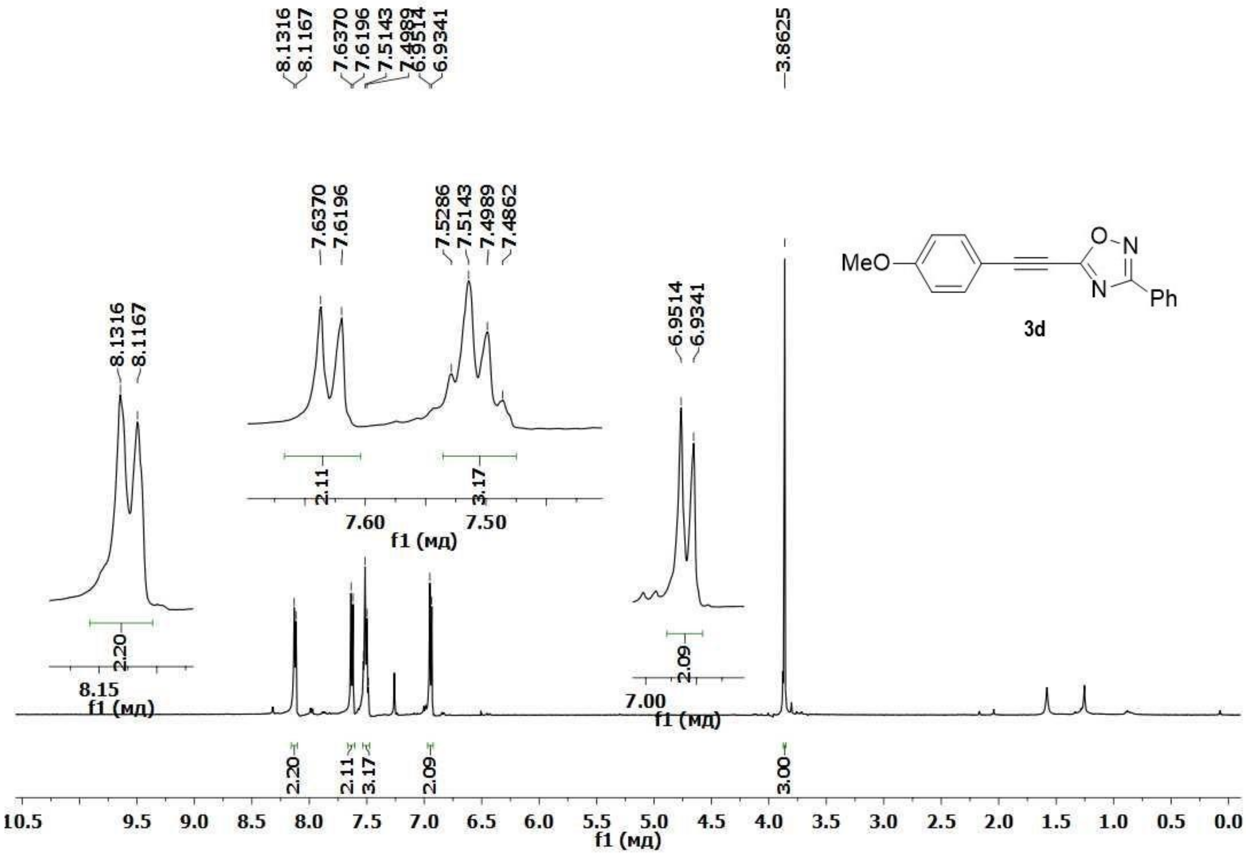

Figure S23. ^{1}H NMR spectrum of compound **3d** (400 MHz, $CDCl_3$).

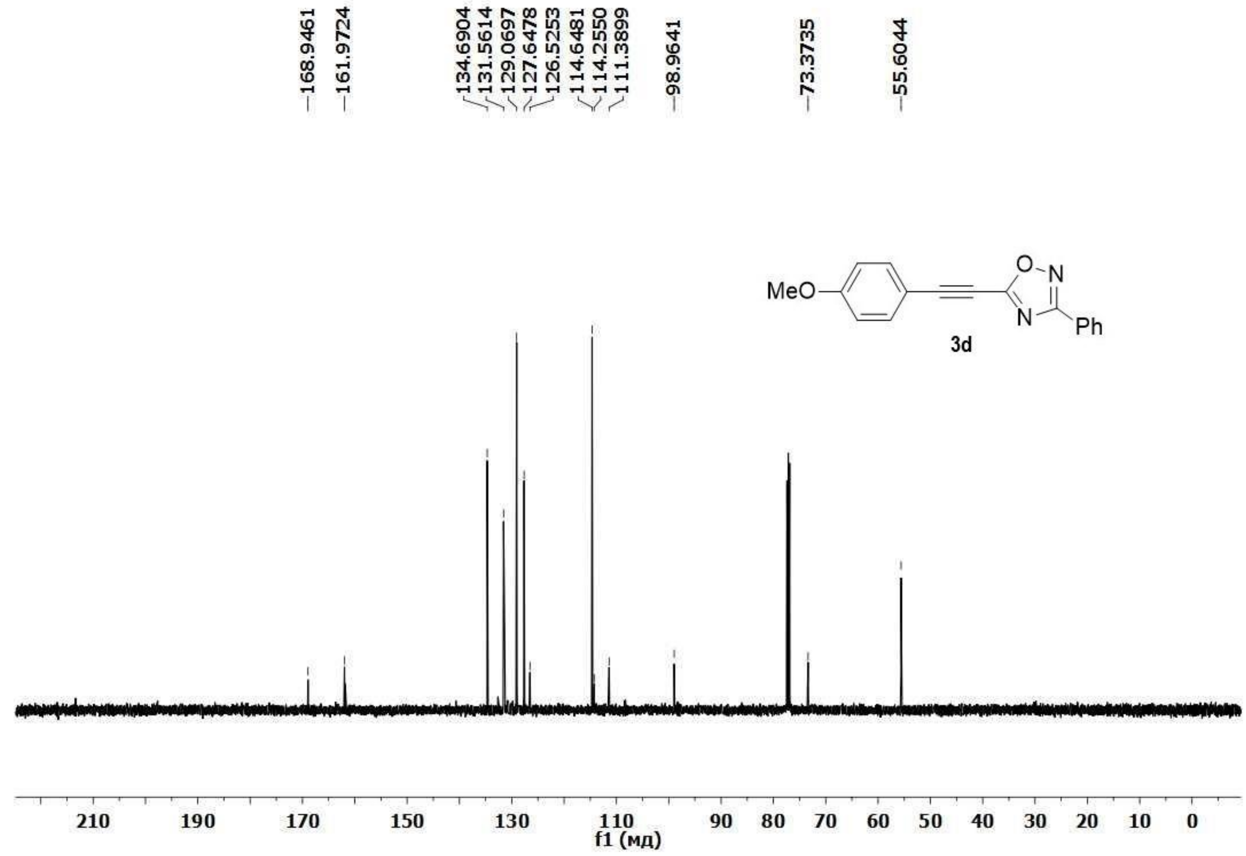

Figure S24. ^{13}C NMR spectrum of compound **3d** (100 MHz, $CDCl_3$).

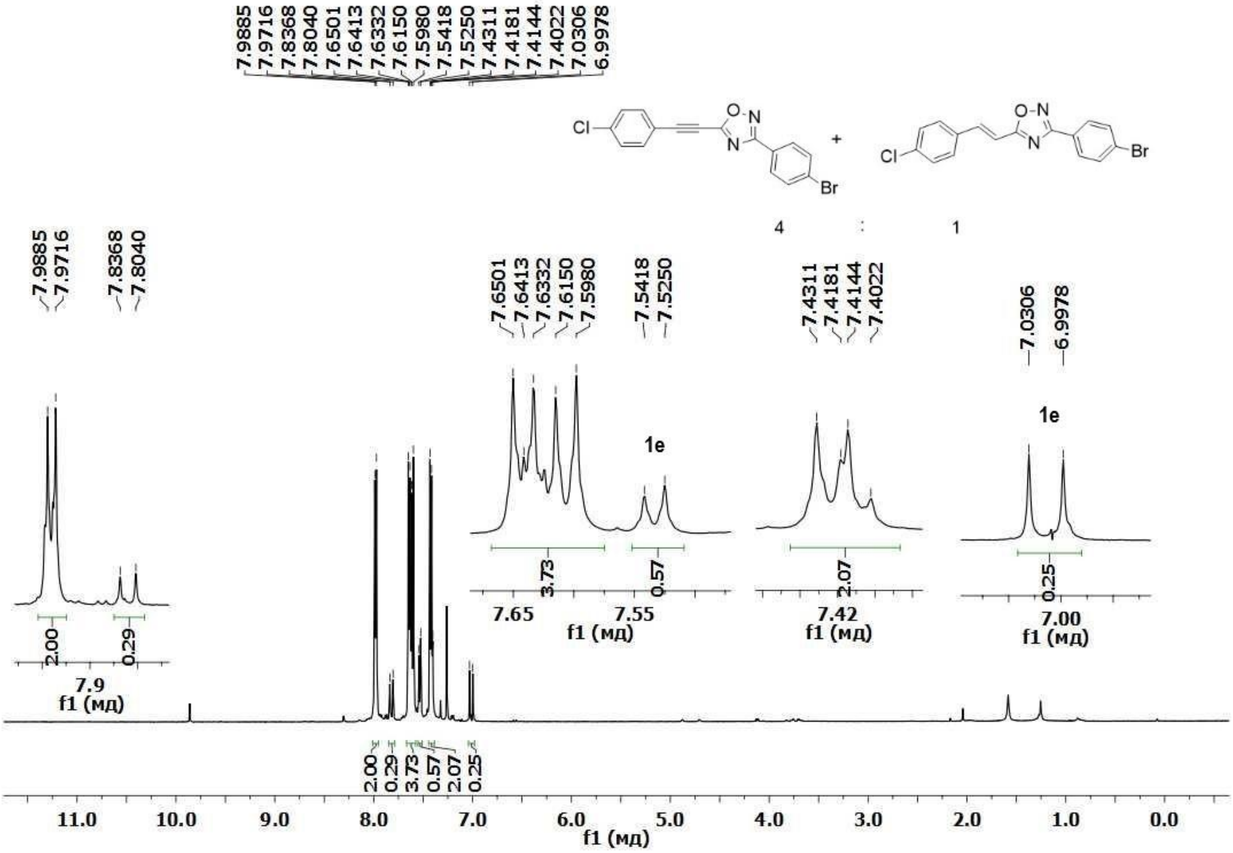

Figure S25. ^{1}H NMR spectrum of mixture compounds **3e** and **1e** (500 MHz, $CDCl_3$).

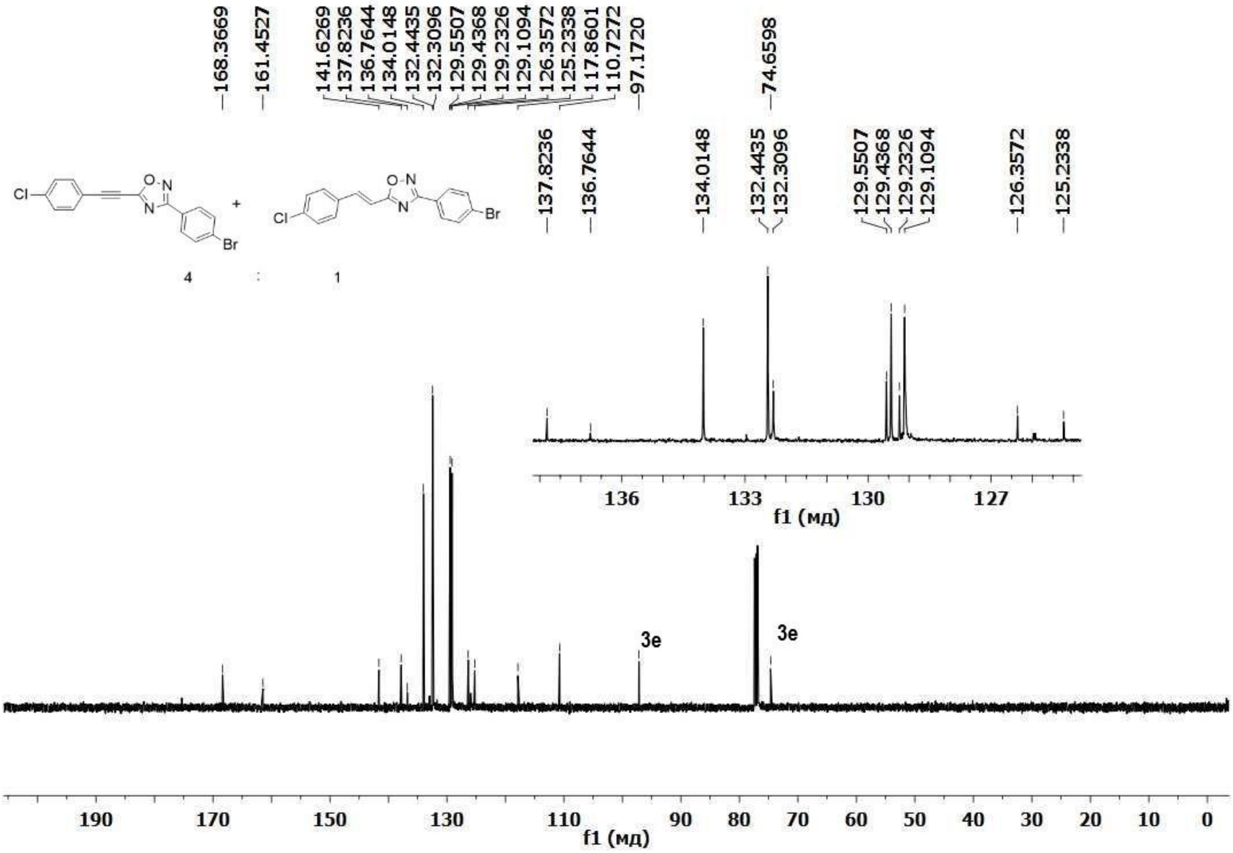

Figure S26. ^{13}C NMR spectrum of mixture compounds **3e** and **1e** (125 MHz, $CDCl_3$).

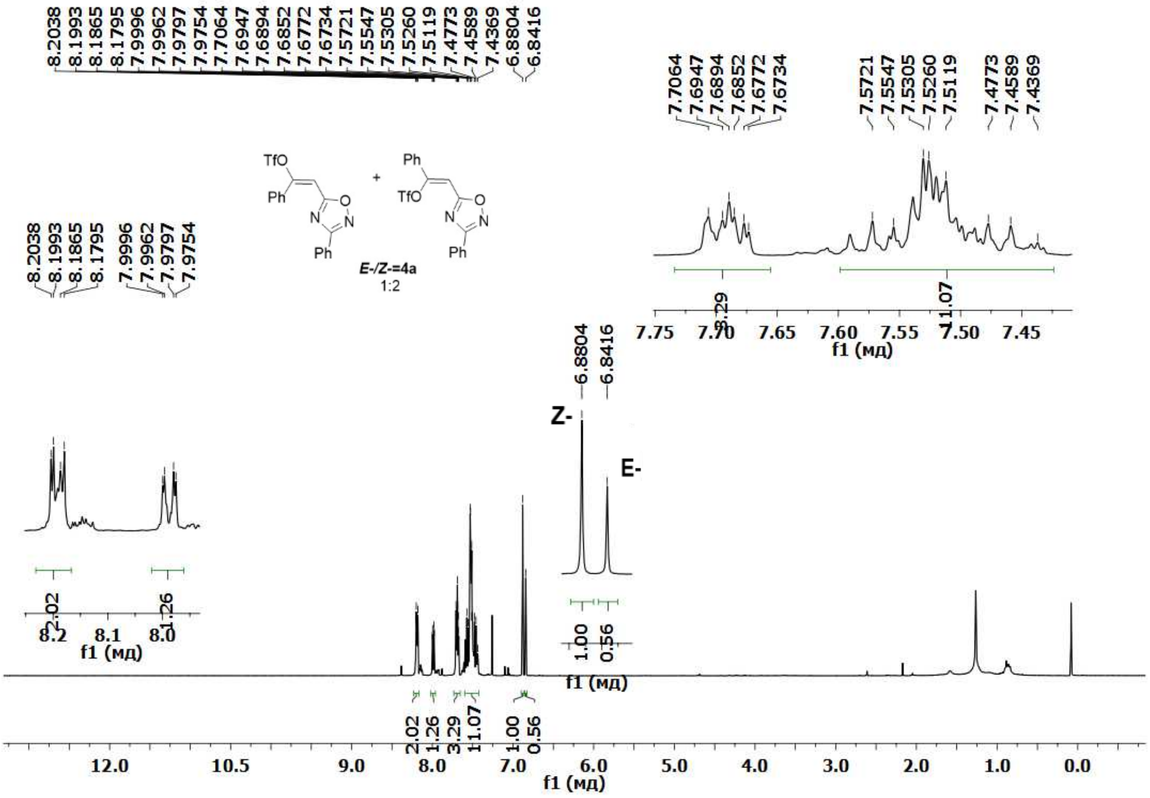

Figure S27. ^{1}H NMR spectrum of mixture of compounds ***E*-/*Z*-4a** (400 MHz, $CDCl_3$).

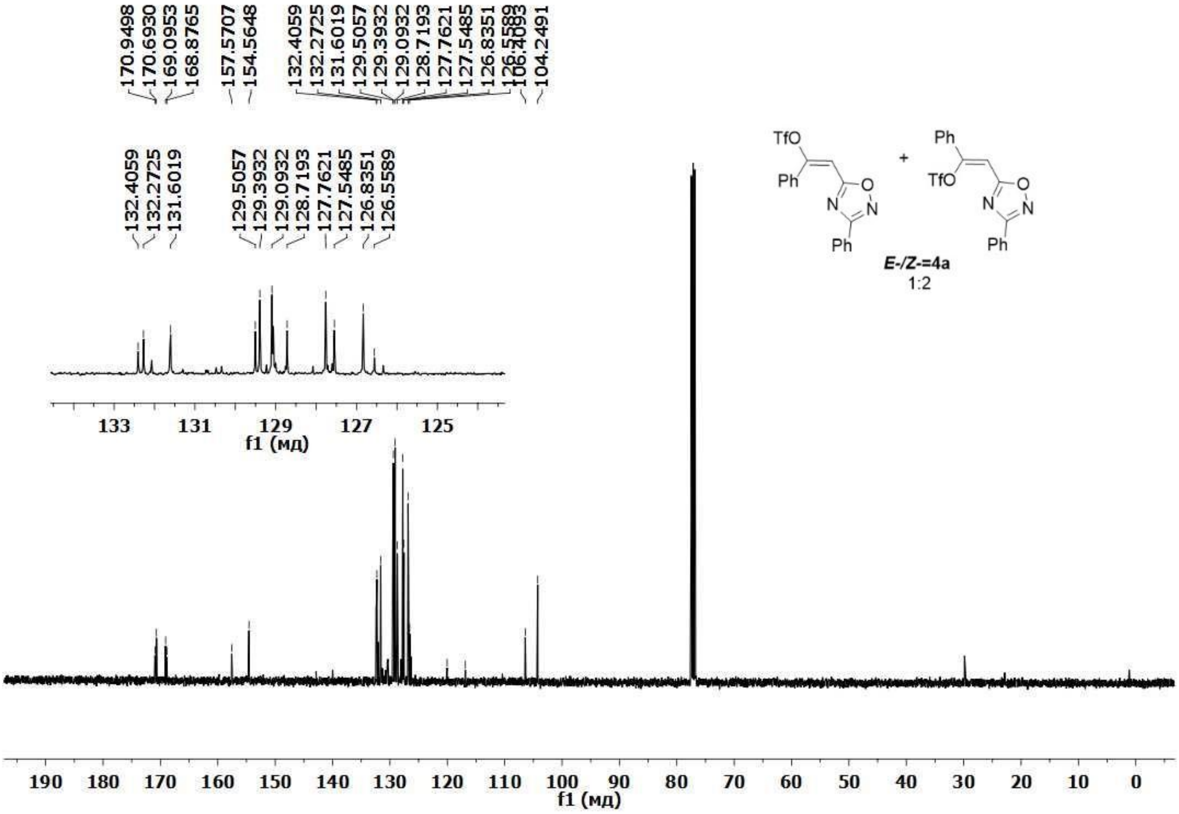

Figure S28. ^{13}C NMR spectrum of mixture of compounds ***E*-/*Z*-4a** (100 MHz, $CDCl_3$).

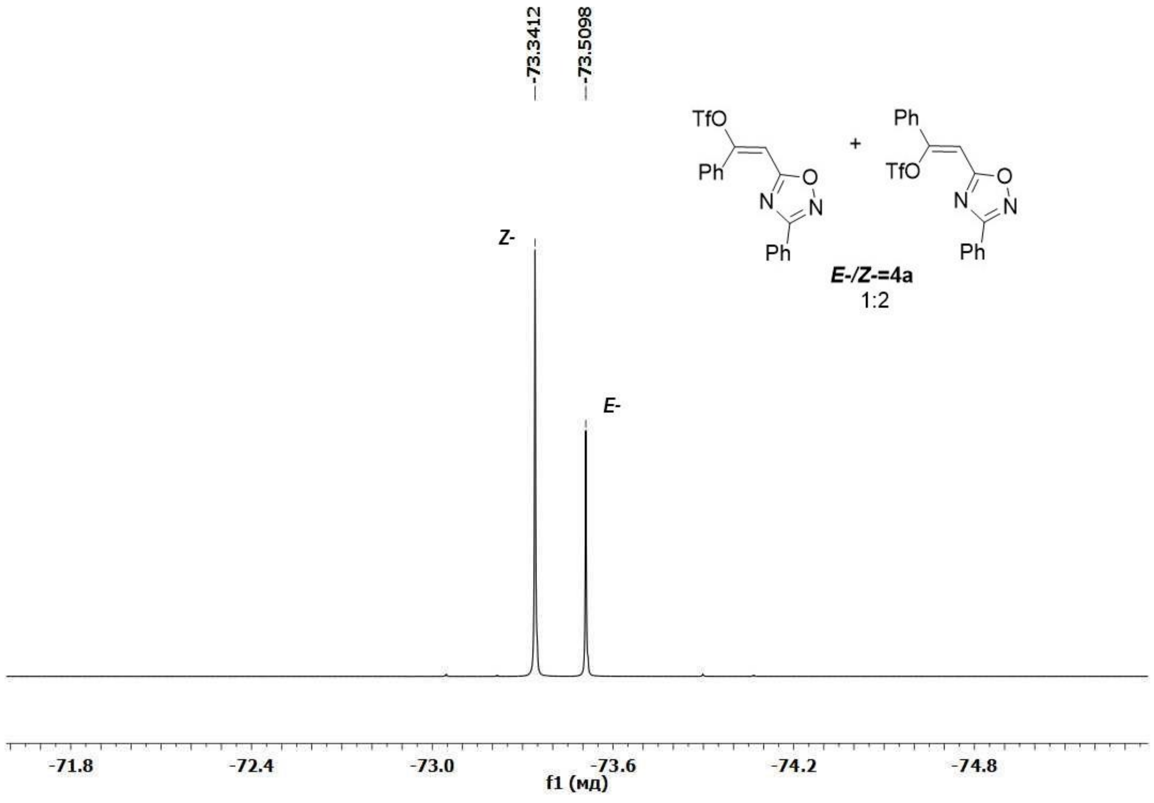

Figure S29. ^{19}F NMR spectrum of mixture of compounds ***E-/Z*-4a** (376 MHz, $CDCl_3$).

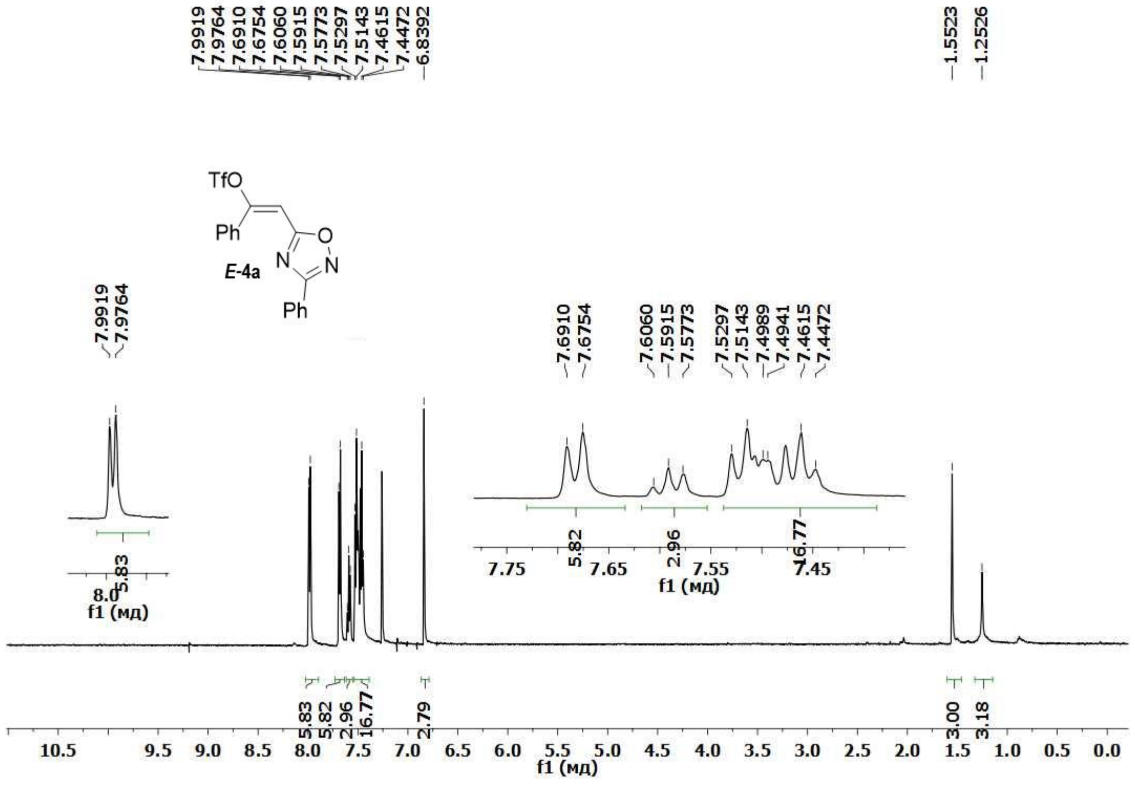

Figure S30. ^{1}H NMR spectrum of compound ***E*-4a** (500 MHz, $CDCl_3$).

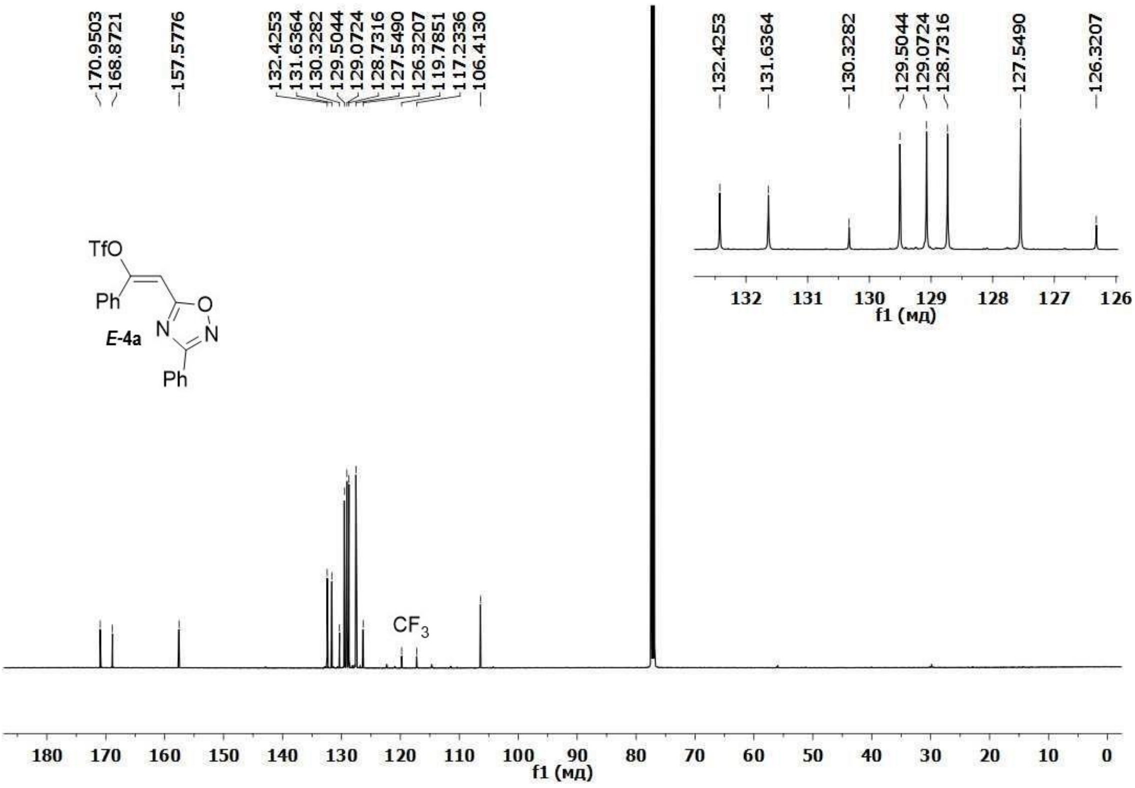

Figure S31. ^{13}C NMR spectrum of compound ***E*-4a** (125 MHz, $CDCl_3$).

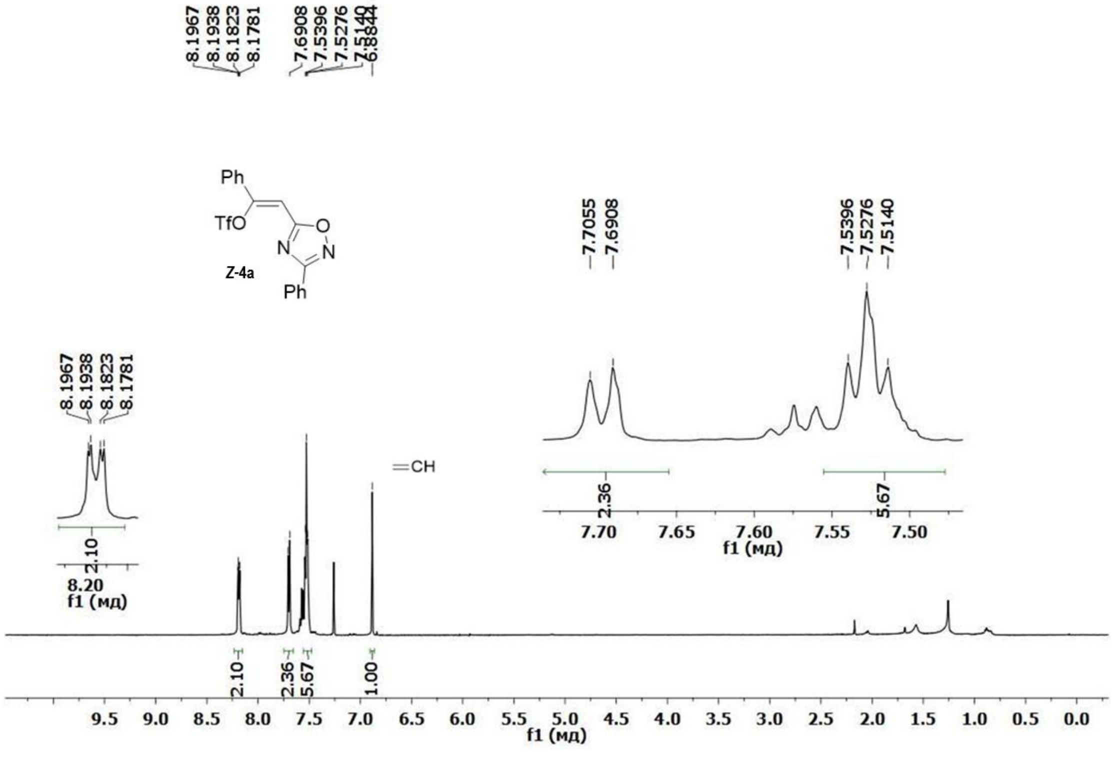

Figure S32. ^{1}H NMR spectrum of compound ***Z*-4a** (500 MHz, $CDCl_3$).

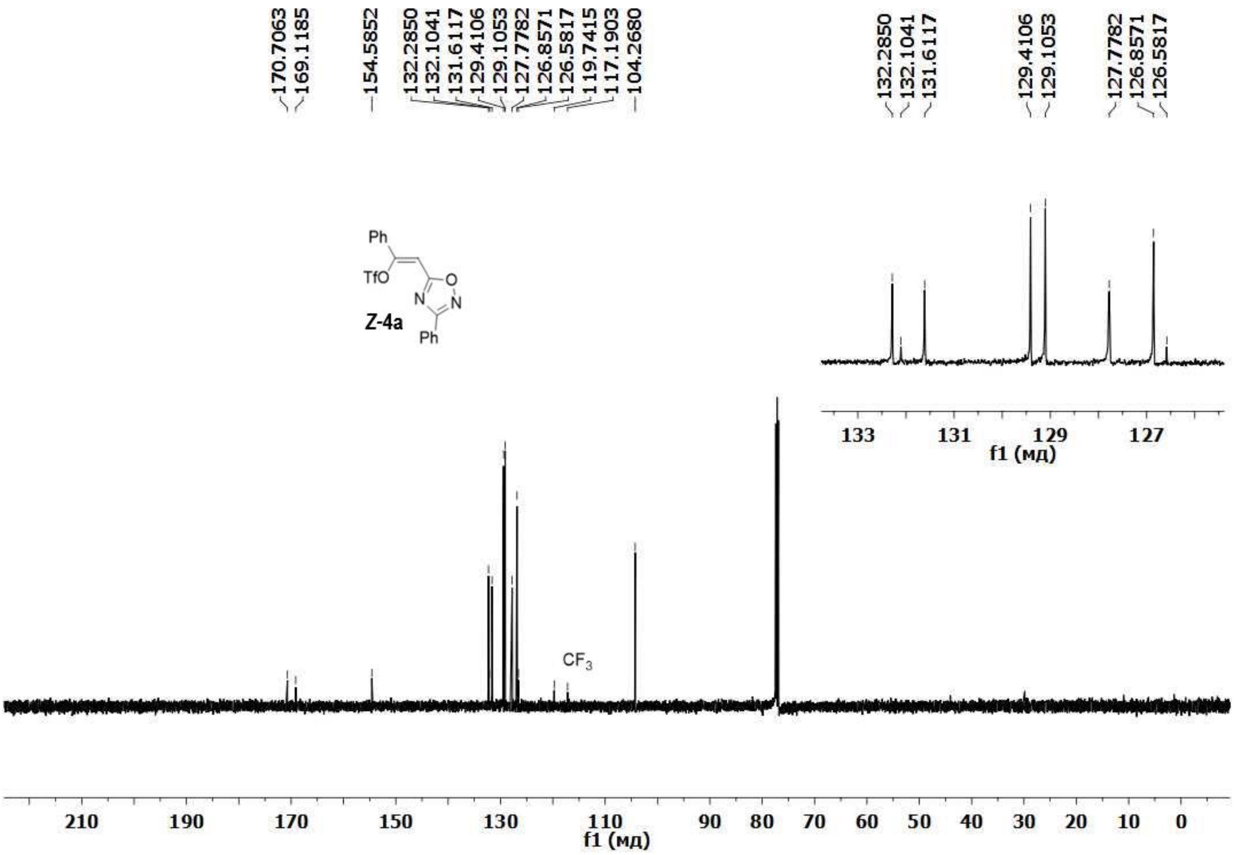

Figure S33. ^{13}C NMR spectrum of compound ***Z*-4a** (125 MHz, $CDCl_3$).

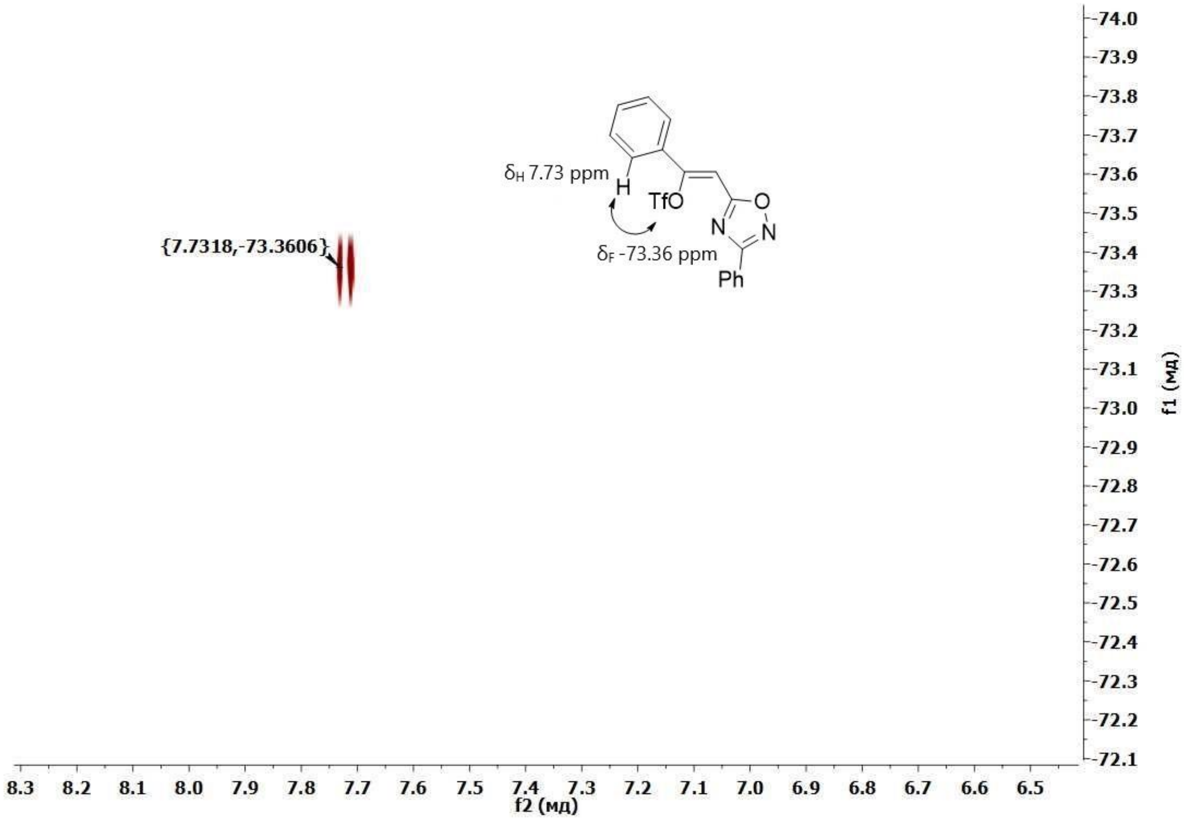

Figure S34. HOESY NMR spectrum of compounds ***Z*-4a** ($CDCl_3$).

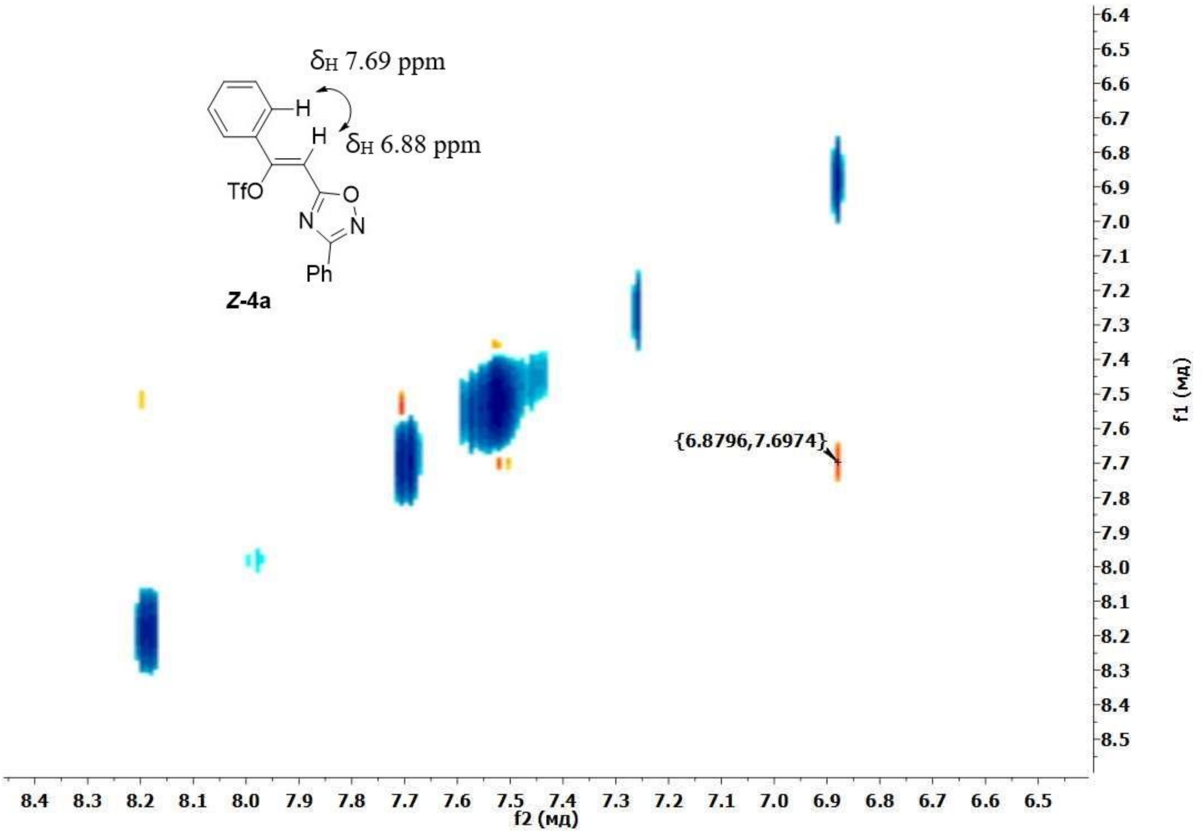

Figure S35. NOESY NMR spectrum of compounds ***Z*-4a** ($CDCl_3$).

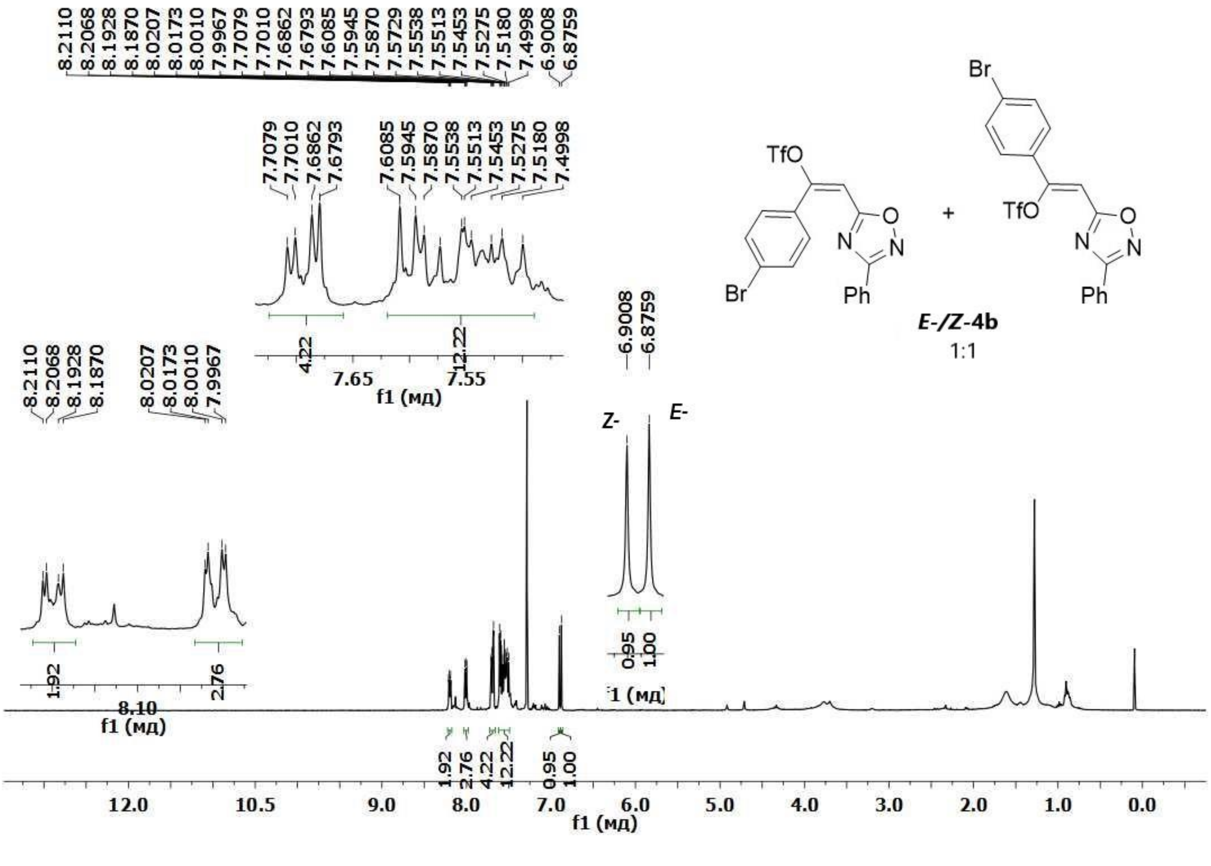

Figure S36. ^{1}H NMR spectrum of mixture of compounds ***E-/Z*-4b** (400 MHz, $CDCl_3$).

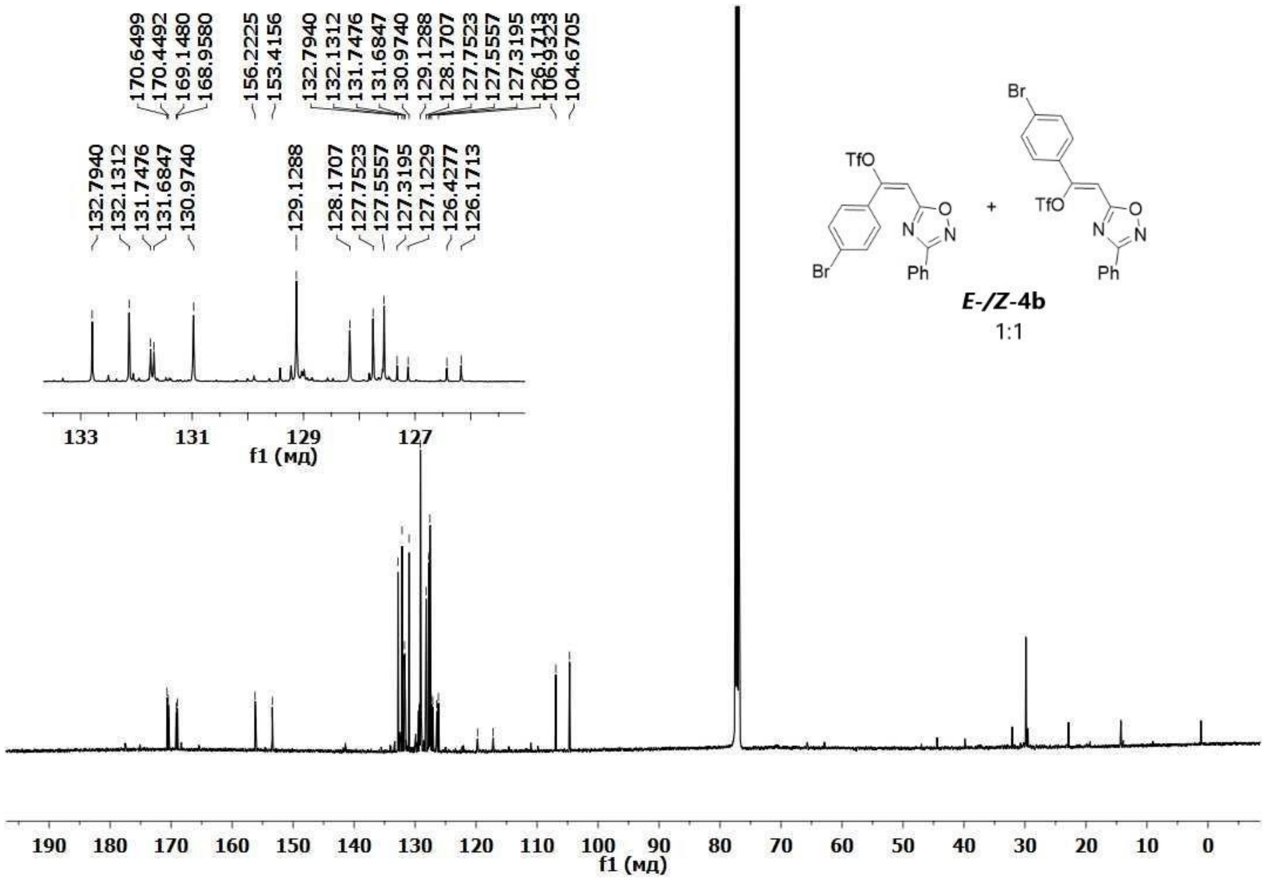

Figure S37. ^{13}C NMR spectrum of mixture of compounds ***E-/Z*-4b** (100 MHz, $CDCl_3$).

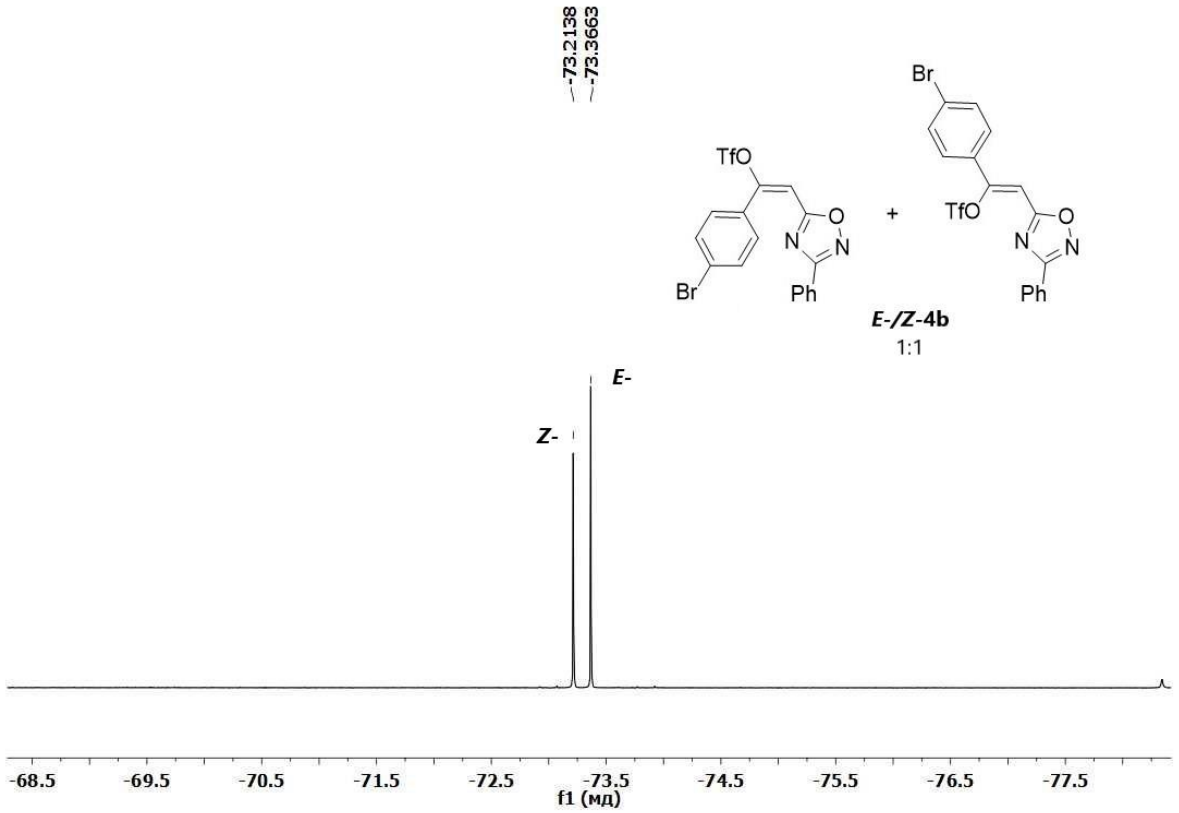

Figure S38. ^{19}F NMR spectrum of mixture of compounds ***E-/Z*-4a** (376 MHz, $CDCl_3$).

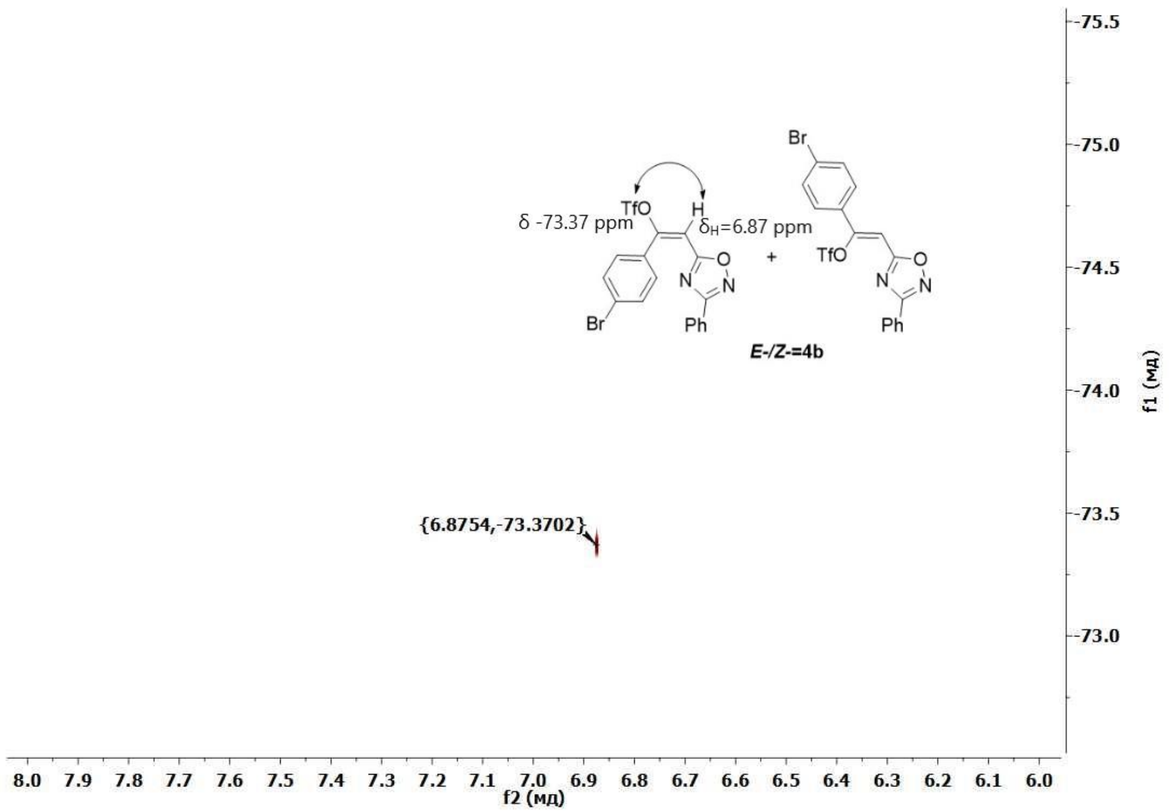

Figure S39. HOESY NMR spectrum of mixture of compounds ***E-/Z*-4b** ($CDCl_3$).

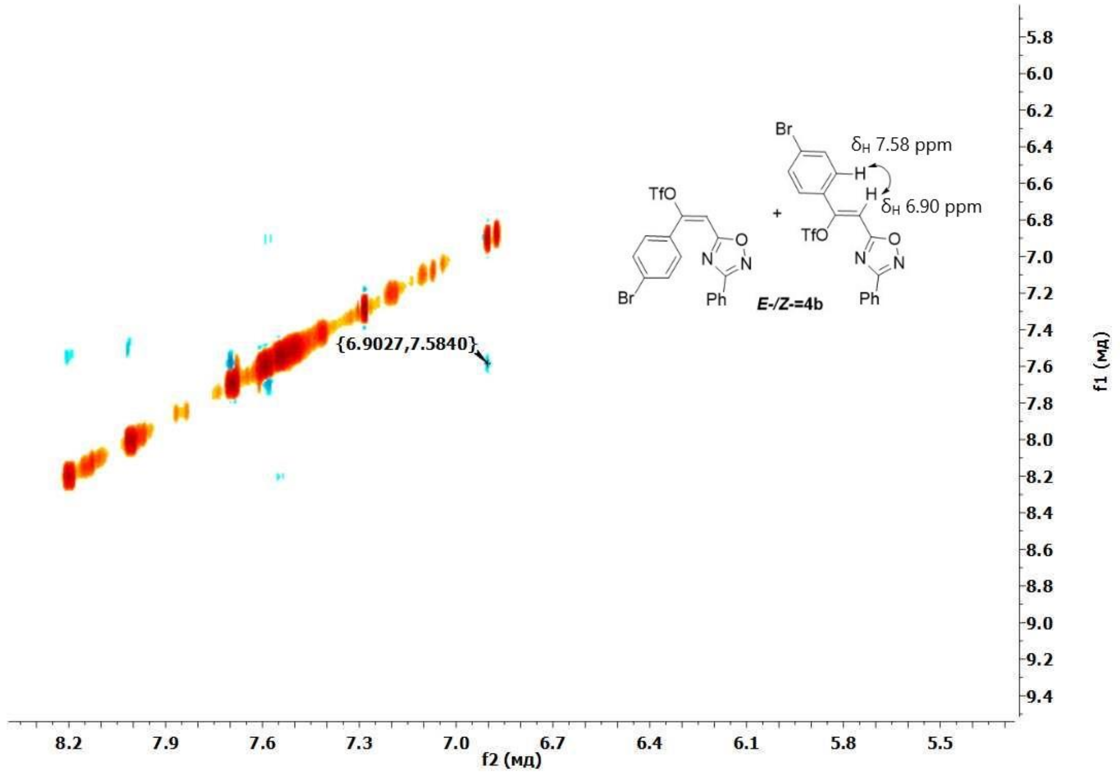

Figure S40. NOESY NMR spectrum of mixture of compounds ***E-/Z*-4b** ($CDCl_3$).

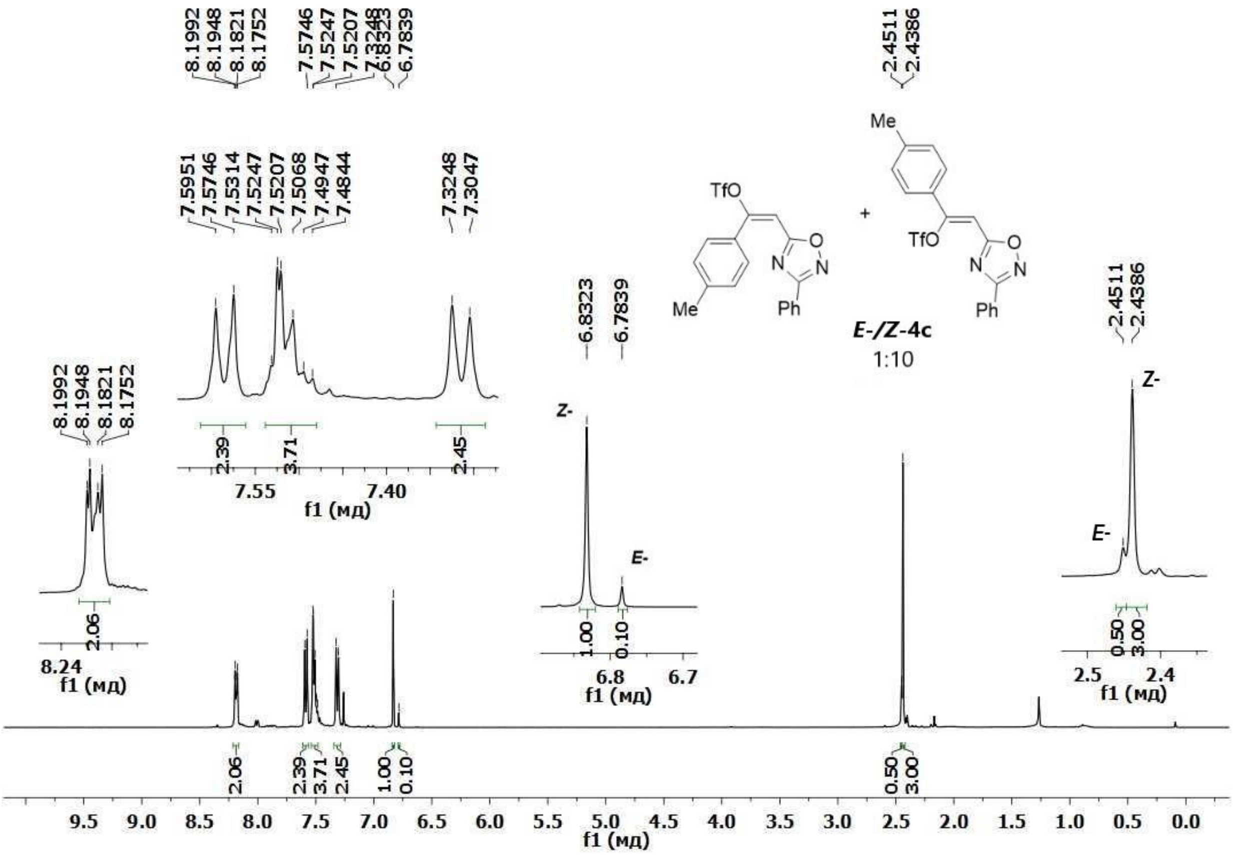

Figure S41. ^{1}H NMR spectrum of mixture of compounds ***E*-/*Z*-4c** (400 MHz, $CDCl_3$).

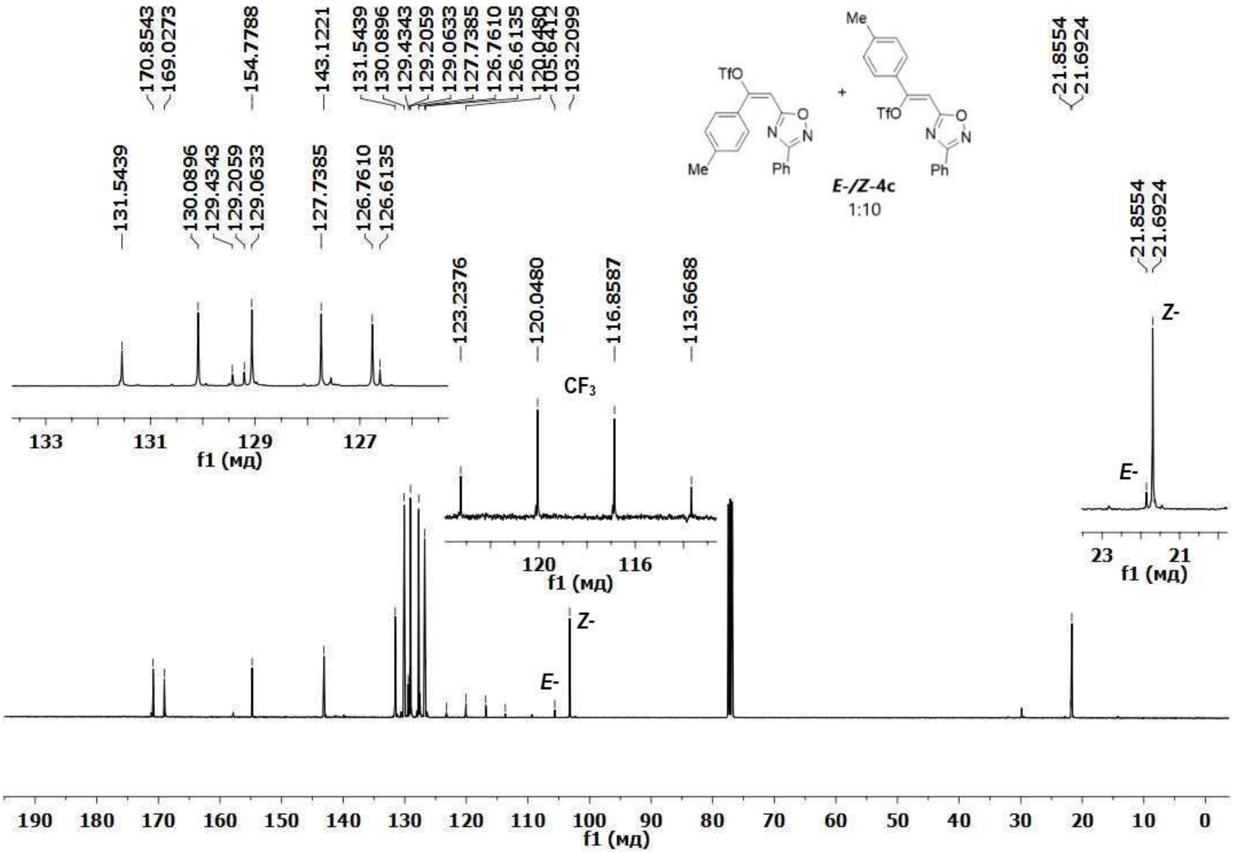

Figure S42. ^{13}C NMR spectrum of mixture of compounds ***E*-/*Z*-4c** (100 MHz, $CDCl_3$).

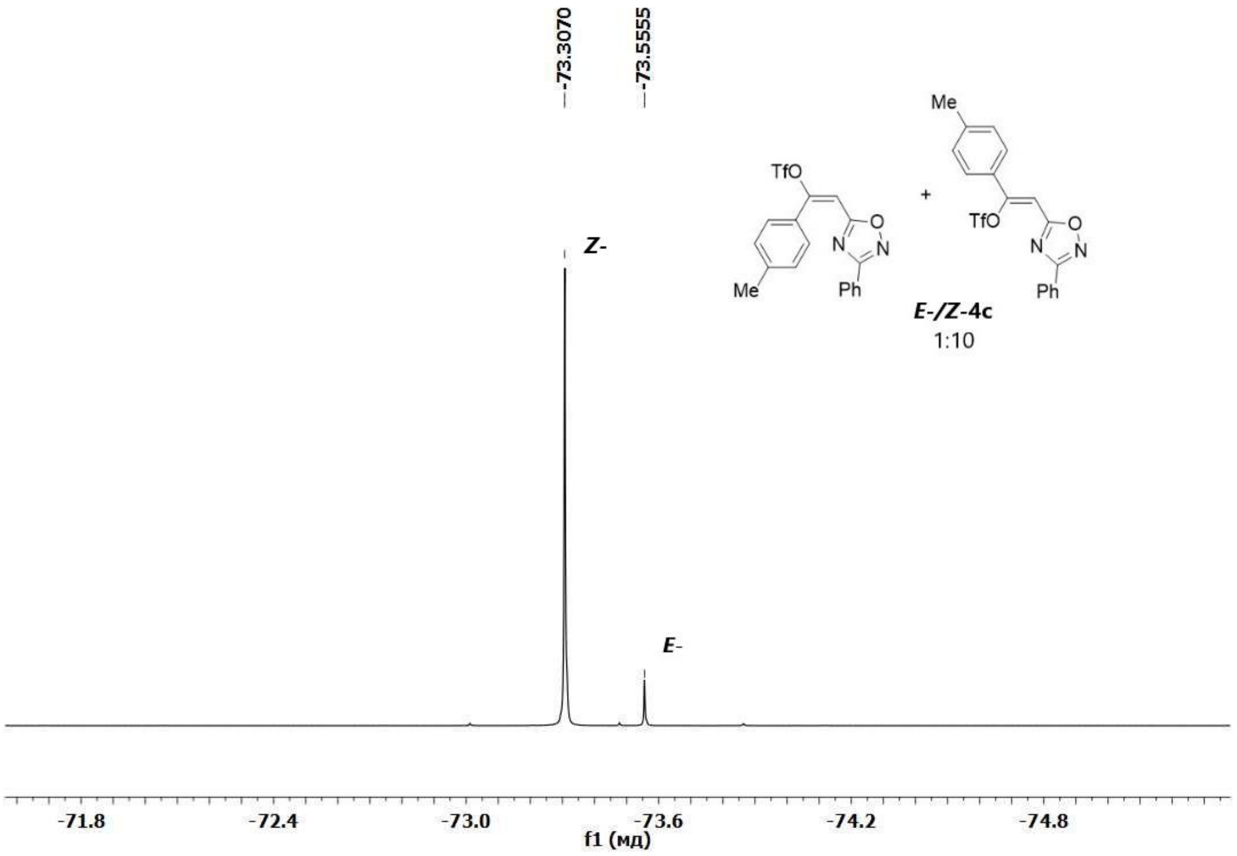

Figure S43. ^{19}F NMR spectrum of mixture of compounds ***E-/Z*-4c** (376 MHz, $CDCl_3$).

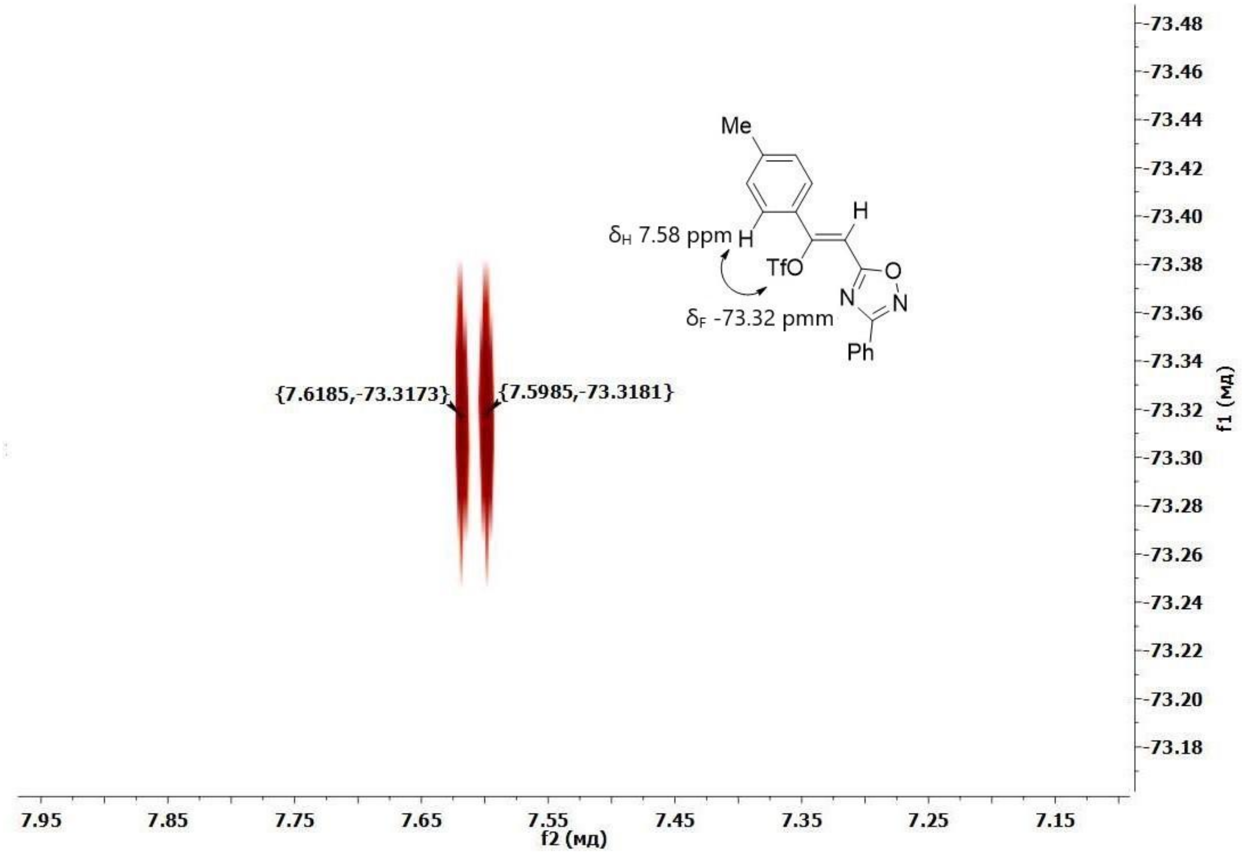

Figure S44. HOESY NMR spectrum of mixture of compounds ***E-/Z*-4c** ($CDCl_3$).

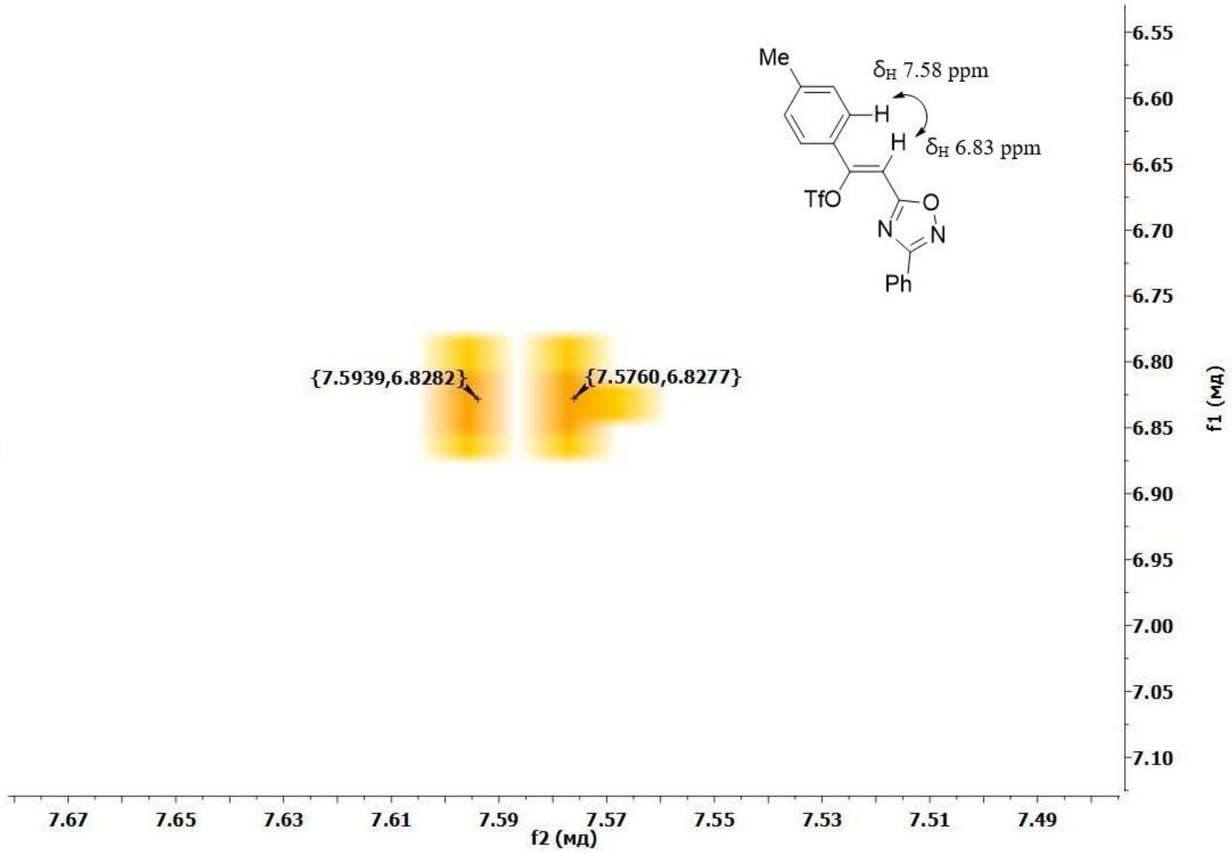

Figure S45. NOESY NMR spectrum of mixture of compounds ***E-/Z*-4c** ($CDCl_3$).

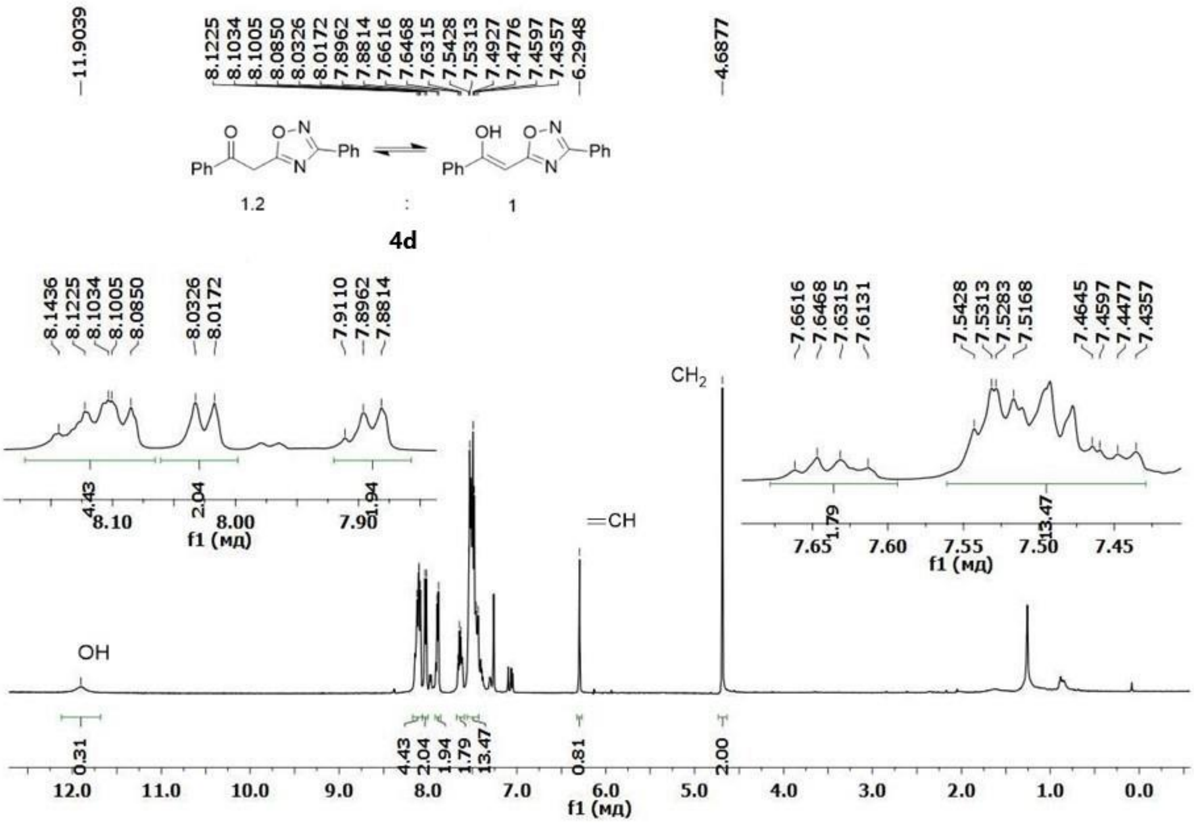

Figure S46. ^{1}H NMR spectrum of mixture of compounds **4d and 4d'** (500 MHz, $CDCl_3$).

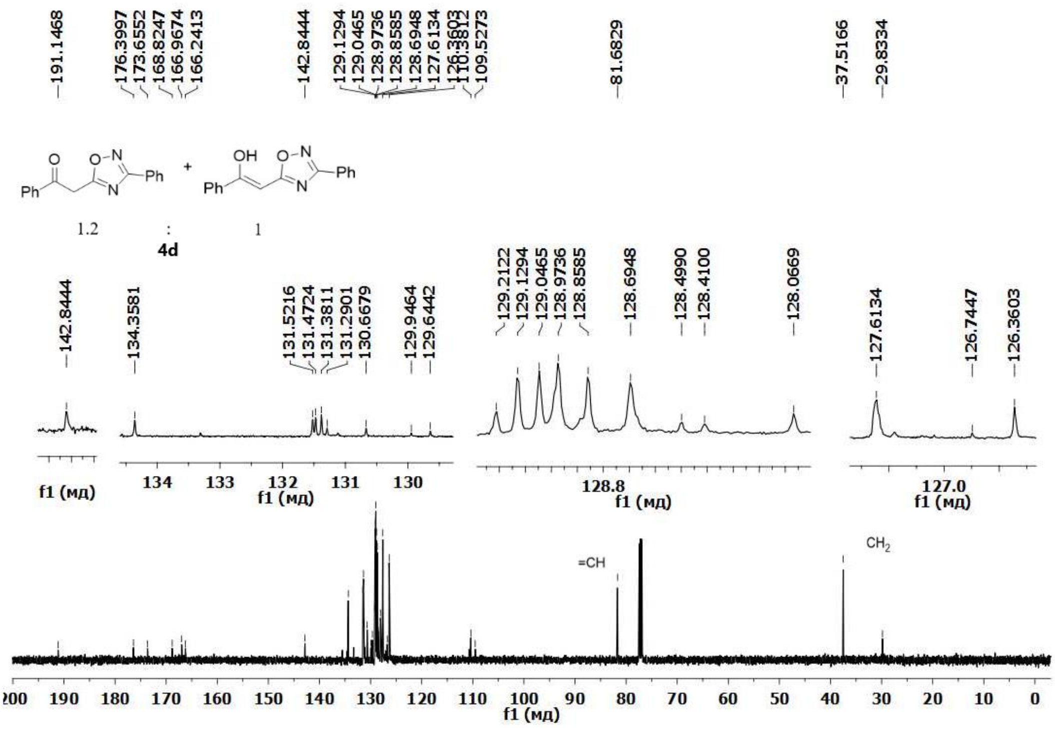

Figure S47. ^{13}C NMR spectrum of mixture of compounds **4d and 4d’** (125 MHz, $CDCl_3$).

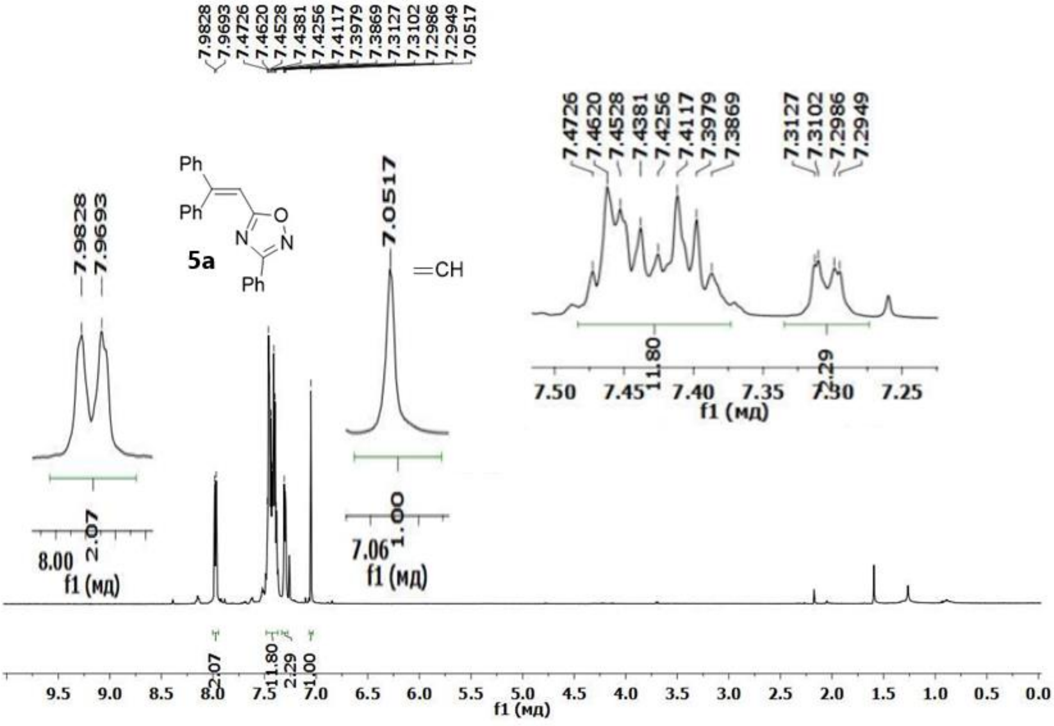

Figure S48. ^{1}H NMR spectrum of compound **5a** (500 MHz, $CDCl_3$).

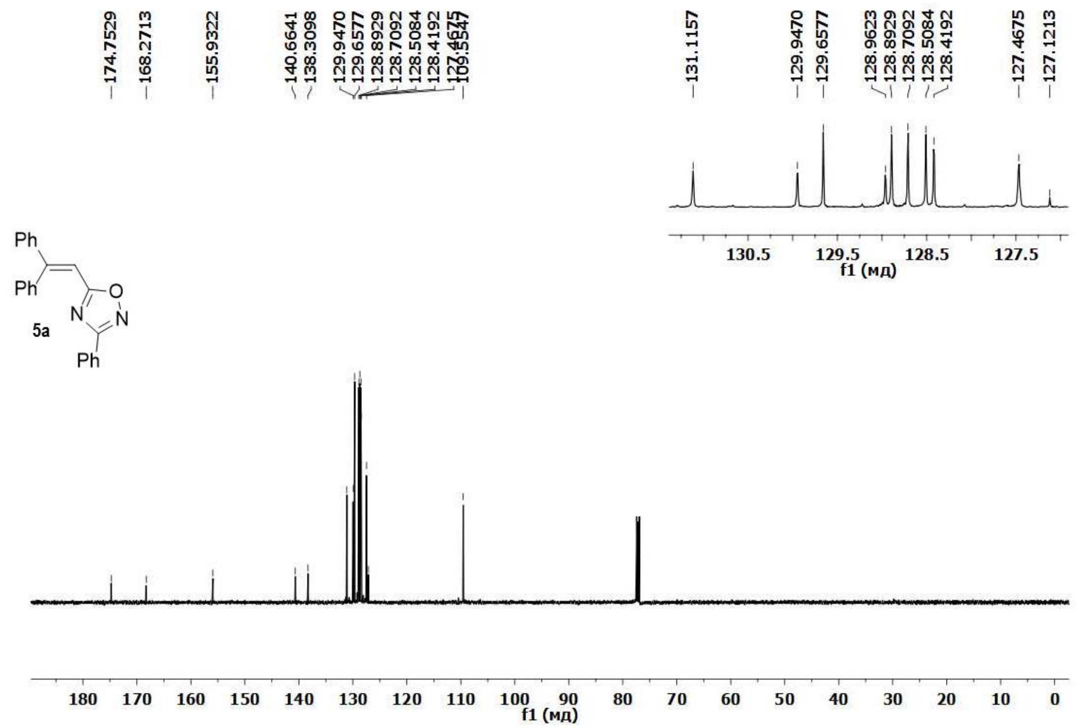

Figure S49. ^{13}C NMR spectrum of compound **5a** (125 MHz, $CDCl_3$).

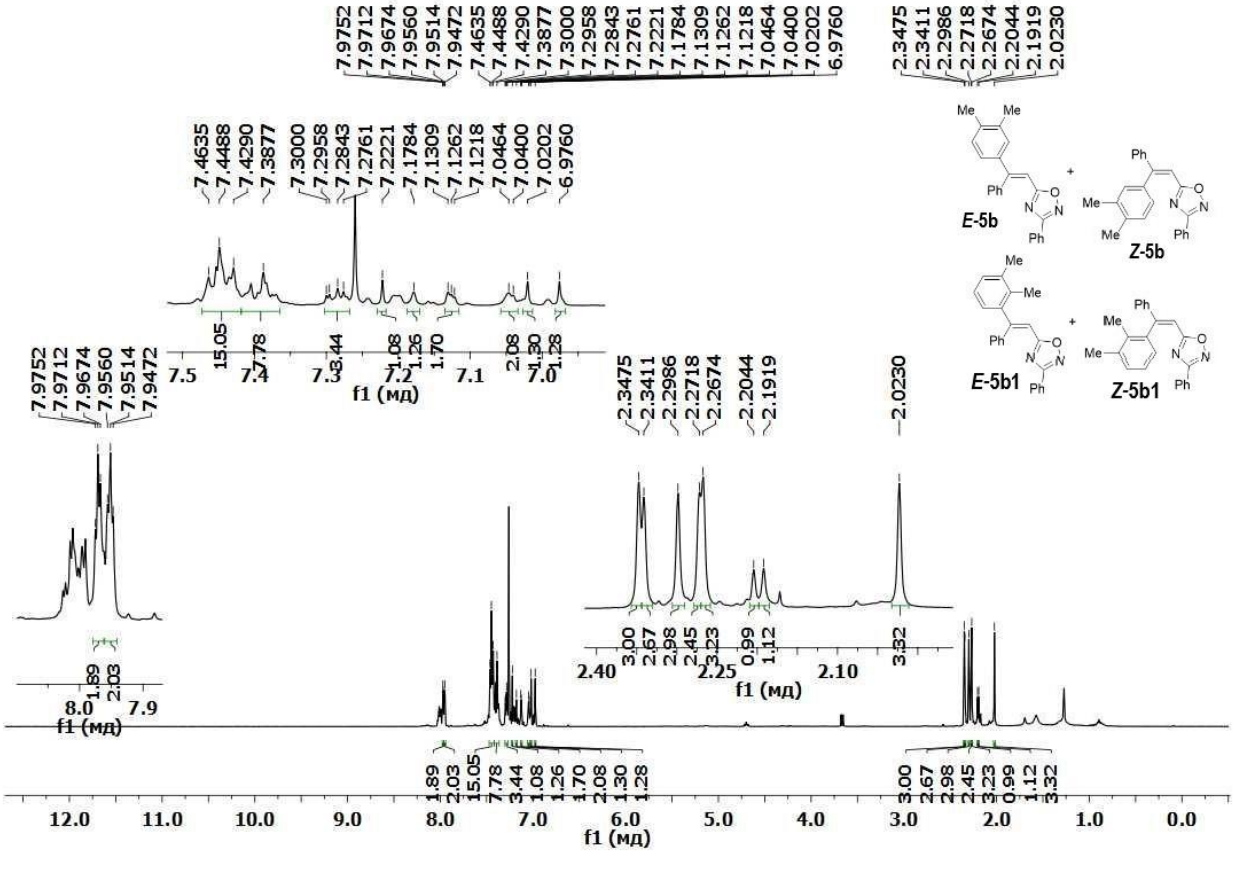

Figure S50. ^{1}H NMR spectrum of mixture of compounds ***E*-/-*Z*-5b** and ***E*-/-*Z*-5b1** (400 MHz, $CDCl_3$).

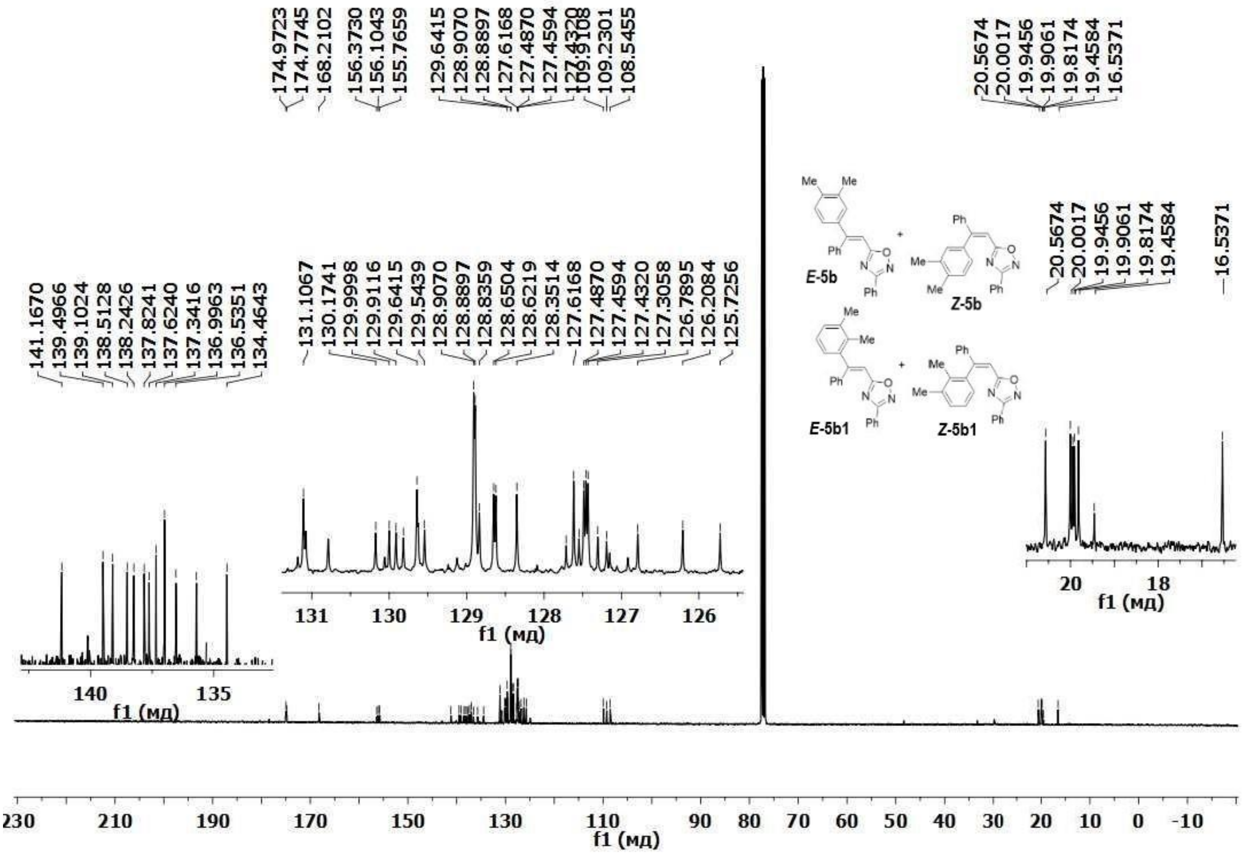

Figure S51. ^{13}C NMR spectrum of mixture of compounds ***E*-/-*Z*-5b** and ***E*-/-*Z*-5b1** (100 MHz, $CDCl_3$).

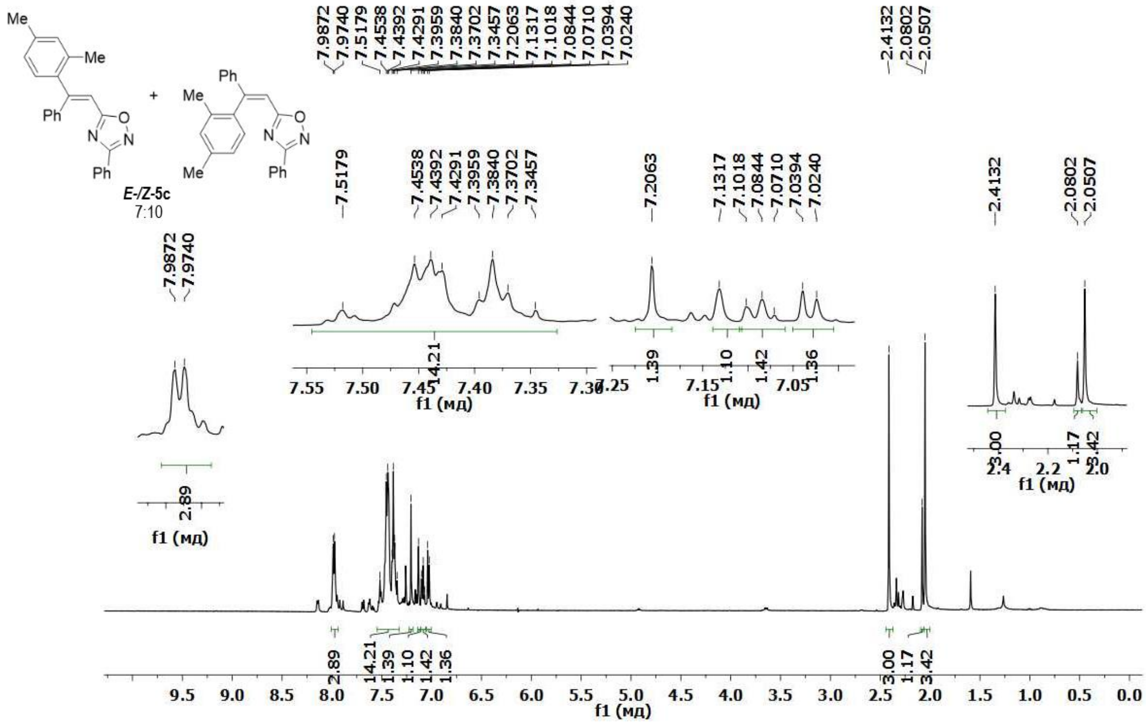

Figure S52. ^{1}H NMR spectrum of mixture of compounds ***E*-/*Z*-5c** (500 MHz, $CDCl_3$).

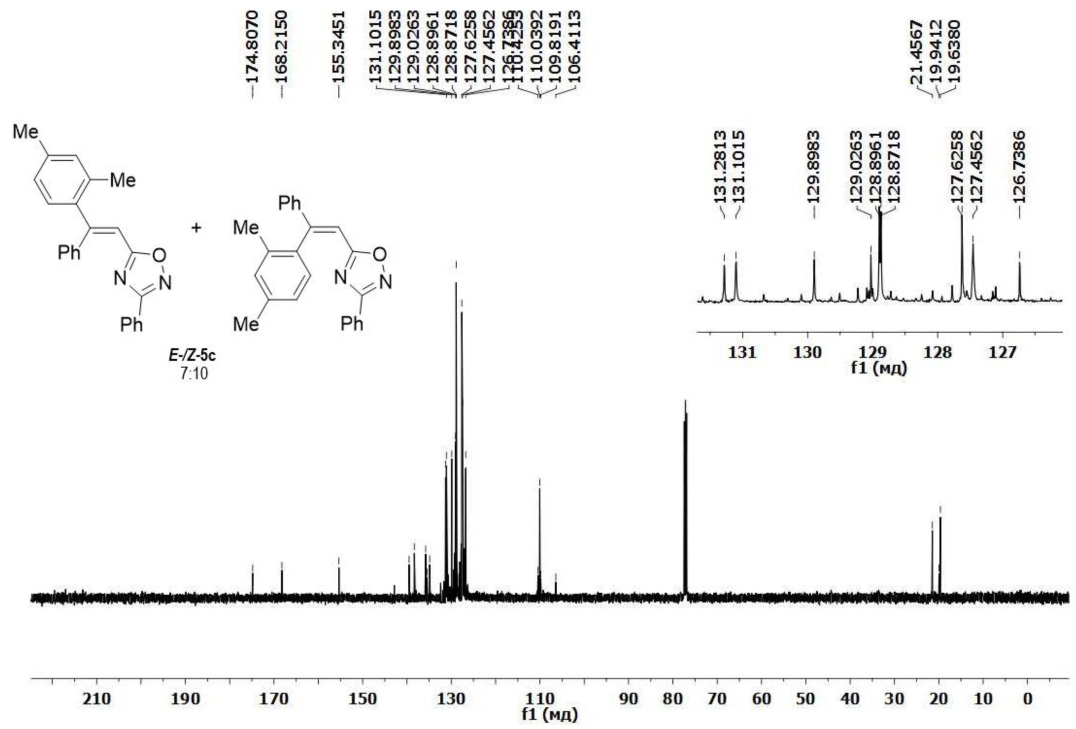

Figure S53. ^{13}C NMR spectrum of mixture of compounds ***E-/Z*-5c** (125 MHz, $CDCl_3$).

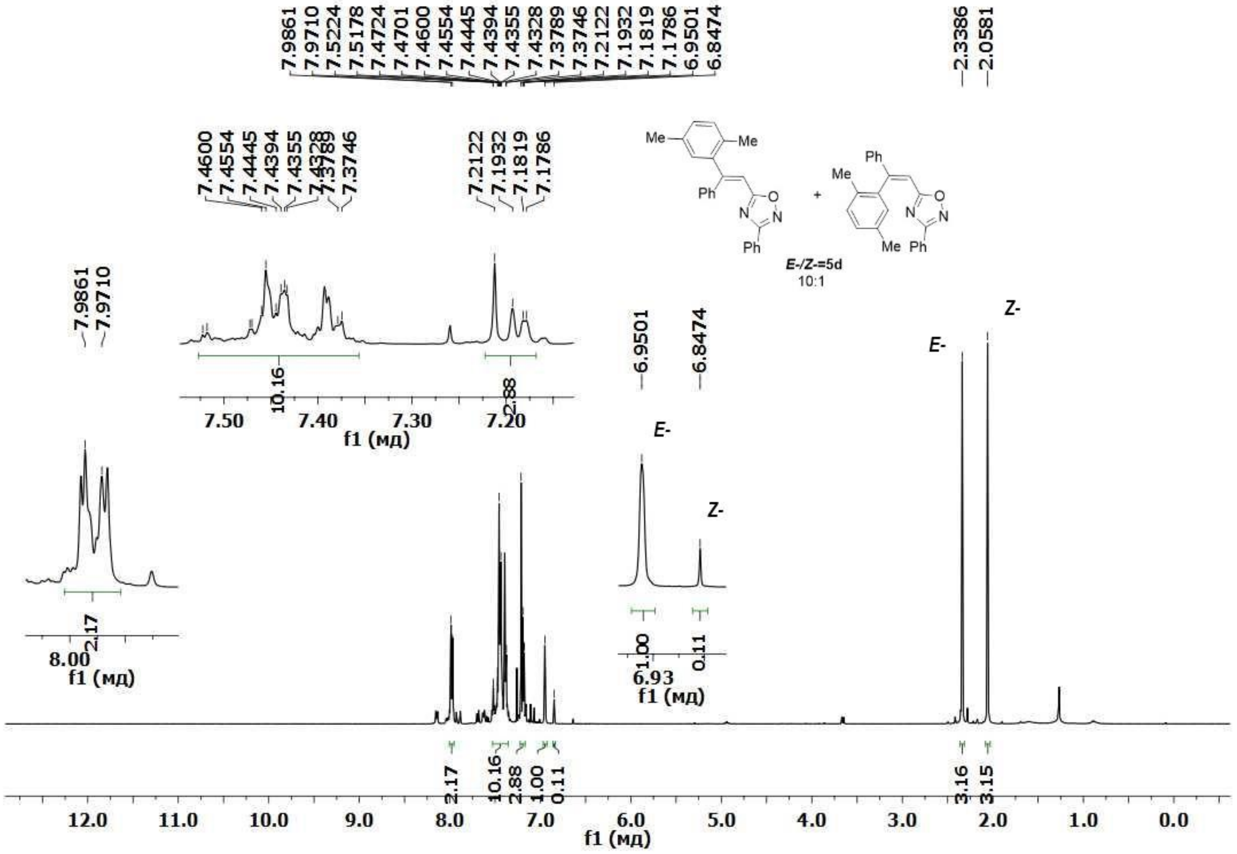

Figure S54. ^{1}H NMR spectrum of mixture of compounds ***E-/Z*-5d** (500 MHz, $CDCl_3$).

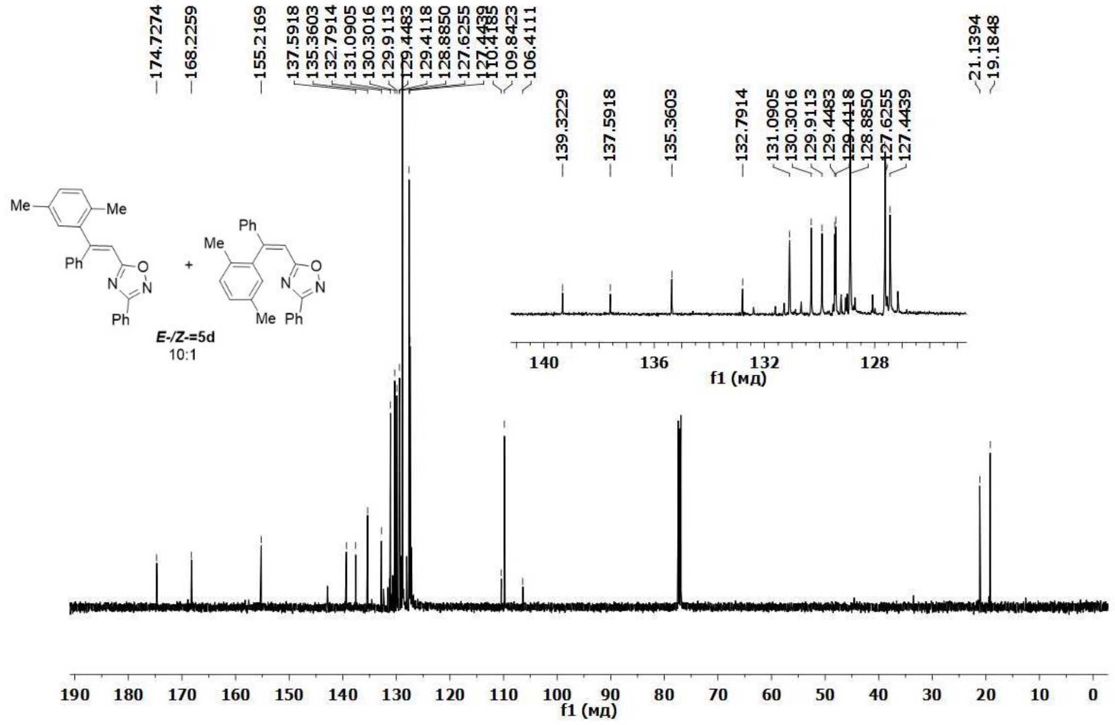

Figure S55. ^{13}C NMR spectrum of mixture of compounds ***E*-/*Z*-5d** (125 MHz, $CDCl_3$).

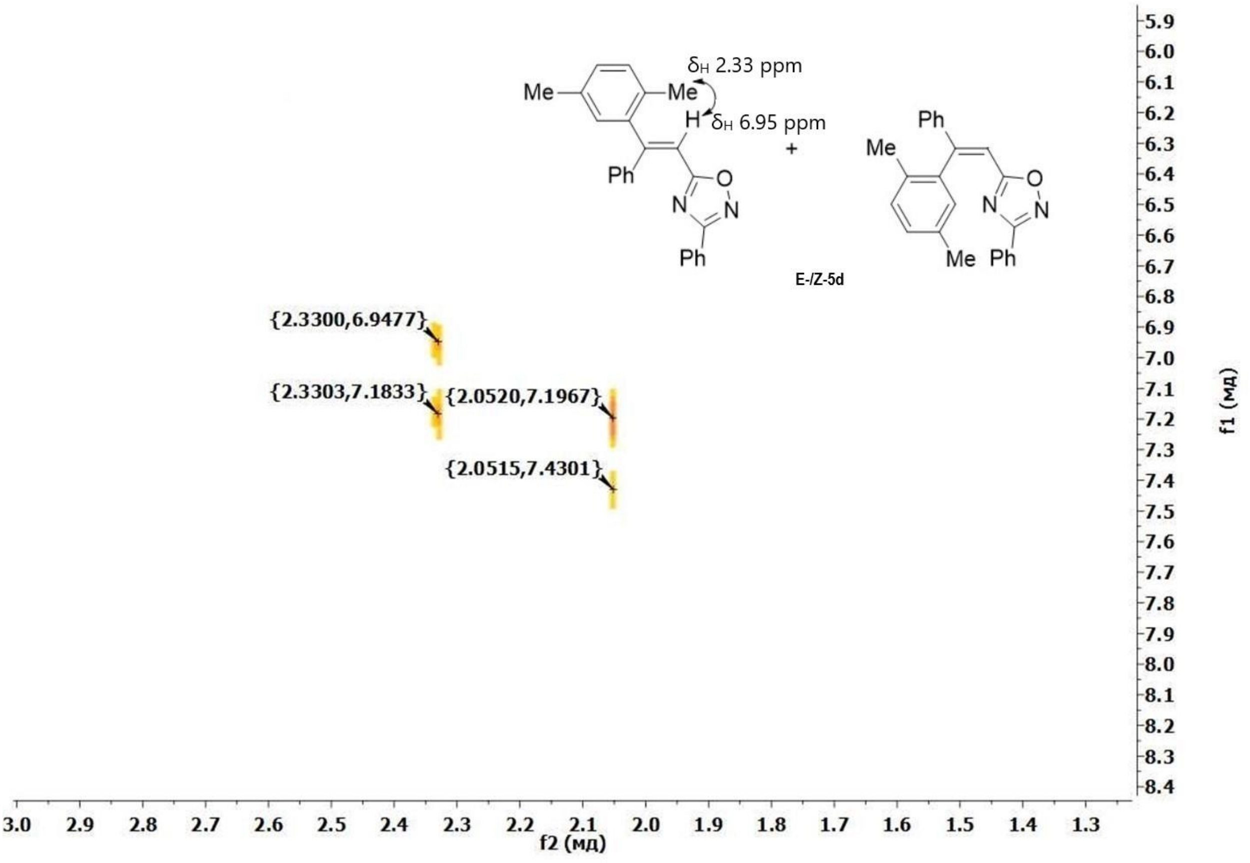

Figure S56. NOESY NMR spectrum of mixture of compounds ***E*-/*Z*-5d** ($CDCl_3$).

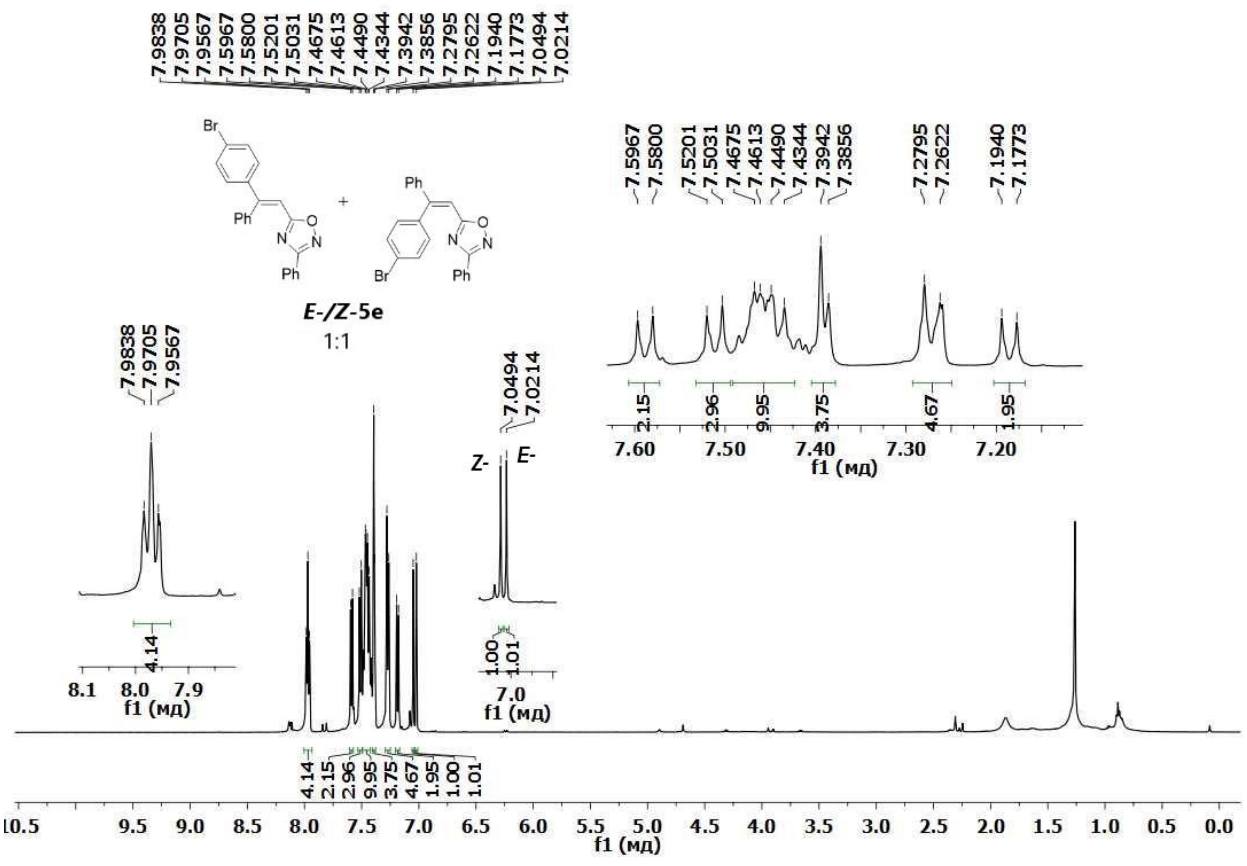

Figure S57. ^{1}H NMR spectrum of mixture of compounds ***E-/Z*-5e** (400 MHz, $CDCl_3$).

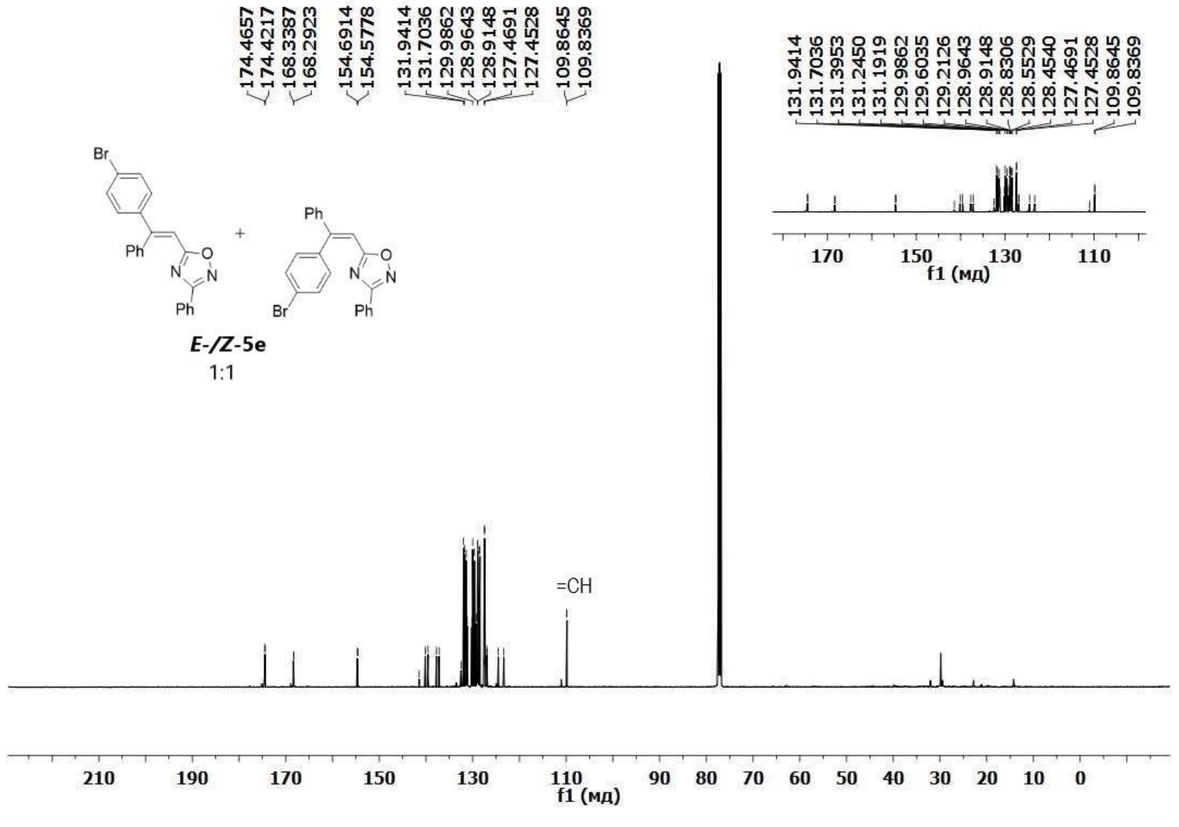

Figure S58. ^{13}C NMR spectrum of mixture of compounds ***E-/Z*-5e** (100 MHz, $CDCl_3$).

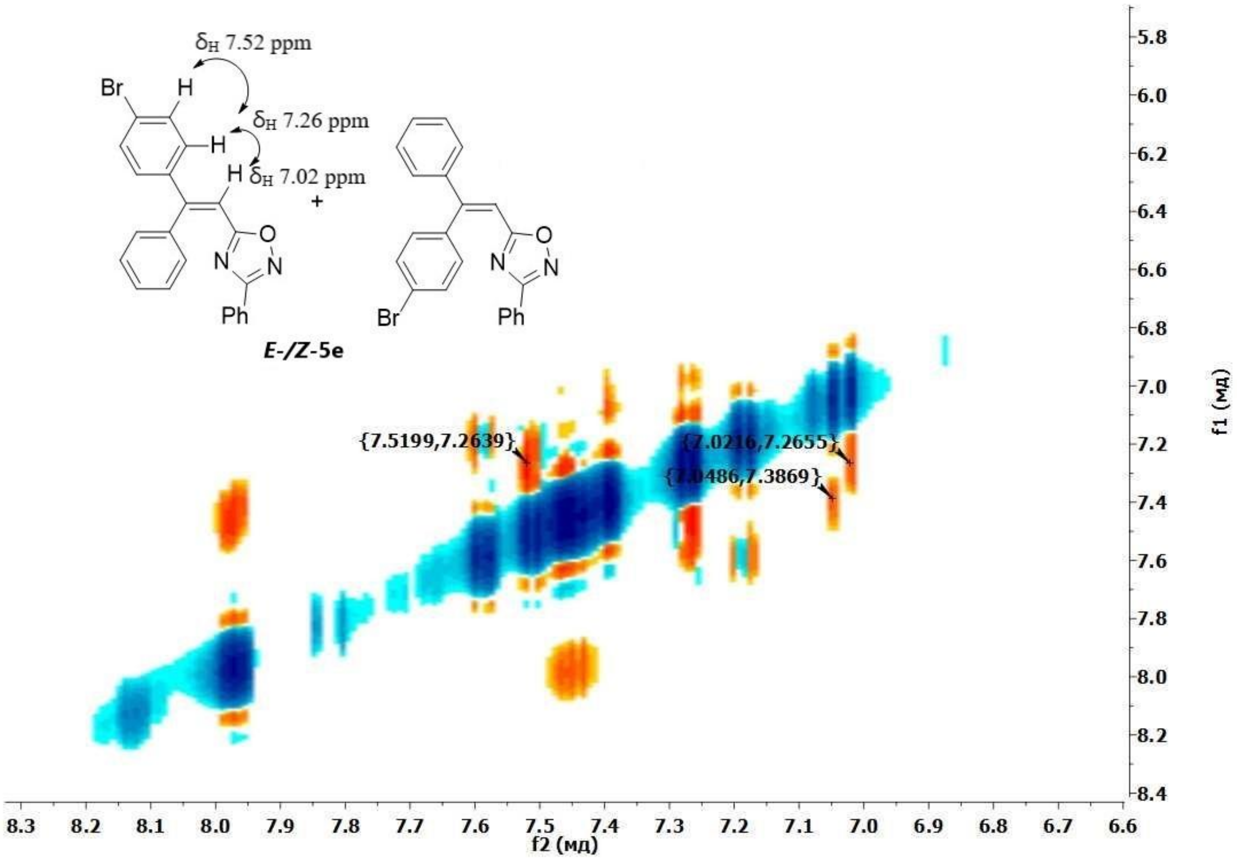

Figure S59. NOESY NMR spectrum of mixture of compounds ***E-/Z*-5e** ($CDCl_3$).

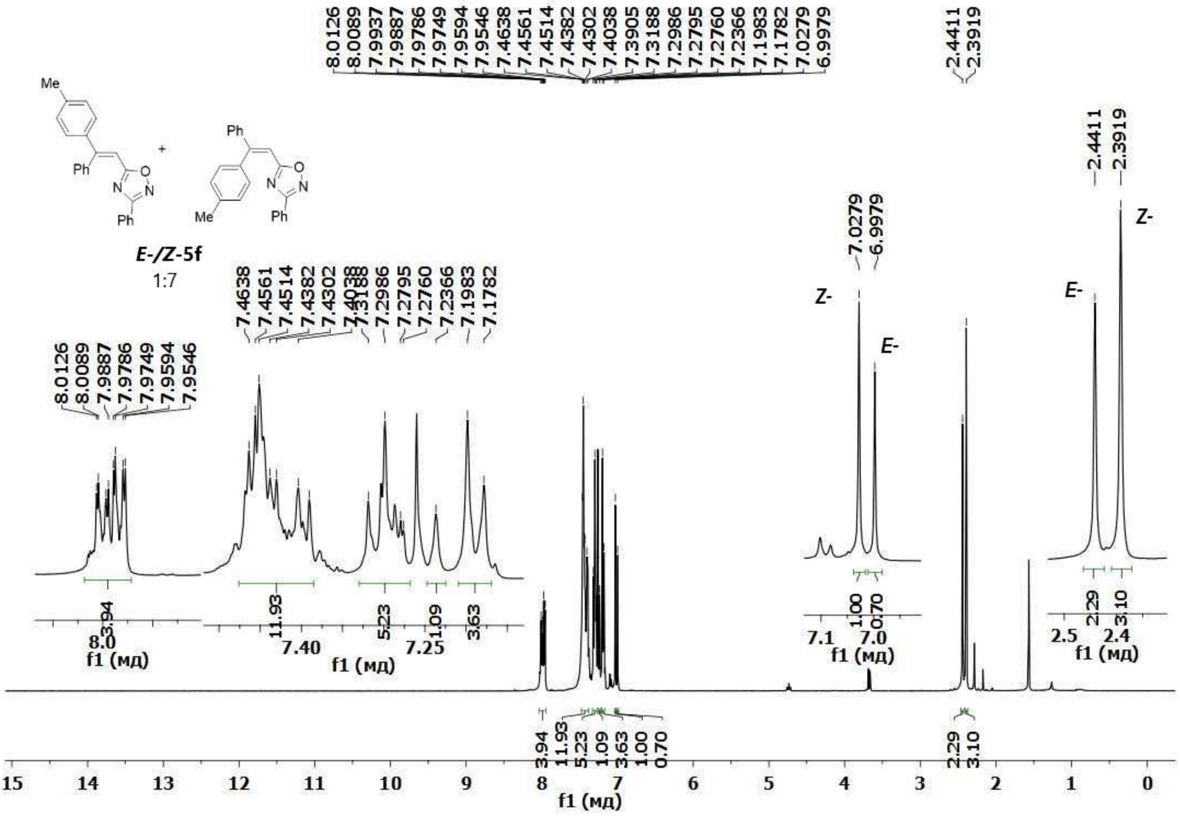

Figure S60. ^{1}H NMR spectrum of mixture of compounds ***E-/Z*-5f** (400 MHz, $CDCl_3$).

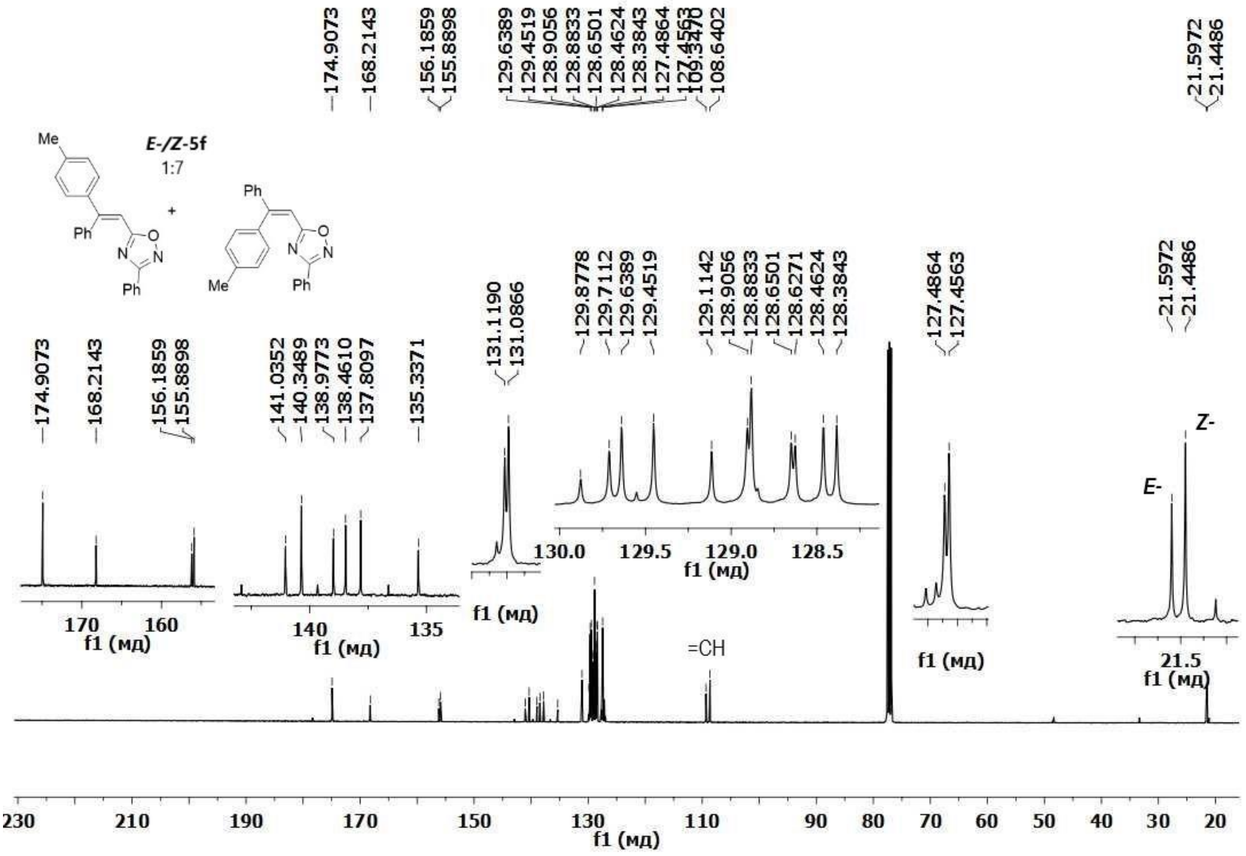

Figure S61. ^{13}C NMR spectrum of mixture of compounds ***E*-/*Z*-5f** (100 MHz, $CDCl_3$).

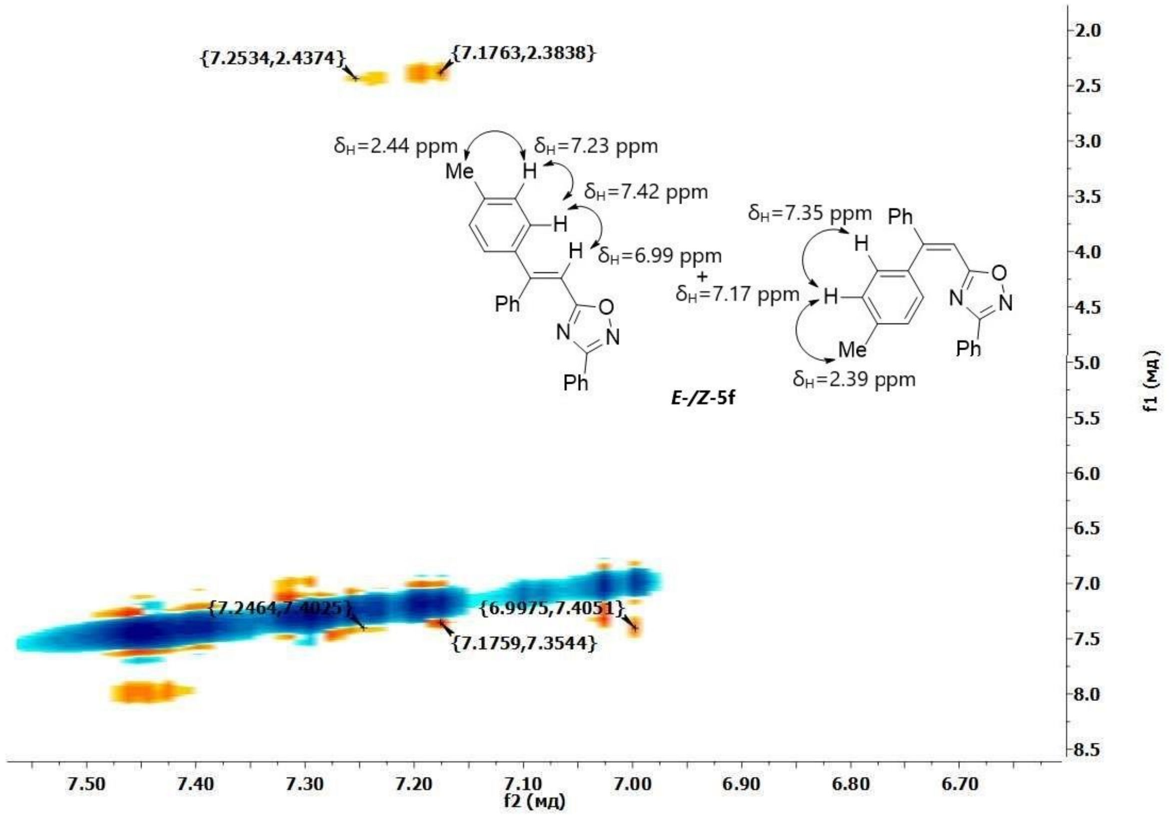

Figure S62. NOESY NMR spectrum of mixture of compounds ***E*-/*Z*-5f** ($CDCl_3$).

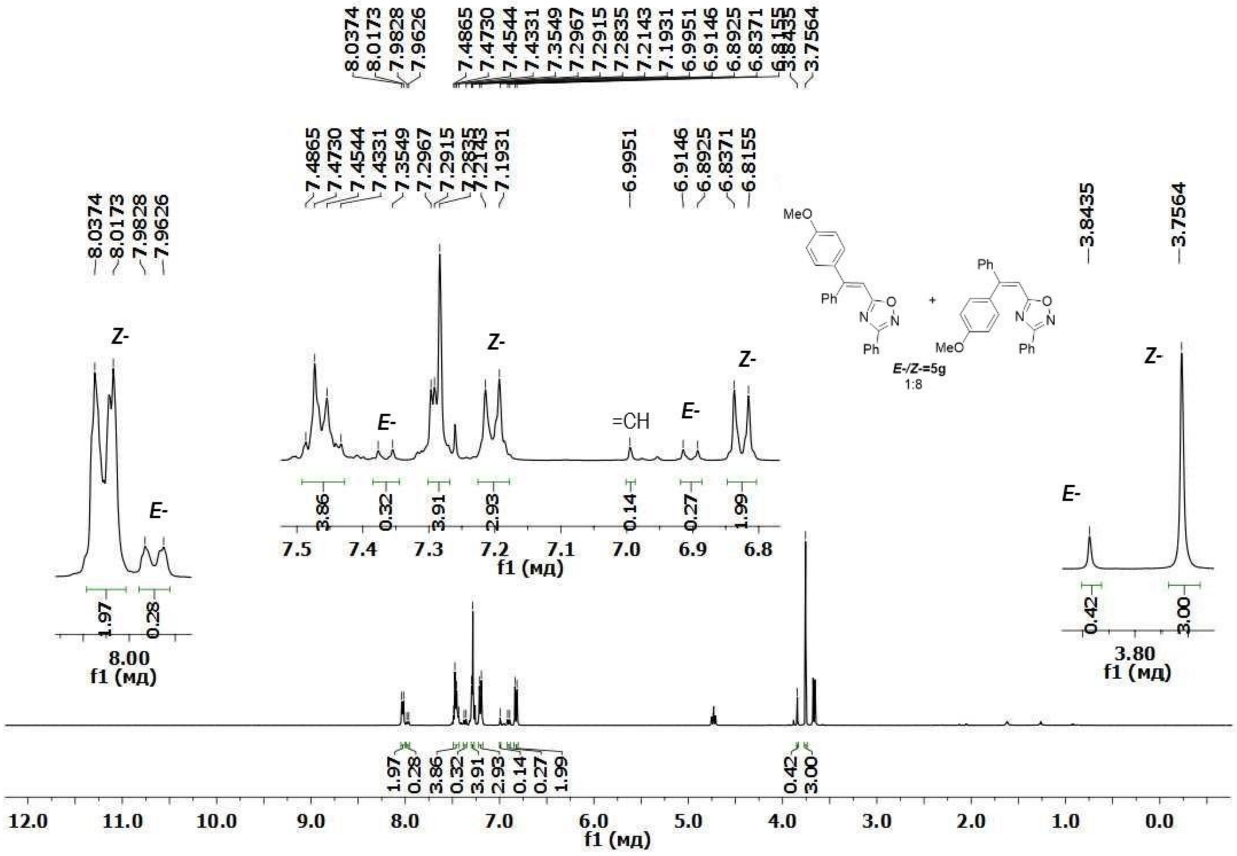

Figure S63. ^{1}H NMR spectrum of mixture of compounds ***E-/Z*-5g** (400 MHz, $CDCl_3$).

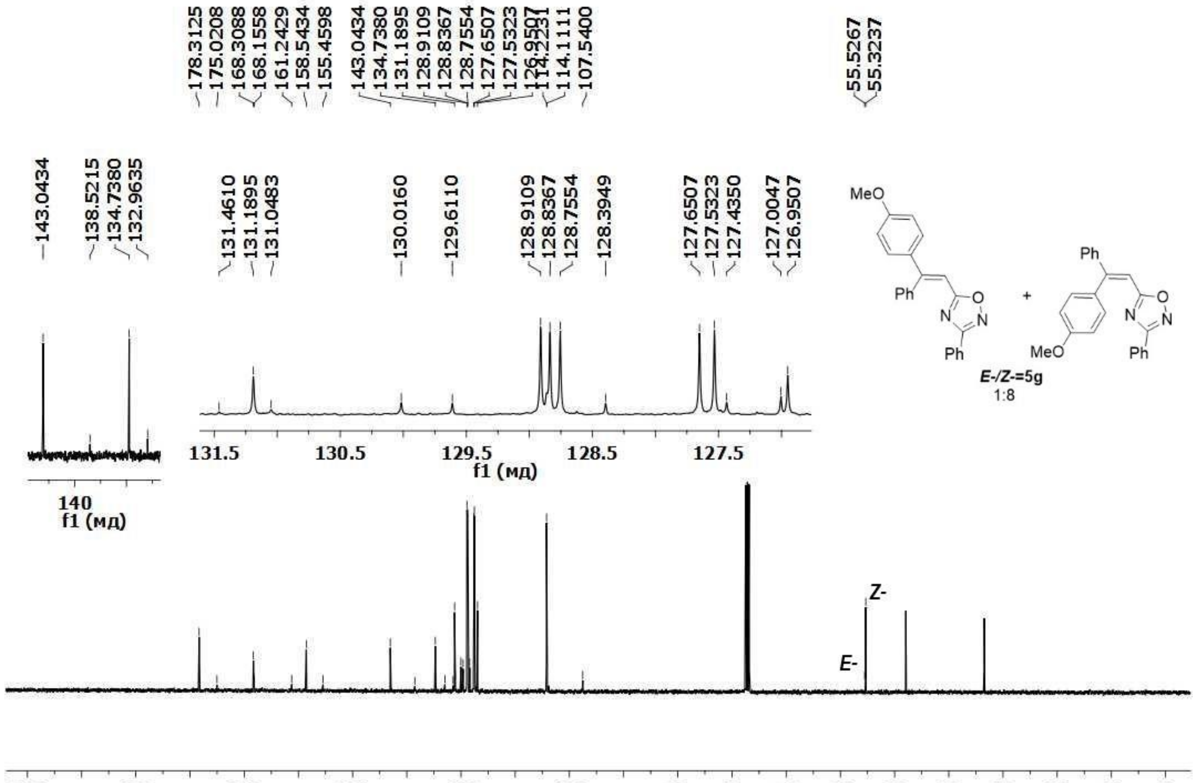

Figure S64. ^{13}C NMR spectrum of mixture of compounds ***E-/Z*-5g** (100 MHz, $CDCl_3$).

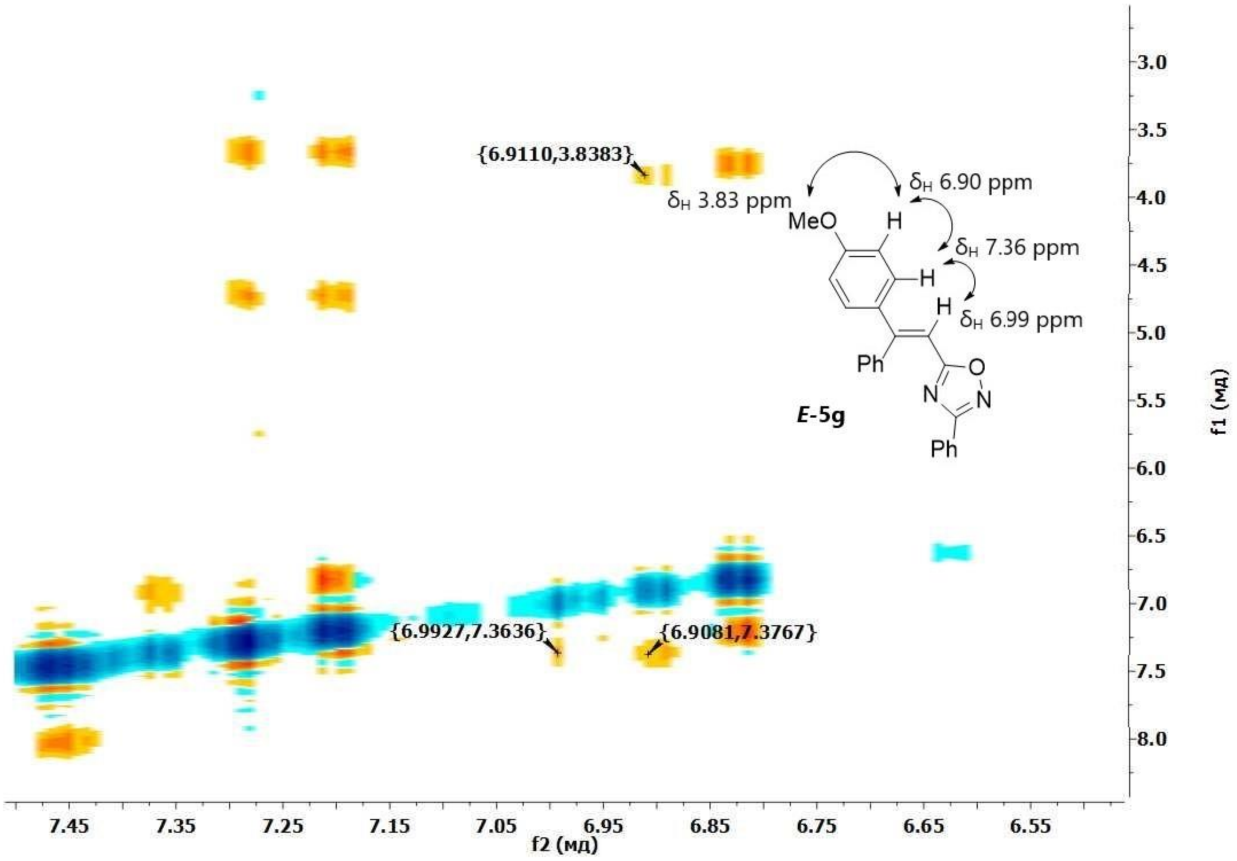

Figure S65. NOESY NMR spectrum of mixture of compounds ***E*-/*Z*-5g** ($CDCl_3$).

4. Data of DFT calculation of compounds 3 and cations A, B

All computations were carried out at the DFT/HF hybrid level of theory using hybrid potential M062X by using GAUSSIAN 2009 program packages [1]. The geometries optimization were performed using the 6-311+G(2d,2p) basis set (standard 6-311G basis set added with polarization (d,p) and diffuse functions). Optimizations were performed on all degrees of freedom and solvent phase optimized structures were verified as true minima with no imaginary frequencies. The Hessian matrix was calculated analytically for the optimized structures in order to prove the location of correct minima and to estimate the thermodynamic parameters. Solvent-phase calculations used the Polarizable Continuum Model (PCM, solvent = water).

[1] Frisch, M. J.; Trucks, G. W.; Schlegel, H. B.; Scuseria, G. E.; Robb, M. A.; Cheeseman, J. R.; Scalmani, G.; Barone, V.; Mennucci, B.; Petersson, G. A.; Nakatsuji, H.; Caricato, M.; Li, X.; Hratchian, H. P.; Izmaylov, A. F.; Bloino, J.; Zheng, G.; Sonnenberg, J. L.; Hada, M.; Ehara, M.; Toyota, K.; Fukuda, R.; Hasegawa, J.; Ishida, M.; Nakajima, T.; Honda, Y.; Kitao, O.; Nakai, H.; Vreven, T.; Montgomery, Jr., J. A.; Peralta, J. E.; Ogliaro, F.; Bearpark, M.; Heyd, J. J.; Brothers, E.; Kudin, K. N.; Staroverov, V. N.; Keith, T.; Kobayashi, R.; Normand, J.; Raghavachari, K.; Rendell, A.; Burant, J. C.; Iyengar, S. S.; Tomasi, J.; Cossi, M.; Rega, N.; Millam, J. M.; Klene, M.; Knox, J. E.; Cross, J. B.; Bakken, V.; Adamo, C.; Jaramillo, J.; Gomperts, R.; Stratmann, R. E.; Yazyev, O.; Austin, A. J.; Cammi, R.; Pomelli, C.; Ochterski, J. W.; Martin, R. L.; Morokuma, K.; Zakrzewski, V. G.; Voth, G. A.; Salvador, P.; Dannenberg, J. J.; Dapprich, S.; Daniels, A. D.; Farkas, O.; Foresman, J. B.; Ortiz, J. V.; Cioslowski, J.; Fox, D. J.; *Gaussian 09, Revision C.01*, Gaussian, Inc., Wallingford CT, **2010**.

1a

Energy E(B3LYP) = -800.60145468 h, G^{298} = -800.429214 h, μ=3.93 D

Cartesian coordinates, Å

N	atom	x	y	z
1	N	-1.070075	0.254421	0.000039
2	C	-2.193715	-0.542200	0.000030
3	C	-0.096120	-0.610830	0.000119
4	N	-1.930155	-1.827663	0.000084
5	O	-0.529881	-1.889326	0.000070
6	C	1.288394	-0.372066	0.000128
7	C	2.478402	-0.168115	0.000138
8	C	3.876398	0.083356	0.000045
9	C	4.355247	1.404223	-0.000034
10	C	4.789092	-0.984698	0.000040
11	C	5.721178	1.645980	-0.000116
12	C	6.152823	-0.730069	-0.000042
13	C	6.621269	0.582269	-0.000121
14	H	3.653179	2.225315	-0.000029
15	H	4.420984	-2.000345	0.000102
16	H	6.084421	2.663898	-0.000176
17	H	6.851061	-1.555059	-0.000045
18	H	7.684859	0.775393	-0.000185
19	C	-3.559884	-0.004584	-0.000012
20	C	-4.666551	-0.862336	-0.000028
21	C	-3.761024	1.378738	-0.000037
22	C	-5.952085	-0.339512	-0.000068
23	C	-5.051251	1.896677	-0.000076
24	C	-6.148394	1.040674	-0.000092
25	H	-4.517180	-1.932170	-0.000010
26	H	-2.907952	2.040584	-0.000026
27	H	-6.801927	-1.007602	-0.000081
28	H	-5.198220	2.967633	-0.000095
29	H	-7.151246	1.444606	-0.000123

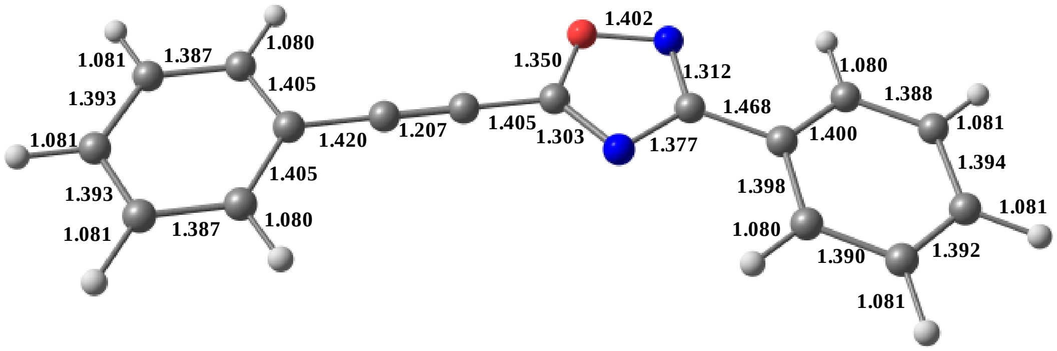

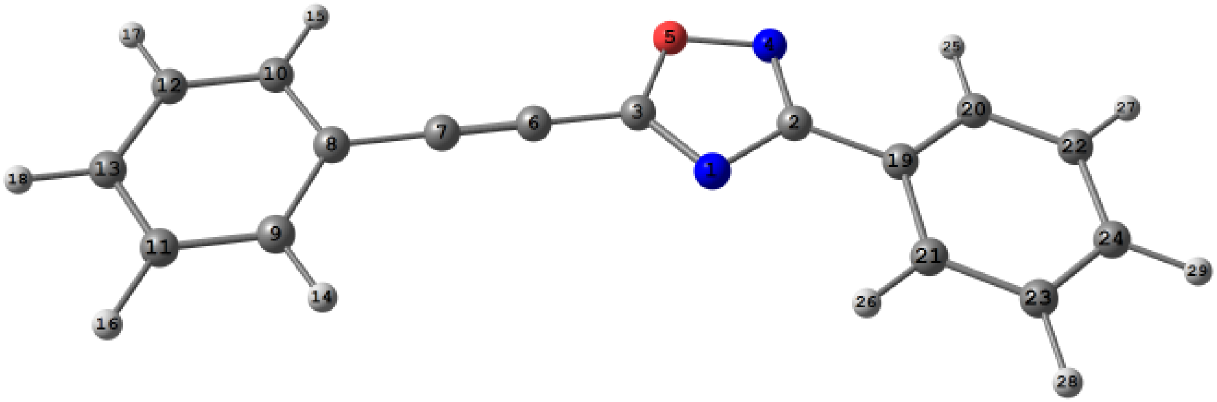

Summary of Natural Population Analysis:

Atom	No	Natural Charge	Natural Population: Core	Valence	Rydberg	Total
N	1	-0.55015	1.99938	5.49362	0.05716	7.55015
C	2	0.33370	1.99917	3.61485	0.05227	5.66630
C	3	0.26787	1.99902	3.53573	0.19738	5.73213
N	4	-0.19270	1.99933	5.15361	0.03976	7.19270
O	5	-0.34802	1.99973	6.30828	0.04001	8.34802
C	6	0.82585	1.99834	2.55859	0.61722	5.17415
C	7	-0.42464	1.99842	4.27926	0.14696	6.42464
C	8	-0.20152	1.99895	4.15622	0.04635	6.20152
C	9	-0.15485	1.99909	4.13245	0.02331	6.15485
C	10	-0.15586	1.99909	4.13402	0.02275	6.15586
C	11	-0.20300	1.99915	4.18352	0.02033	6.20300
C	12	-0.20287	1.99915	4.18329	0.02043	6.20287
C	13	-0.17782	1.99916	4.15804	0.02062	6.17782

H	14	0.22147	0.00000	0.77507	0.00346	0.77853
H	15	0.22204	0.00000	0.77499	0.00297	0.77796
H	16	0.22115	0.00000	0.77702	0.00183	0.77885
H	17	0.22111	0.00000	0.77709	0.00180	0.77889
H	18	0.21884	0.00000	0.77956	0.00160	0.78116
C	19	-0.56629	1.99900	4.31305	0.25424	6.56629
C	20	-0.20259	1.99893	4.14312	0.06054	6.20259
C	21	0.22330	1.99896	2.56139	1.21635	5.77670
C	22	-0.26217	1.99916	4.17962	0.08338	6.26217
C	23	-0.67901	1.99920	4.41668	0.26313	6.67901
C	24	-0.25481	1.99913	4.16957	0.08611	6.25481
H	25	0.22006	0.00000	0.77542	0.00453	0.77994
H	26	0.08232	0.00000	0.79473	0.12295	0.91768
H	27	0.21638	0.00000	0.78026	0.00335	0.78362
H	28	0.21195	0.00000	0.78162	0.00643	0.78805
H	29	0.21368	0.00000	0.78213	0.00419	0.78632
* Total *		-0.87659	37.98236	87.47281	3.42142	128.87659

1b

Energy E(B3LYP) = -839.932109924 h, G^{298} = -839.736206 h, μ=4.94 D

Cartesian coordinates, Å

N	atom	x	y	z
1	N	-1.559172	0.238648	-0.002474
2	C	-2.696985	-0.536624	-0.001163
3	C	-0.600560	-0.644631	-0.003795
4	N	-2.458108	-1.826903	-0.001633
5	O	-1.058966	-1.915065	-0.003492
6	C	0.787111	-0.432181	-0.005160
7	C	1.981920	-0.253324	-0.005508
8	C	3.383517	-0.035500	-0.004802
9	C	3.901861	1.270261	-0.019558
10	C	4.275234	-1.120322	0.006362
11	C	5.271660	1.475368	-0.020789
12	C	5.642865	-0.896979	0.005336
13	C	6.167310	0.399957	-0.004964
14	H	3.225117	2.112470	-0.033087
15	H	3.888388	-2.129143	0.012873
16	H	5.654282	2.487207	-0.036670
17	H	6.316216	-1.743696	0.010183
18	C	-4.053113	0.026104	0.001058
19	C	-5.175670	-0.810830	-0.002767
20	C	-4.228793	1.412877	0.007461
21	C	-6.451335	-0.264279	-0.000065
22	C	-5.509197	1.954694	0.010226
23	C	-6.622056	1.119248	0.006490
24	H	-5.046110	-1.883246	-0.007889
25	H	-3.363605	2.058763	0.010373
26	H	-7.313422	-0.916505	-0.003117
27	H	-5.636291	3.028192	0.015299
28	H	-7.617271	1.541657	0.008614
29	C	7.653595	0.634556	0.024094
30	H	8.000007	0.770565	1.051424
31	H	8.197149	-0.210410	-0.395418
32	H	7.924041	1.532074	-0.530642

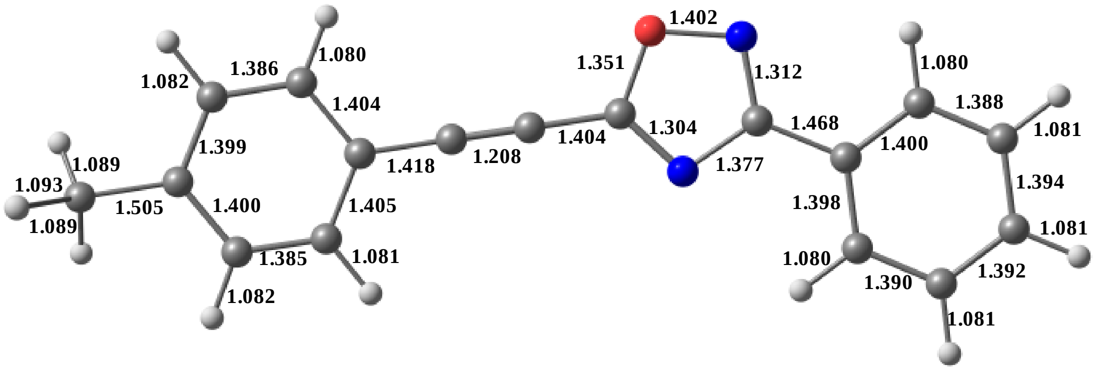

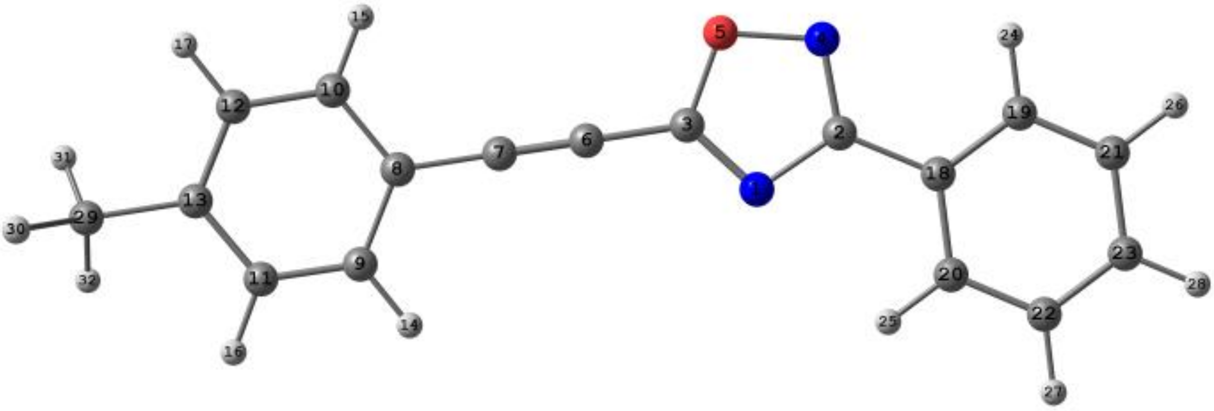

Summary of Natural Population Analysis:

Atom	No	Natural Charge	Core	Valence	Rydberg	Total
N	1	-0.52391	1.99934	5.48208	0.04248	7.52391
C	2	0.32832	1.99918	3.61680	0.05570	5.67168
C	3	0.49688	1.99907	3.46997	0.03407	5.50312
N	4	-0.19227	1.99933	5.15379	0.03914	7.19227
O	5	-0.32353	1.99972	6.29713	0.02668	8.32353
C	6	-0.09998	1.99828	4.08840	0.01330	6.09998
C	7	0.12712	1.99841	3.86013	0.01434	5.87288
C	8	-0.18102	1.99893	4.16443	0.01766	6.18102
C	9	-0.13005	1.99889	4.11085	0.02032	6.13005
C	10	-0.14322	1.99908	4.12568	0.01845	6.14322
C	11	-0.21335	1.99905	4.19407	0.02023	6.21335
C	12	-0.20755	1.99903	4.18899	0.01953	6.20755

C	13	0.00490	1.99905	3.97902	0.01703	5.99510
H	14	0.22154	0.00000	0.77682	0.00164	0.77846
H	15	0.22254	0.00000	0.77572	0.00174	0.77746
H	16	0.21846	0.00000	0.77971	0.00184	0.78154
H	17	0.21832	0.00000	0.77971	0.00196	0.78168
C	18	-0.55078	1.99898	4.31038	0.24142	6.55078
C	19	-0.19686	1.99891	4.14249	0.05546	6.19686
C	20	0.23494	1.99896	2.54778	1.21833	5.76506
C	21	-0.27715	1.99916	4.18036	0.09763	6.27715
C	22	-0.67573	1.99920	4.41677	0.25975	6.67573
C	23	-0.25288	1.99913	4.16994	0.08381	6.25288
H	24	0.21709	0.00000	0.77563	0.00728	0.78291
H	25	0.08539	0.00000	0.79358	0.12103	0.91461
H	26	0.21665	0.00000	0.78036	0.00300	0.78335
H	27	0.21156	0.00000	0.78171	0.00673	0.78844
H	28	0.21365	0.00000	0.78220	0.00415	0.78635
C	29	-0.59637	1.99928	4.58510	0.01198	6.59637
H	30	0.22342	0.00000	0.77499	0.00159	0.77658
H	31	0.21499	0.00000	0.78348	0.00153	0.78501
H	32	0.21636	0.00000	0.78210	0.00154	0.78364

===

* Total * -0.89253 39.98100 94.45018 2.46134 136.89253

1c

Energy E(B3LYP) = -3374.13922416 h, G^{298} = -3373.980636 h, μ=2.06 D

Cartesian coordinates, Å

N	atom	x	y	z
1	N	-2.851951	0.213130	0.000025
2	C	-4.012895	-0.528649	0.000033
3	C	-1.921468	-0.698193	0.000074
4	N	-3.811042	-1.825298	0.000075
5	O	-2.415525	-1.954298	0.000055
6	C	-0.526443	-0.526296	0.000068
7	C	0.671902	-0.382759	0.000064
8	C	2.080803	-0.207618	0.000032
9	C	2.634787	1.082433	-0.000061
10	C	2.934725	-1.321999	0.000095
11	C	4.010286	1.255412	-0.000091
12	C	4.310638	-1.151674	0.000065
13	C	4.833470	0.135889	-0.000027
14	H	1.985542	1.945460	-0.000109
15	H	2.517473	-2.318091	0.000167
16	H	4.431402	2.248794	-0.000162
17	H	4.963061	-2.011141	0.000114
18	C	-5.351663	0.073974	0.000012
19	C	-6.498345	-0.729552	-0.000002
20	C	-5.485630	1.465371	0.000007
21	C	-7.757232	-0.145323	-0.000020
22	C	-6.749427	2.044915	-0.000011
23	C	-7.886611	1.242746	-0.000024
24	H	-6.400718	-1.805387	0.000001
25	H	-4.601739	2.085452	0.000016
26	H	-8.638279	-0.771687	-0.000031
27	H	-6.844631	3.121720	-0.000014
28	H	-8.868851	1.694539	-0.000038
29	Br	6.734300	0.373653	-0.000068

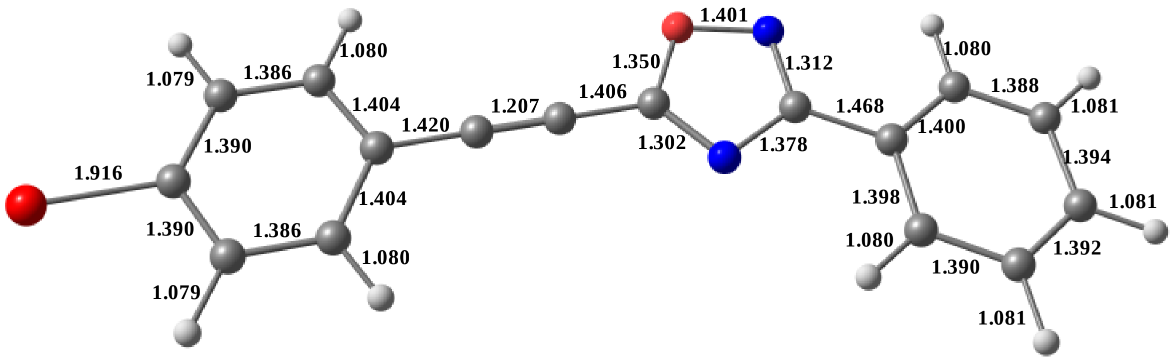

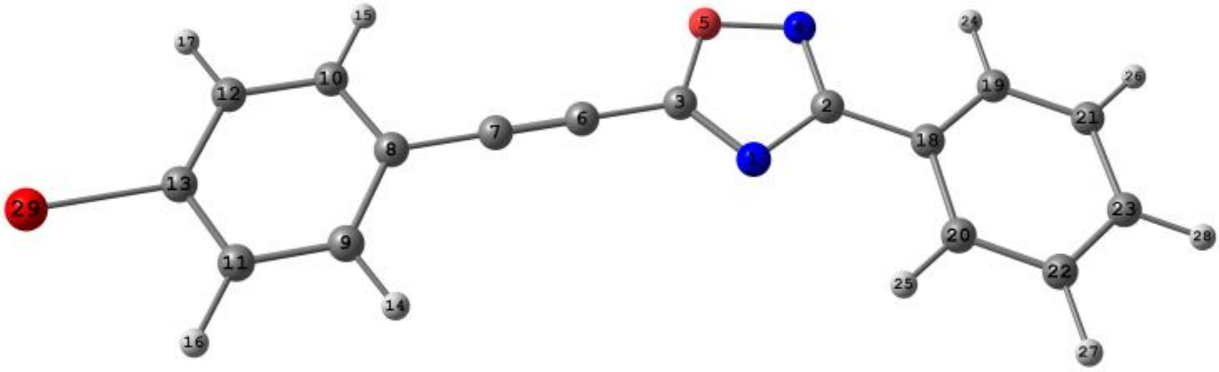

Summary of Natural Population Analysis:

Atom	No	Natural Charge	Natural Population: Core	Valence	Rydberg	Total
N	1	-0.51091	1.99935	5.47681	0.03475	7.51091
C	2	0.35244	1.99919	3.61346	0.03491	5.64756
C	3	0.48123	1.99907	3.47127	0.04842	5.51877
N	4	-0.17967	1.99934	5.14823	0.03210	7.17967
O	5	-0.32123	1.99972	6.29395	0.02756	8.32123
C	6	-0.08322	1.99831	4.07031	0.01460	6.08322
C	7	0.11500	1.99842	3.86492	0.02166	5.88500
C	8	-0.58209	1.99907	4.34976	0.23325	6.58209
C	9	-0.54766	1.99892	3.37866	1.17009	6.54766
C	10	0.34744	1.99894	2.52728	1.12634	5.65256
C	11	-0.28075	1.99895	4.21427	0.06752	6.28075
C	12	-0.68472	1.99898	4.43482	0.25093	6.68472
C	13	-0.14427	1.99858	4.05985	0.08584	6.14427
H	14	0.20802	0.00000	0.77651	0.01547	0.79198

H	15	0.02296	0.00000	0.83965	0.13740	0.97704
H	16	0.23580	0.00000	0.76113	0.00307	0.76420
H	17	0.22459	0.00000	0.76214	0.01327	0.77541
C	18	-0.11989	1.99877	4.09290	0.02823	6.11989
C	19	-0.16126	1.99892	4.14061	0.02173	6.16126
C	20	-0.54115	1.99893	3.32687	1.21535	6.54115
C	21	-0.20946	1.99917	4.18792	0.02237	6.20946
C	22	-0.23195	1.99920	4.19912	0.03363	6.23195
C	23	-0.18345	1.99914	4.16592	0.01839	6.18345
H	24	0.22270	0.00000	0.77496	0.00234	0.77730
H	25	0.21814	0.00000	0.77662	0.00524	0.78186
H	26	0.21777	0.00000	0.78038	0.00185	0.78223
H	27	0.21742	0.00000	0.78057	0.00201	0.78258
H	28	0.21644	0.00000	0.78186	0.00170	0.78356
Br	29	0.06106	27.99907	6.90888	0.03099	34.93894
* Total *		-1.64065	65.98003	92.95962	4.70101	163.64065

1d

Energy E(B3LYP) = -915.166299622 h, G^{298} = -914.965433 h, μ=5.56 D

Cartesian coordinates, Å

N	atom	x	y	z
1	N	-2.021061	0.243954	-0.000141
2	C	-3.150662	-0.542446	0.000000
3	C	-1.052691	-0.629986	-0.000200
4	N	-2.900258	-1.830454	0.000035
5	O	-1.499489	-1.905285	0.000040
6	C	0.331454	-0.404169	-0.000284
7	C	1.525378	-0.213057	-0.000352
8	C	2.921058	0.021876	-0.000205
9	C	3.426973	1.337392	-0.000216
10	C	3.828677	-1.047667	-0.000057
11	C	4.786137	1.565052	-0.000088
12	C	5.196889	-0.823822	0.000071
13	C	5.684577	0.487570	0.000058
14	H	2.741206	2.172164	-0.000330
15	H	3.456248	-2.061827	-0.000048
16	H	5.178779	2.571630	-0.000098
17	H	5.868396	-1.667149	0.000181
18	C	-4.512369	0.007220	0.000048
19	C	-5.626912	-0.840363	0.000137
20	C	-4.701624	1.392233	0.000005
21	C	-6.907825	-0.306172	0.000183
22	C	-5.987199	1.921735	0.000049
23	C	-7.091991	1.075653	0.000139
24	H	-5.487011	-1.911486	0.000169
25	H	-3.842618	2.046335	-0.000063
26	H	-7.763569	-0.966725	0.000251
27	H	-6.124602	2.993987	0.000015
28	H	-8.091233	1.488465	0.000174
29	O	6.996463	0.815584	0.000176
30	C	7.971254	-0.234306	0.000326
31	H	7.877774	-0.852708	0.892964
32	H	7.877949	-0.852799	-0.892268
33	H	8.935400	0.262758	0.000395

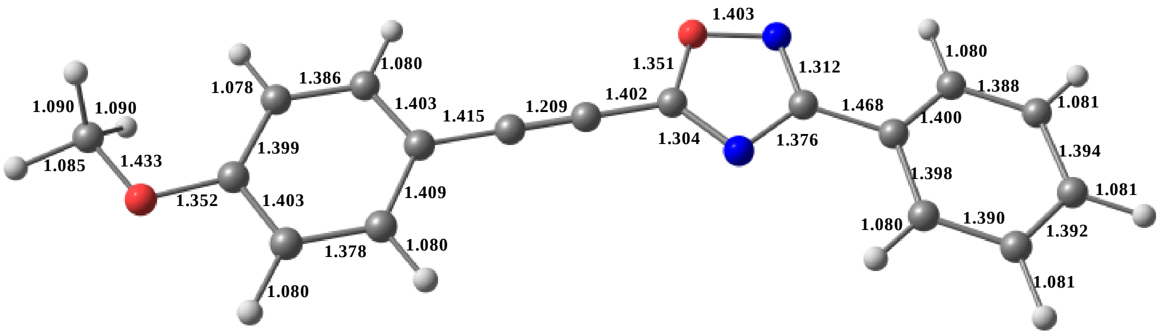

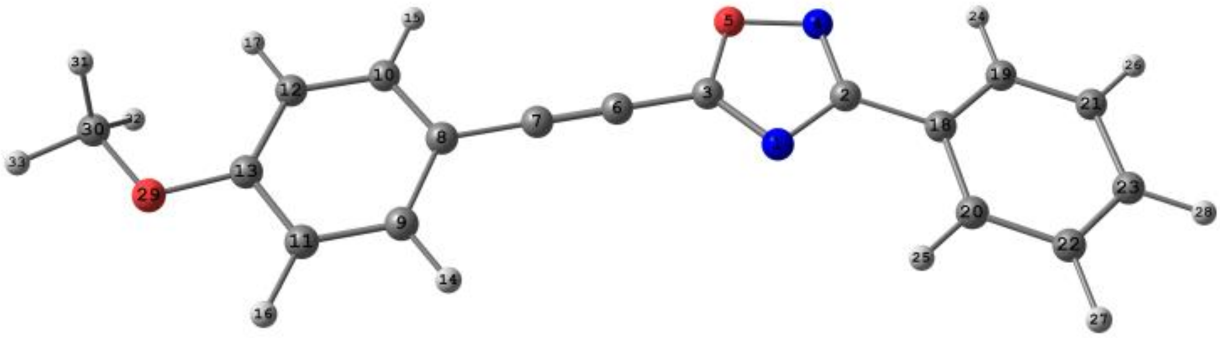

Summary of Natural Population Analysis:

Atom	No	Natural Charge	Core	Valence	Rydberg	Total
			Natural Population			
N	1	-0.55731	1.99938	5.50082	0.05711	7.55731
C	2	0.33186	1.99917	3.61588	0.05308	5.66814
C	3	0.26669	1.99902	3.53374	0.20054	5.73331
N	4	-0.19742	1.99933	5.15829	0.03979	7.19742
O	5	-0.35217	1.99973	6.31242	0.04003	8.35217
C	6	0.81033	1.99835	2.52225	0.66907	5.18967
C	7	-0.42056	1.99844	4.27564	0.14648	6.42056
C	8	-0.23378	1.99895	4.18874	0.04609	6.23378
C	9	-0.13872	1.99910	4.11688	0.02274	6.13872
C	10	-0.13016	1.99911	4.10828	0.02277	6.13016
C	11	-0.24494	1.99905	4.22524	0.02064	6.24494
C	12	-0.28672	1.99904	4.26901	0.01866	6.28672
C	13	0.35610	1.99881	3.62225	0.02284	5.64390

H	14	0.22259	0.00000	0.77400	0.00341	0.77741
H	15	0.22244	0.00000	0.77440	0.00316	0.77756
H	16	0.22936	0.00000	0.76847	0.00217	0.77064
H	17	0.23074	0.00000	0.76717	0.00209	0.76926
C	18	-0.56025	1.99898	4.31028	0.25099	6.56025
C	19	-0.20283	1.99892	4.14313	0.06079	6.20283
C	20	0.22160	1.99896	2.56204	1.21739	5.77840
C	21	-0.26290	1.99916	4.18016	0.08357	6.26290
C	22	-0.67932	1.99920	4.41706	0.26306	6.67932
C	23	-0.25588	1.99913	4.17037	0.08638	6.25588
H	24	0.21996	0.00000	0.77560	0.00444	0.78004
H	25	0.08315	0.00000	0.79441	0.12243	0.91685
H	26	0.21620	0.00000	0.78048	0.00333	0.78380
H	27	0.21185	0.00000	0.78184	0.00631	0.78815
H	28	0.21349	0.00000	0.78231	0.00420	0.78651
O	29	-0.54176	1.99970	6.51694	0.02512	8.54176
C	30	-0.20986	1.99923	4.19698	0.01365	6.20986
H	31	0.18226	0.00000	0.81553	0.00221	0.81774
H	32	0.18226	0.00000	0.81553	0.00221	0.81774
H	33	0.19914	0.00000	0.79961	0.00125	0.80086

===

* Total *	-0.87456	41.98077	99.37578	3.51801	144.87456

Aa

Energy E(B3LYP) = -801.021661821 h, G^{298} = -800.836342 h, μ=4.34 D

Cartesian coordinates, Å

N	atom	x	y	z
1	C	-6.146025	1.030357	0.074657
2	C	-5.916828	-0.300577	0.418328
3	C	-4.631311	-0.818350	0.380281
4	C	-3.564776	0.002903	-0.006073
5	C	-3.795478	1.338368	-0.352496
6	C	-5.086579	1.846854	-0.310081
7	C	-2.219450	-0.556953	-0.061116
8	N	-1.048104	0.173397	0.020804
9	C	-0.030914	-0.683365	-0.059988
10	O	-0.528818	-1.899763	-0.188858
11	N	-1.943041	-1.821996	-0.198132
12	C	1.322731	-0.429158	-0.026874
13	C	2.512297	-0.198603	-0.000884
14	C	3.892952	0.074094	0.025127
15	C	4.343530	1.399212	0.179717
16	C	5.702930	1.660765	0.202061
17	C	6.618063	0.616668	0.071151
18	C	6.177049	-0.697350	-0.082523
19	C	4.821087	-0.976632	-0.105868
20	H	3.625388	2.199552	0.279365
21	H	4.467556	-1.990095	-0.224263
22	H	6.051961	2.676205	0.320955
23	H	6.892027	-1.500861	-0.183796
24	H	7.678012	0.827620	0.088790
25	H	-4.447441	-1.845432	0.658469
26	H	-2.986186	1.977320	-0.676130
27	H	-6.739036	-0.932331	0.721770
28	H	-5.263794	2.877249	-0.581662
29	H	-7.149481	1.430433	0.108273
30	H	-0.956116	1.169233	0.167873

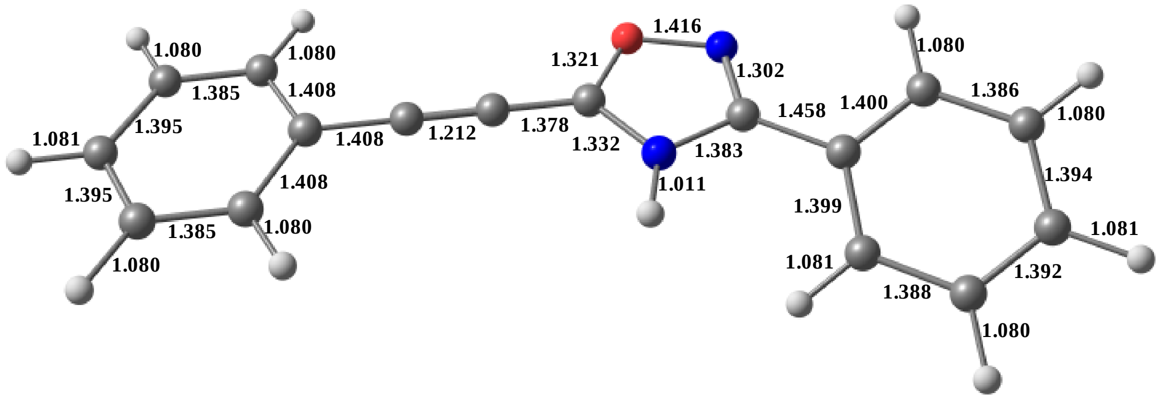

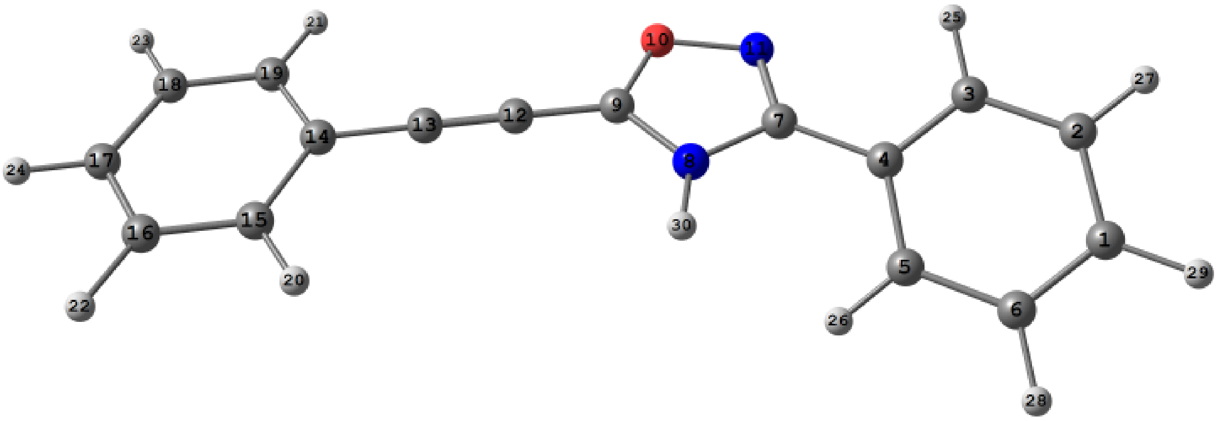

Summary of Natural Population Analysis:

Atom	No	Natural Charge	Natural Population Core	Valence	Rydberg	Total
C	1	-0.16248	1.99916	4.14414	0.01918	6.16248
C	2	-0.19999	1.99918	4.17999	0.02083	6.19999
C	3	-0.13612	1.99891	4.11722	0.02000	6.13612
C	4	-0.14130	1.99894	4.12347	0.01888	6.14130
C	5	-0.16020	1.99908	4.14387	0.01725	6.16020
C	6	-0.19307	1.99915	4.17383	0.02008	6.19307
C	7	0.42039	1.99917	3.55215	0.02829	5.57961
N	8	-0.49220	1.99920	5.47326	0.01974	7.49220
C	9	0.59775	1.99907	3.36958	0.03360	5.40225
O	10	-0.27169	1.99969	6.24591	0.02609	8.27169
N	11	-0.12865	1.99932	5.09668	0.03266	7.12865
C	12	-0.14917	1.99836	4.13484	0.01598	6.14917

C	13	0.23446	1.99847	3.74420	0.02287	5.76554
C	14	-0.55656	1.99894	4.35698	0.20065	6.55656
C	15	-0.15199	1.99907	4.11108	0.04185	6.15199
C	16	-0.27573	1.99914	4.17768	0.09891	6.27573
C	17	-0.19207	1.99915	4.12843	0.06450	6.19207
C	18	-0.64680	1.99919	4.41436	0.23325	6.64680
C	19	0.34646	1.99894	2.52395	1.13065	5.65354
H	20	0.22483	0.00000	0.76965	0.00552	0.77517
H	21	-0.00177	0.00000	0.85580	0.14597	1.00177
H	22	0.22605	0.00000	0.77133	0.00262	0.77395
H	23	0.21419	0.00000	0.77244	0.01337	0.78581
H	24	0.22149	0.00000	0.77558	0.00293	0.77851
H	25	0.23011	0.00000	0.76788	0.00201	0.76989
H	26	0.22214	0.00000	0.77607	0.00179	0.77786
H	27	0.22510	0.00000	0.77330	0.00159	0.77490
H	28	0.22564	0.00000	0.77271	0.00165	0.77436
H	29	0.22272	0.00000	0.77578	0.00151	0.77728
H	30	0.47729	0.00000	0.52024	0.00246	0.52271

===

* Total *	0.22883	37.98212	88.54239	2.24667	128.77117

Ab

Energy E(B3LYP) = -840.353469478 h, G^{298} = -840.144326 h, μ=4.25 D

Cartesian coordinates, Å

N	atom	x	y	z
1	N	-1.536330	0.162433	0.021276
2	C	-2.719337	-0.548559	-0.056151
3	C	-0.532263	-0.711815	-0.058432
4	N	-2.466000	-1.818437	-0.188616
5	O	-1.052606	-1.921160	-0.181590
6	C	0.823463	-0.481931	-0.028989
7	C	2.019942	-0.280597	-0.009484
8	C	3.403819	-0.047146	0.010411
9	C	3.901857	1.260655	0.170253
10	C	4.306477	-1.118745	-0.135904
11	C	5.266198	1.479176	0.184025
12	C	5.666726	-0.877563	-0.121129
13	C	6.172221	0.419579	0.041571
14	H	3.212897	2.085362	0.278565
15	H	3.928151	-2.122352	-0.262908
16	H	5.640278	2.486623	0.303632
17	H	6.352756	-1.704883	-0.240032
18	C	-4.055288	0.034183	-0.002069
19	C	-5.134114	-0.764563	0.396727
20	C	-4.264792	1.369382	-0.362332
21	C	-6.410653	-0.224779	0.433327
22	C	-5.546982	1.900083	-0.321473
23	C	-6.618701	1.105896	0.075642
24	H	-4.966487	-1.791407	0.685872
25	H	-3.445679	1.990507	-0.695724
26	H	-7.242285	-0.839186	0.746580
27	H	-5.707756	2.930223	-0.604079
28	H	-7.615202	1.523106	0.107951
29	H	-1.427711	1.156908	0.164167
30	C	7.653611	0.664858	0.085615
31	H	8.021480	0.564883	1.109815
32	H	8.193446	-0.056476	-0.525654
33	H	7.900157	1.669304	-0.253742

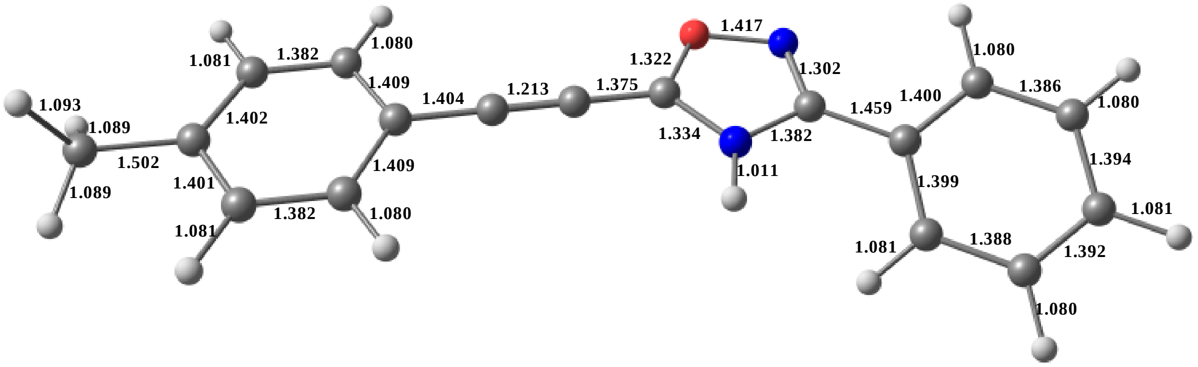

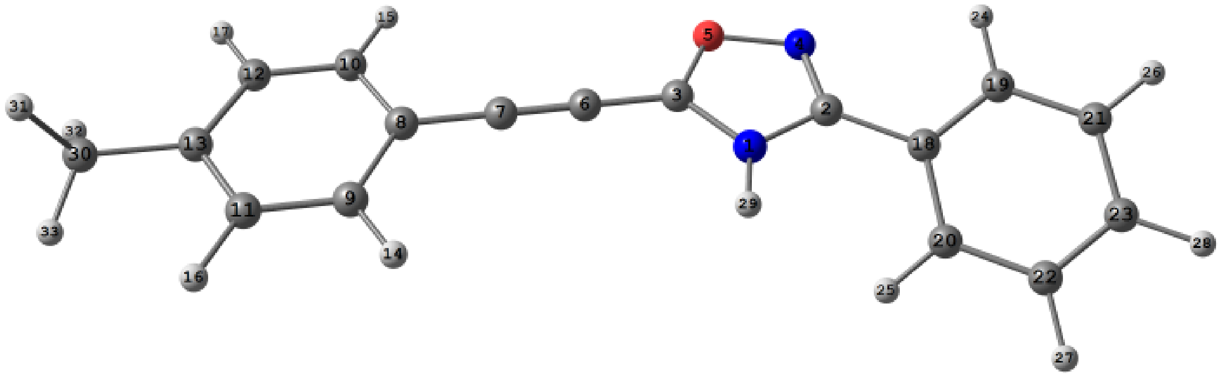

Summary of Natural Population Analysis:

Atom	No	Natural Charge	Core	Valence	Rydberg	Total
N	1	-0.49530	1.99920	5.47632	0.01978	7.49530
C	2	0.41951	1.99917	3.55278	0.02854	5.58049
C	3	0.59980	1.99906	3.37264	0.02850	5.40020
N	4	-0.13093	1.99932	5.09914	0.03248	7.13093
O	5	-0.27564	1.99970	6.24969	0.02625	8.27564
C	6	-0.15984	1.99837	4.14108	0.02040	6.15984
C	7	0.23275	1.99848	3.74371	0.02506	5.76725
C	8	-0.59226	1.99895	4.36854	0.22477	6.59226
C	9	0.30460	1.99894	2.51856	1.17790	5.69540
C	10	-0.16097	1.99907	4.10198	0.05992	6.16097
C	11	-0.65744	1.99908	4.42414	0.23422	6.65744
C	12	-0.26897	1.99902	4.18325	0.08669	6.26897
C	13	-0.00903	1.99905	3.94676	0.06322	6.00903

The header row of the table reads "Natural Population" above the Core, Valence, Rydberg and Total columns.

H	14	0.05831	0.00000	0.81246	0.12922	0.94169
H	15	0.22200	0.00000	0.77029	0.00771	0.77800
H	16	0.21702	0.00000	0.77556	0.00743	0.78298
H	17	0.22363	0.00000	0.77383	0.00254	0.77637
C	18	-0.14098	1.99894	4.12267	0.01936	6.14098
C	19	-0.13673	1.99890	4.11785	0.01997	6.13673
C	20	-0.16091	1.99908	4.14449	0.01735	6.16091
C	21	-0.20016	1.99918	4.18016	0.02083	6.20016
C	22	-0.19322	1.99915	4.17397	0.02009	6.19322
C	23	-0.16330	1.99916	4.14496	0.01919	6.16330
H	24	0.22994	0.00000	0.76805	0.00201	0.77006
H	25	0.22191	0.00000	0.77612	0.00197	0.77809
H	26	0.22492	0.00000	0.77349	0.00159	0.77508
H	27	0.22544	0.00000	0.77290	0.00165	0.77456
H	28	0.22256	0.00000	0.77593	0.00151	0.77744
H	29	0.47627	0.00000	0.52126	0.00247	0.52373
C	30	-0.62872	1.99928	4.58874	0.04070	6.62872
H	31	0.22918	0.00000	0.76775	0.00306	0.77082
H	32	0.21871	0.00000	0.77805	0.00324	0.78129
H	33	0.21758	0.00000	0.77916	0.00326	0.78242

===

* Total *	0.16972	39.98110	94.49628	2.35290	136.83028

Ac

Energy E(B3LYP) = -3374.55873046 h, G^{298} = -3374.387155 h, μ=10.05 D

Cartesian coordinates, Å

N	atom	x	y	z
1	N	-2.824592	0.139573	0.014644
2	C	-4.029166	-0.536450	-0.054732
3	C	-1.849527	-0.764438	-0.060935
4	N	-3.812206	-1.814536	-0.178323
5	O	-2.403427	-1.957603	-0.173451
6	C	-0.484398	-0.575135	-0.036760
7	C	0.716098	-0.413379	-0.022327
8	C	2.110748	-0.226353	-0.007459
9	C	2.648985	1.064480	0.145947
10	C	2.971310	-1.330741	-0.146875
11	C	4.020231	1.245894	0.160356
12	C	4.342238	-1.146756	-0.133293
13	C	4.852294	0.139017	0.020625
14	H	1.990245	1.913153	0.252906
15	H	2.559818	-2.321856	-0.264972
16	H	4.435444	2.234477	0.278130
17	H	5.004114	-1.991790	-0.240670
18	C	-5.347162	0.084805	-0.000028
19	C	-6.448277	-0.683499	0.398108
20	C	-5.518188	1.426483	-0.356950
21	C	-7.708474	-0.107029	0.437357
22	C	-6.784382	1.993855	-0.313440
23	C	-7.878159	1.230007	0.083001
24	H	-6.310344	-1.715353	0.685088
25	H	-4.682132	2.025128	-0.689522
26	H	-8.557164	-0.697779	0.750286
27	H	-6.915581	3.028868	-0.593461
28	H	-8.862107	1.675827	0.117448
29	H	-2.685203	1.131906	0.146433
30	Br	6.743511	0.391383	0.040461

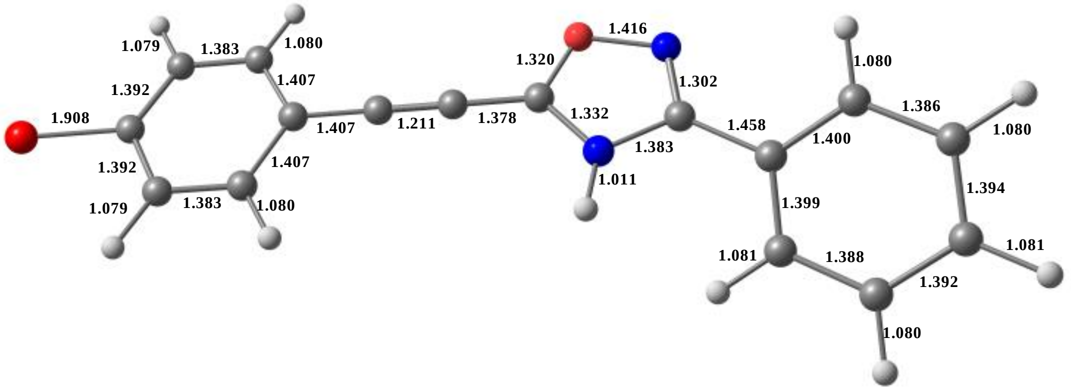

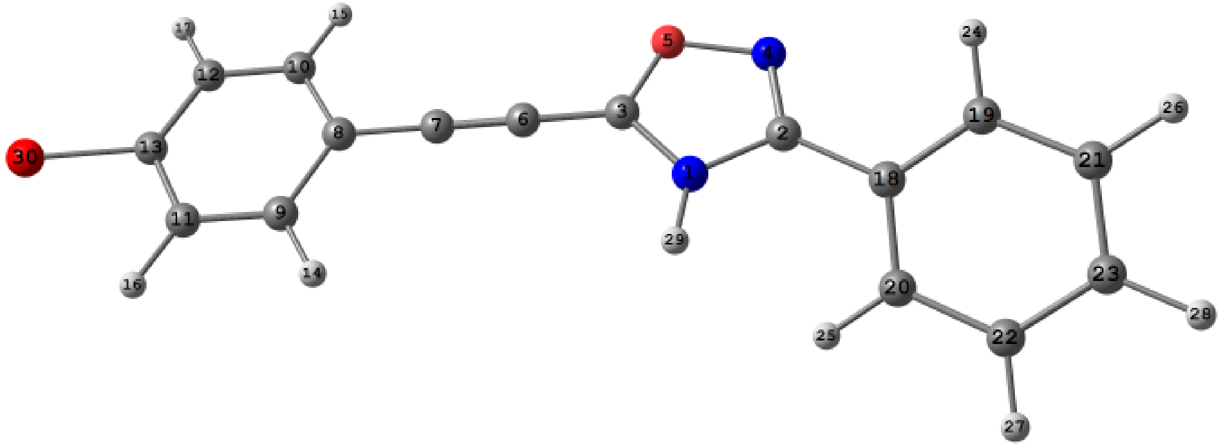

Summary of Natural Population Analysis:

Atom	No	Natural Charge	Natural Population Core	Valence	Rydberg	Total
N	1	-0.48828	1.99920	5.47068	0.01840	7.48828
C	2	0.41983	1.99917	3.55184	0.02916	5.58017
C	3	0.59838	1.99907	3.37012	0.03243	5.40162
N	4	-0.12754	1.99932	5.09524	0.03298	7.12754
O	5	-0.27087	1.99969	6.24453	0.02664	8.27087
C	6	-0.14086	1.99836	4.12668	0.01582	6.14086
C	7	0.22916	1.99846	3.74906	0.02332	5.77084
C	8	-0.55987	1.99894	4.35463	0.20630	6.55987
C	9	-0.13829	1.99907	4.09352	0.04571	6.13829
C	10	0.34062	1.99894	2.53039	1.13005	5.65938
C	11	-0.30397	1.99893	4.19979	0.10525	6.30397
C	12	-0.67490	1.99898	4.42966	0.24626	6.67490

C	13	-0.11177	1.99861	4.04353	0.06963	6.11177
H	14	0.23077	0.00000	0.76294	0.00629	0.76923
H	15	0.02206	0.00000	0.83838	0.13955	0.97794
H	16	0.24046	0.00000	0.75635	0.00319	0.75954
H	17	0.23068	0.00000	0.75775	0.01157	0.76932
C	18	-0.14186	1.99894	4.12371	0.01921	6.14186
C	19	-0.13604	1.99890	4.11695	0.02018	6.13604
C	20	-0.16023	1.99908	4.14370	0.01746	6.16023
C	21	-0.20000	1.99918	4.17997	0.02086	6.20000
C	22	-0.19322	1.99915	4.17383	0.02024	6.19322
C	23	-0.16214	1.99916	4.14382	0.01917	6.16214
H	24	0.23018	0.00000	0.76779	0.00203	0.76982
H	25	0.22214	0.00000	0.77600	0.00186	0.77786
H	26	0.22517	0.00000	0.77323	0.00160	0.77483
H	27	0.22572	0.00000	0.77263	0.00165	0.77428
H	28	0.22276	0.00000	0.77573	0.00151	0.77724
H	29	0.47739	0.00000	0.52003	0.00258	0.52261
Br	30	0.09057	27.99905	6.88218	0.02820	34.90943
* Total *		0.19603	65.98021	94.52466	2.29911	162.80397

Ad

Energy E(B3LYP) = -915.589370784 h, G^{298} = -915.374774 h, μ=2.76 D

Cartesian coordinates, Å

N	atom	x	y	z
1	N	-1.992122	0.157848	0.035144
2	C	-3.169934	-0.558903	-0.053945
3	C	-0.980350	-0.711578	-0.041140
4	N	-2.911481	-1.826797	-0.189558
5	O	-1.496235	-1.924625	-0.173027
6	C	0.370248	-0.474121	-0.002451
7	C	1.566671	-0.258963	0.018398
8	C	2.940635	-0.005159	0.037274
9	C	3.424266	1.314602	0.198777
10	C	3.863502	-1.059468	-0.109885
11	C	4.775064	1.558999	0.210447
12	C	5.221926	-0.814740	-0.100677
13	C	5.688753	0.499339	0.059386
14	H	2.724154	2.128922	0.312549
15	H	3.501617	-2.069540	-0.233122
16	H	5.157891	2.561437	0.332941
17	H	5.908508	-1.637282	-0.217261
18	C	-4.509029	0.018808	-0.008376
19	C	-5.588830	-0.784932	0.377130
20	C	-4.719900	1.354963	-0.363952
21	C	-6.867473	-0.249395	0.405204
22	C	-6.004114	1.881662	-0.331614
23	C	-7.076796	1.082349	0.052244
24	H	-5.420449	-1.812695	0.662572
25	H	-3.899841	1.980344	-0.686905
26	H	-7.699832	-0.867997	0.708185
27	H	-6.165571	2.912737	-0.610493
28	H	-8.074864	1.496286	0.077948
29	H	-1.890608	1.151766	0.184465
30	O	6.985049	0.843642	0.080655
31	C	7.986676	-0.174543	-0.075711
32	H	7.926042	-0.902034	0.732441
33	H	7.883255	-0.671246	-1.039396
34	H	8.936514	0.346276	-0.030726

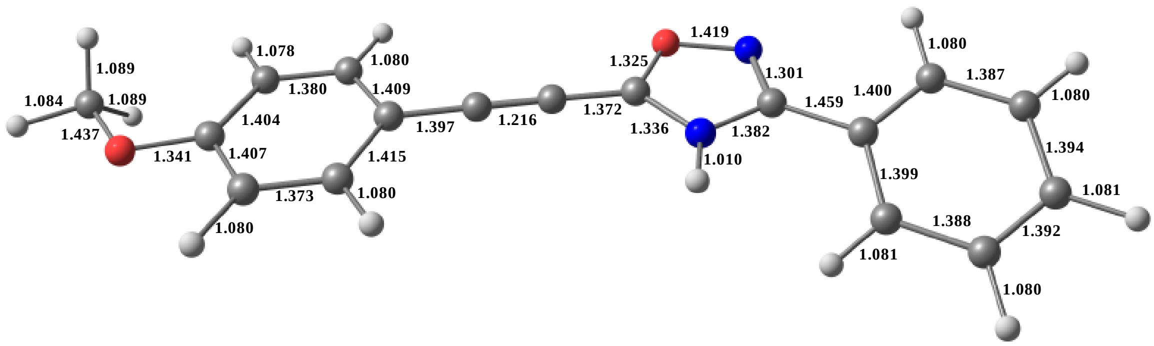

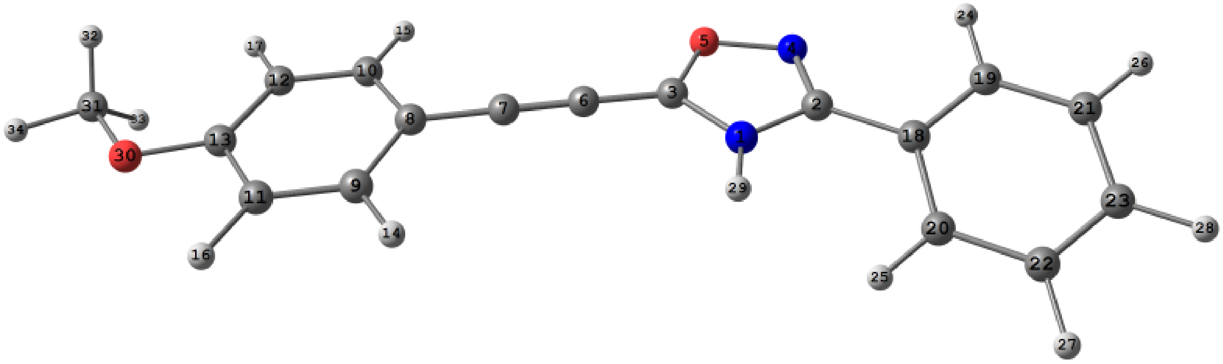

Summary of Natural Population Analysis:

Atom	No	Natural Charge	Core	Valence	Rydberg	Total
N	1	-0.52741	1.99923	5.49562	0.03256	7.52741
C	2	0.41971	1.99916	3.55211	0.02901	5.58029
C	3	0.33136	1.99903	3.46131	0.20830	5.66864
N	4	-0.13680	1.99932	5.10470	0.03278	7.13680
O	5	-0.31018	1.99971	6.27033	0.04015	8.31018
C	6	0.78411	1.99841	2.46518	0.75229	5.21589
C	7	-0.30207	1.99848	4.16121	0.14238	6.30207
C	8	-0.25035	1.99895	4.20723	0.04417	6.25035
C	9	-0.11532	1.99910	4.09459	0.02163	6.11532
C	10	-0.10210	1.99911	4.08133	0.02167	6.10210
C	11	-0.24166	1.99905	4.22228	0.02034	6.24166
C	12	-0.28376	1.99904	4.26661	0.01811	6.28376
C	13	0.38868	1.99885	3.59015	0.02232	5.61132
H	14	0.22855	0.00000	0.76800	0.00345	0.77145
H	15	0.22875	0.00000	0.76802	0.00322	0.77125

Natural Population (spans Core, Valence, Rydberg, Total)

H	16	0.23533	0.00000	0.76254	0.00212	0.76467
H	17	0.23641	0.00000	0.76155	0.00204	0.76359
C	18	-0.14039	1.99894	4.12167	0.01977	6.14039
C	19	-0.13759	1.99891	4.11878	0.01991	6.13759
C	20	-0.16163	1.99908	4.14521	0.01735	6.16163
C	21	-0.20034	1.99918	4.18035	0.02082	6.20034
C	22	-0.19343	1.99915	4.17416	0.02012	6.19343
C	23	-0.16448	1.99916	4.14611	0.01921	6.16448
H	24	0.22972	0.00000	0.76829	0.00199	0.77028
H	25	0.22162	0.00000	0.77651	0.00187	0.77838
H	26	0.22463	0.00000	0.77377	0.00160	0.77537
H	27	0.22516	0.00000	0.77318	0.00166	0.77484
H	28	0.22233	0.00000	0.77615	0.00151	0.77767
H	29	0.47373	0.00000	0.52280	0.00347	0.52627
O	30	-0.52390	1.99970	6.49923	0.02498	8.52390
C	31	-0.21151	1.99922	4.19897	0.01332	6.21151
H	32	0.18687	0.00000	0.81106	0.00207	0.81313
H	33	0.18686	0.00000	0.81107	0.00207	0.81314
H	34	0.20278	0.00000	0.79602	0.00121	0.79722
* Total *		1.02372	41.98076	100.42606	1.56945	143.97628

Ba

Energy E(B3LYP) = -801.403133047 h, G^{298} = -801.205854 h, μ=15.63 D

Cartesian coordinates, Å

N	atom	x	y	z
1	C	6.230683	-0.144477	0.005708
2	C	5.523660	-1.345574	0.000363
3	C	4.138196	-1.337791	0.016686
4	C	3.453625	-0.115369	0.038327
5	C	4.163697	1.090450	0.043501
6	C	5.551029	1.069952	0.027427
7	C	1.999567	-0.121454	0.070705
8	N	1.184673	0.941494	-0.297262
9	C	-0.064670	0.547409	-0.145914
10	O	-0.076354	-0.680108	0.292605
11	N	1.258418	-1.127329	0.445604
12	C	-1.269562	1.298469	-0.403172
13	C	-2.459449	0.802518	-0.253720
14	C	-3.697950	0.331071	-0.113302
15	C	-4.353595	-0.302849	-1.232315
16	C	-5.627280	-0.771893	-1.070549
17	C	-6.268012	-0.630482	0.172270
18	C	-5.647961	-0.019682	1.275865
19	C	-4.374824	0.463057	1.154844
20	H	-3.826602	-0.390240	-2.169741
21	H	-3.863210	0.939400	1.976750
22	H	-6.142525	-1.248025	-1.890330
23	H	-6.178498	0.065849	2.211542
24	H	-7.275922	-1.005589	0.283350
25	H	3.586733	-2.265988	-0.000764
26	H	3.651690	2.041254	0.080694
27	H	6.052681	-2.287121	-0.022910
28	H	6.098716	2.000821	0.036258
29	H	7.311261	-0.155833	-0.008744
30	H	1.483230	1.838225	-0.660759
31	H	-1.171290	2.328818	-0.734913

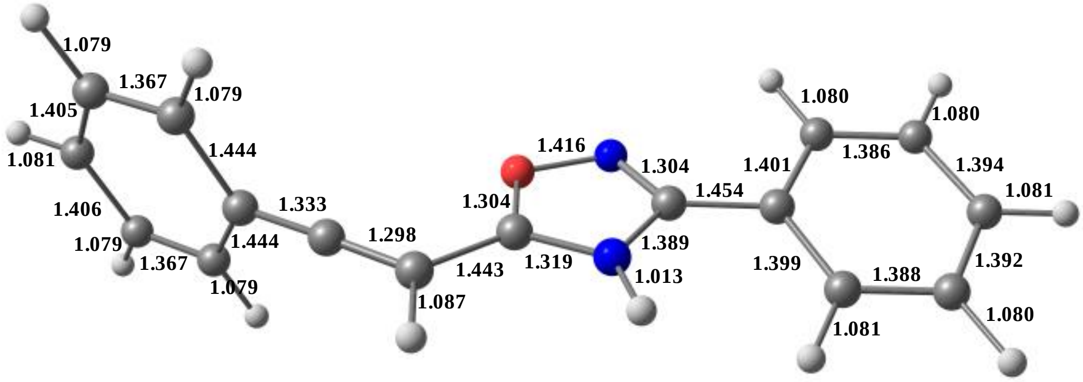

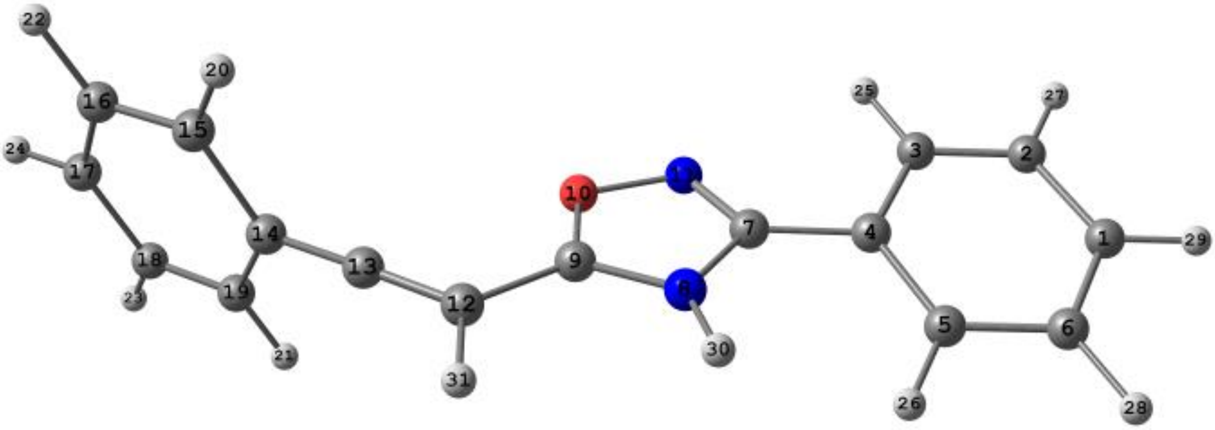

Summary of Natural Population Analysis:

Atom	No	Natural Charge	Core	Valence	Rydberg	Total
C	1	-0.15449	1.99916	4.13676	0.01856	6.15449
C	2	-0.19847	1.99918	4.17847	0.02083	6.19847
C	3	-0.13040	1.99891	4.11184	0.01965	6.13040
C	4	-0.15264	1.99897	4.13428	0.01940	6.15264
C	5	-0.14285	1.99890	4.12516	0.01879	6.14285
C	6	-0.19855	1.99918	4.17832	0.02105	6.19855
C	7	0.42799	1.99917	3.54551	0.02734	5.57201
N	8	-0.47393	1.99917	5.45832	0.01643	7.47393
C	9	0.68005	1.99921	3.29545	0.02529	5.31995
O	10	-0.26314	1.99966	6.23717	0.02631	8.26314
N	11	-0.10870	1.99933	5.07780	0.03158	7.10870
C	12	-0.34389	1.99881	4.32957	0.01551	6.34389

(Natural Population spans Core, Valence, Rydberg, Total.)

C	13	0.46855	1.99897	3.51609	0.01638	5.53145
C	14	-0.18897	1.99858	4.16905	0.02134	6.18897
C	15	-0.01064	1.99910	3.99359	0.01795	6.01064
C	16	-0.21086	1.99914	4.19243	0.01929	6.21086
C	17	0.02688	1.99920	3.95555	0.01837	5.97312
C	18	-0.21074	1.99914	4.19230	0.01930	6.21074
C	19	-0.01096	1.99910	3.99392	0.01795	6.01096
H	20	0.25130	0.00000	0.74726	0.00144	0.74870
H	21	0.25134	0.00000	0.74722	0.00144	0.74866
H	22	0.25199	0.00000	0.74647	0.00154	0.74801
H	23	0.25200	0.00000	0.74646	0.00154	0.74800
H	24	0.24141	0.00000	0.75729	0.00130	0.75859
H	25	0.23162	0.00000	0.76642	0.00196	0.76838
H	26	0.22114	0.00000	0.77709	0.00177	0.77886
H	27	0.22671	0.00000	0.77169	0.00160	0.77329
H	28	0.22739	0.00000	0.77100	0.00160	0.77261
H	29	0.22408	0.00000	0.77441	0.00151	0.77592
H	30	0.48530	0.00000	0.51219	0.00251	0.51470
H	31	0.33149	0.00000	0.66657	0.00194	0.66851

==

* Total *	2.00003	37.98284	89.60566	0.41147	127.99997

Bb

Energy E(B3LYP) = -840.742239366 h, G^{298} = -840.521133 h, μ=13.74 D

Cartesian coordinates, Å

N	atom	x	y	z
1	N	1.671893	-0.993076	0.297674
2	C	2.440222	0.110508	-0.049705
3	C	0.405205	-0.636378	0.198574
4	N	1.656939	1.104182	-0.364094
5	O	0.342098	0.607026	-0.190788
6	C	-0.768660	-1.431390	0.464704
7	C	-1.974583	-0.967760	0.320720
8	C	-3.218120	-0.524215	0.175661
9	C	-3.902876	0.104022	1.283262
10	C	-3.890984	-0.665363	-1.095718
11	C	-5.174328	0.553745	1.108485
12	C	-5.163172	-0.200646	-1.229875
13	C	-5.830625	0.416499	-0.142355
14	H	-3.391390	0.201453	2.228377
15	H	-3.370708	-1.137604	-1.914678
16	H	-5.698701	1.021038	1.928951
17	H	-5.677639	-0.301719	-2.173754
18	C	3.894514	0.157361	-0.053312
19	C	4.532575	1.401583	0.036404
20	C	4.649546	-1.016150	-0.156929
21	C	5.916819	1.463401	0.022314
22	C	6.035165	-0.941632	-0.170228
23	C	6.668665	0.294437	-0.080466
24	H	3.946463	2.303543	0.130838
25	H	4.173570	-1.981681	-0.249419
26	H	6.409981	2.421493	0.098626
27	H	6.617556	-1.847298	-0.255024
28	H	7.748083	0.347477	-0.088588
29	H	2.010325	-1.893956	0.611830
30	H	-0.630526	-2.457739	0.791886
31	C	-7.206542	0.942332	-0.310991
32	H	-7.806231	0.768569	0.581780
33	H	-7.146368	2.030031	-0.436463
34	H	-7.699947	0.530317	-1.186377

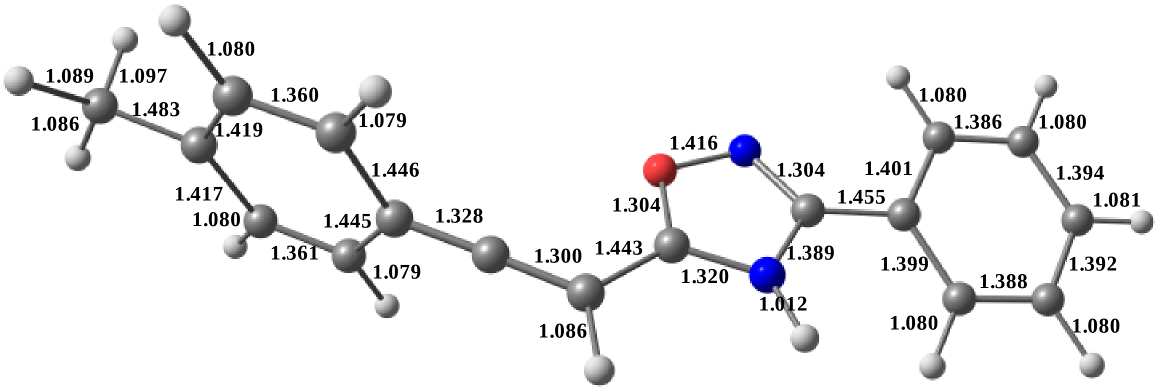

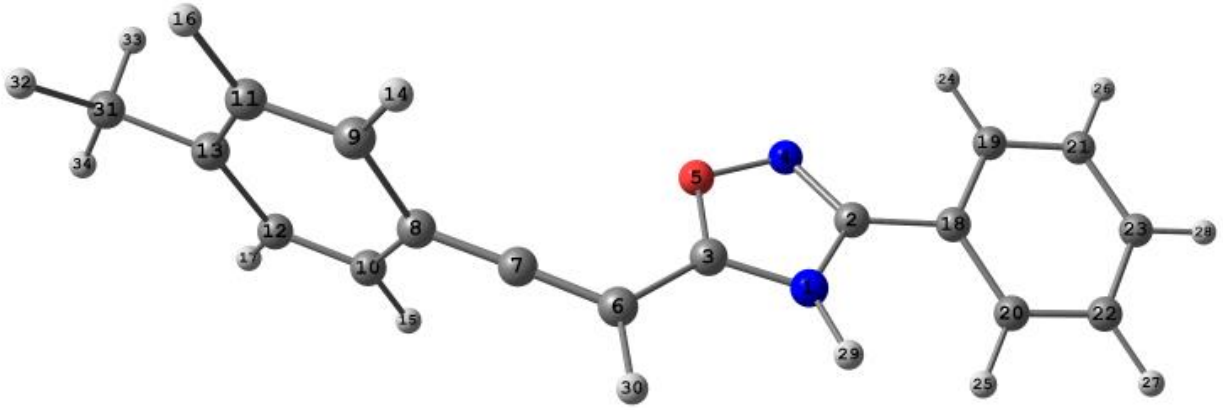

Summary of Natural Population Analysis:

Atom	No	Natural Charge	Core	Valence	Rydberg	Total
			Natural Population			
N	1	-0.47681	1.99917	5.46120	0.01644	7.47681
C	2	0.42713	1.99917	3.54627	0.02743	5.57287
C	3	0.68041	1.99921	3.29508	0.02531	5.31959
N	4	-0.11094	1.99932	5.08006	0.03155	7.11094
O	5	-0.26629	1.99966	6.24031	0.02632	8.26629
C	6	-0.34546	1.99881	4.33108	0.01556	6.34546
C	7	0.44110	1.99897	3.54361	0.01631	5.55890
C	8	-0.18262	1.99858	4.16299	0.02105	6.18262
C	9	-0.02047	1.99908	4.00338	0.01800	6.02047
C	10	-0.01430	1.99908	3.99722	0.01800	6.01430
C	11	-0.21127	1.99901	4.19280	0.01946	6.21127
C	12	-0.21390	1.99901	4.19554	0.01936	6.21390

C	13	0.21027	1.99911	3.77402	0.01660	5.78973
H	14	0.25025	0.00000	0.74825	0.00150	0.74975
H	15	0.25033	0.00000	0.74818	0.00149	0.74967
H	16	0.24882	0.00000	0.74940	0.00178	0.75118
H	17	0.24875	0.00000	0.74939	0.00186	0.75125
C	18	-0.15187	1.99897	4.13346	0.01944	6.15187
C	19	-0.13133	1.99891	4.11275	0.01967	6.13133
C	20	-0.14389	1.99890	4.12619	0.01880	6.14389
C	21	-0.19878	1.99917	4.17876	0.02084	6.19878
C	22	-0.19871	1.99918	4.17847	0.02106	6.19871
C	23	-0.15570	1.99916	4.13796	0.01858	6.15570
H	24	0.23140	0.00000	0.76664	0.00196	0.76860
H	25	0.22170	0.00000	0.77653	0.00177	0.77830
H	26	0.22649	0.00000	0.77190	0.00161	0.77351
H	27	0.22712	0.00000	0.77127	0.00161	0.77288
H	28	0.22387	0.00000	0.77462	0.00151	0.77613
H	29	0.48440	0.00000	0.51309	0.00251	0.51560
H	30	0.32383	0.00000	0.67421	0.00196	0.67617
C	31	-0.62691	1.99925	4.61294	0.01473	6.62691
H	32	0.24768	0.00000	0.75073	0.00159	0.75232
H	33	0.26970	0.00000	0.72878	0.00152	0.73030
H	34	0.23600	0.00000	0.76242	0.00158	0.76400

===

* Total *	2.00003	39.98173	95.58950	0.42874	135.99997

Bc

Energy E(B3LYP) = -3374.94122972 h, G^{298} = -3374.756547 h, μ=9.28 D

Cartesian coordinates, Å

N	atom	x	y	z
1	N	-2.934552	1.042252	0.187830
2	C	-3.658535	-0.133101	0.035048
3	C	-1.655755	0.718565	0.193503
4	N	-2.834217	-1.141047	-0.049520
5	O	-1.542360	-0.573828	0.062234
6	C	-0.517410	1.598939	0.306621
7	C	0.708961	1.176965	0.238186
8	C	1.978038	0.782539	0.157737
9	C	2.702837	0.409522	1.349444
10	C	2.633013	0.732539	-1.128111
11	C	4.002776	0.013970	1.247666
12	C	3.933209	0.332960	-1.204539
13	C	4.613853	-0.024822	-0.022740
14	H	2.204888	0.448828	2.305926
15	H	2.082342	1.013811	-2.012363
16	H	4.562243	-0.268023	2.125342
17	H	4.440214	0.290226	-2.155231
18	C	-5.108776	-0.228767	-0.028525
19	C	-5.712758	-1.485565	0.114708
20	C	-5.895360	0.907637	-0.248093
21	C	-7.091403	-1.596216	0.037062
22	C	-7.275619	0.785123	-0.321589
23	C	-7.873919	-0.463242	-0.180131
24	H	-5.105191	-2.360227	0.292674
25	H	-5.450612	1.883313	-0.379574
26	H	-7.557134	-2.564330	0.149812
27	H	-7.880549	1.663300	-0.493038
28	H	-8.949251	-0.554222	-0.238010
29	H	-3.308531	1.976447	0.296074
30	H	-0.703132	2.660908	0.441822
31	Cl	6.391031	-0.573432	-0.145056

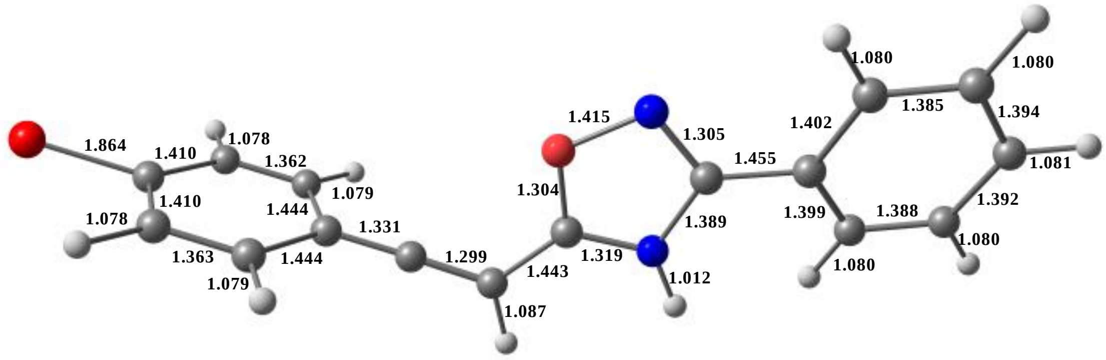

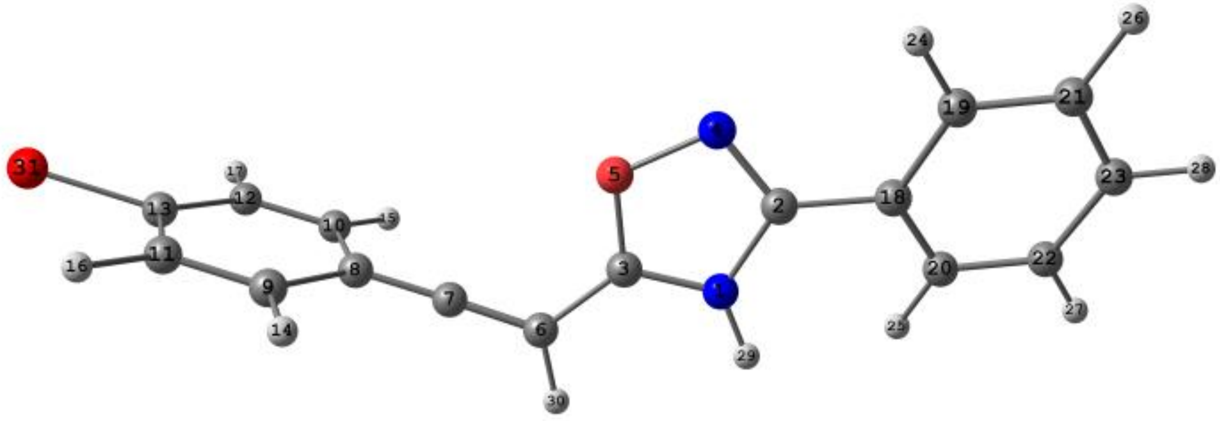

Summary of Natural Population Analysis:

Atom	No	Natural Charge	Natural Population: Core	Valence	Rydberg	Total
N	1	-0.47525	1.99917	5.45954	0.01654	7.47525
C	2	0.42338	1.99918	3.55030	0.02714	5.57662
C	3	0.67981	1.99921	3.29563	0.02535	5.32019
N	4	-0.11150	1.99933	5.08068	0.03149	7.11150
O	5	-0.26409	1.99966	6.23797	0.02646	8.26409
C	6	-0.34142	1.99881	4.32709	0.01552	6.34142
C	7	0.44525	1.99898	3.53947	0.01630	5.55475
C	8	-0.18329	1.99858	4.16352	0.02119	6.18329
C	9	-0.01279	1.99908	3.99570	0.01801	6.01279
C	10	-0.01204	1.99908	3.99499	0.01797	6.01204
C	11	-0.22755	1.99894	4.20560	0.02301	6.22755
C	12	-0.22810	1.99893	4.20613	0.02304	6.22810

C	13	0.02319	1.99876	3.94817	0.02988	5.97681
H	14	0.25613	0.00000	0.74240	0.00147	0.74387
H	15	0.25592	0.00000	0.74261	0.00147	0.74408
H	16	0.26251	0.00000	0.73550	0.00199	0.73749
H	17	0.26257	0.00000	0.73543	0.00199	0.73743
C	18	-0.13730	1.99870	4.11644	0.02215	6.13730
C	19	-0.13445	1.99891	4.11531	0.02023	6.13445
C	20	-0.14635	1.99891	4.12792	0.01952	6.14635
C	21	-0.19886	1.99917	4.17866	0.02103	6.19886
C	22	-0.19928	1.99918	4.17885	0.02125	6.19928
C	23	-0.15421	1.99916	4.13633	0.01872	6.15421
H	24	0.23161	0.00000	0.76637	0.00202	0.76839
H	25	0.21944	0.00000	0.77873	0.00183	0.78056
H	26	0.22659	0.00000	0.77179	0.00163	0.77341
H	27	0.22734	0.00000	0.77103	0.00163	0.77266
H	28	0.22403	0.00000	0.77446	0.00151	0.77597
H	29	0.48410	0.00000	0.51341	0.00249	0.51590
H	30	0.32649	0.00000	0.67155	0.00195	0.67351
Br	31	0.27811	27.99887	6.70773	0.01529	34.72189

===

* Total *	2.00002	65.98062	95.56929	0.45006	161.99998

Bd
Energy E(B3LYP) = -915.98915008 h, G^{298} = -915.762107 h, μ=12.79 D

Cartesian coordinates, Å

N	atom	x	y	z
1	N	2.118221	-0.953861	0.382915
2	C	2.873379	0.123613	-0.059298
3	C	0.846128	-0.635186	0.226106
4	N	2.079671	1.066240	-0.483954
5	O	0.769598	0.562820	-0.286252
6	C	-0.313988	-1.428235	0.547398
7	C	-1.529139	-1.012031	0.331132
8	C	-2.779614	-0.632007	0.125495
9	C	-3.504995	0.073818	1.164225
10	C	-3.443491	-0.923121	-1.132501
11	C	-4.790200	0.449627	0.966519
12	C	-4.724313	-0.540285	-1.316685
13	C	-5.425191	0.146652	-0.275960
14	H	-3.001574	0.288851	2.094252
15	H	-2.897515	-1.446093	-1.902226
16	H	-5.326468	0.970957	1.741996
17	H	-5.251353	-0.740657	-2.236955
18	C	4.327000	0.193155	-0.044323
19	C	4.948472	1.447075	-0.112760
20	C	5.098532	-0.972094	0.025459
21	C	6.331833	1.526734	-0.110218
22	C	6.483218	-0.880030	0.027797
23	C	7.099897	0.365854	-0.039386
24	H	4.350018	2.344940	-0.153871
25	H	4.636787	-1.948586	0.057731
26	H	6.811745	2.493426	-0.156943
27	H	7.077956	-1.780232	0.078292
28	H	8.178544	0.433188	-0.034936
29	H	2.466861	-1.812538	0.789540
30	H	-0.151003	-2.409422	0.981955
31	O	-6.653837	0.459351	-0.555119
32	C	-7.506007	1.149798	0.403001
33	H	-7.628762	0.534525	1.289451
34	H	-7.075171	2.116658	0.645015
35	H	-8.450518	1.270934	-0.111064

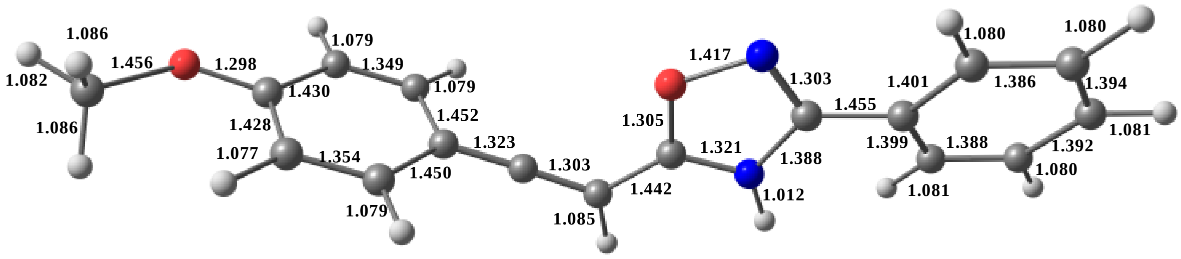

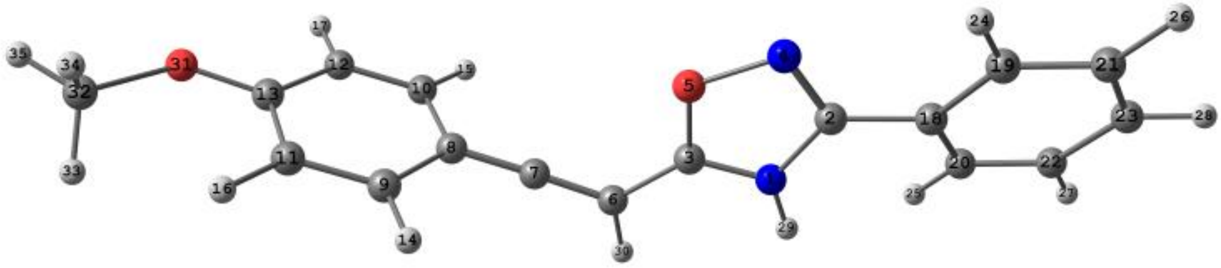

Summary of Natural Population Analysis:

Atom	No	Natural Charge	Natural Population: Core	Valence	Rydberg	Total
N	1	-0.48065	1.99917	5.46505	0.01643	7.48065
C	2	0.42576	1.99917	3.54770	0.02738	5.57424
C	3	0.68089	1.99921	3.29449	0.02541	5.31911
N	4	-0.11487	1.99932	5.08405	0.03149	7.11487
O	5	-0.26921	1.99966	6.24324	0.02631	8.26921
C	6	-0.34572	1.99883	4.33133	0.01556	6.34572
C	7	0.39595	1.99897	3.58863	0.01645	5.60405
C	8	-0.17453	1.99857	4.15447	0.02149	6.17453
C	9	-0.02384	1.99910	4.00651	0.01823	6.02384
C	10	-0.05159	1.99908	4.03460	0.01790	6.05159
C	11	-0.26756	1.99903	4.25125	0.01728	6.26756
C	12	-0.22694	1.99903	4.20783	0.02008	6.22694
C	13	0.51769	1.99899	3.46114	0.02218	5.48231
H	14	0.25103	0.00000	0.74748	0.00150	0.74897
H	15	0.25081	0.00000	0.74769	0.00149	0.74919
H	16	0.25729	0.00000	0.74084	0.00187	0.74271

H	17	0.25750	0.00000	0.74055	0.00195	0.74250
C	18	-0.15088	1.99897	4.13250	0.01941	6.15088
C	19	-0.13180	1.99891	4.11320	0.01970	6.13180
C	20	-0.14418	1.99890	4.12646	0.01882	6.14418
C	21	-0.19884	1.99918	4.17883	0.02084	6.19884
C	22	-0.19893	1.99918	4.17868	0.02107	6.19893
C	23	-0.15636	1.99916	4.13861	0.01859	6.15636
H	24	0.23123	0.00000	0.76680	0.00197	0.76877
H	25	0.22069	0.00000	0.77754	0.00178	0.77931
H	26	0.22627	0.00000	0.77212	0.00161	0.77373
H	27	0.22697	0.00000	0.77142	0.00161	0.77303
H	28	0.22371	0.00000	0.77477	0.00151	0.77629
H	29	0.48257	0.00000	0.51491	0.00252	0.51743
H	30	0.31323	0.00000	0.68478	0.00199	0.68677
O	31	-0.44187	1.99966	6.41793	0.02428	8.44187
C	32	-0.21493	1.99920	4.20210	0.01363	6.21493
H	33	0.20690	0.00000	0.79159	0.00151	0.79310
H	34	0.20653	0.00000	0.79196	0.00152	0.79347
H	35	0.21770	0.00000	0.78132	0.00098	0.78230

===

* Total *	2.00003	41.98127	101.56238	0.45632	143.99997
